# From Blueprints to Build: A Workshop for Developing a Clinical Coaching Program

**DOI:** 10.15766/mep_2374-8265.11548

**Published:** 2025-09-26

**Authors:** Alice Walz, Taryn Hill, Matthew W. Thomas, Caroline Rassbach, Leslie Dingeldein, Jessica Goldstein, H. Mollie Grow, Monique Naifeh, Erin Powell, Rebecca Blankenburg

**Affiliations:** 1 Associate Professor, Department of Pediatrics, Medical University of South Carolina; 2 Assistant Professor, Department of Pediatrics, Johns Hopkins All Children's Hospital; 3 Assistant Professor, Department of Pediatrics, West Virginia University School of Medicine; 4 Clinical Professor, Department of Pediatrics, Stanford School of Medicine; 5 Assistant Professor, Department of Pediatrics, Case Western Reserve University School of Medicine; 6 Associate Professor, Department of Neurology, University of Minnesota Medical School; 7 Professor of Pediatrics, Department of Pediatrics, University of Washington/Seattle Children's Hospital; 8 Professor of Pediatrics, Department of Pediatrics, University of Oklahoma Health Sciences Center; 9 Pediatric Intensivist, St Luke's Children's Hospital; † Co-primary author

**Keywords:** Direct Observation (Clinical), Clinical Teaching/Bedside Teaching, Mentoring/Coaching

## Abstract

**Introduction:**

Clinical coaching is an effective way to support learner skill attainment and professional development. Multiple health professions societies encourage coaching as an integral strategy that should be incorporated in training programs. However, a structured, systematic approach to developing coaching programs does not exist. This workshop presented a framework to help individual programs develop their own clinical coaching program.

**Methods:**

We developed a 105-minute workshop that includes both didactics and facilitated small- and large-group discussions where participants used an innovative Coaching Program Blueprint to design and build or refine a model for their own coaching program. We used an anonymous postworkshop survey to assess the effectiveness of workshop content, format, and resources.

**Results:**

The workshop was presented at two national (2022, 2023) and two international (2022, 2023) academic medical conferences to audiences of various roles in medical education, but with a primary focus on resident and fellow education. Postworkshop survey results included a total of 124 participants. The participants identified learning about different structures and stages of clinical coaching programs as the most valuable aspect of the workshop and appreciated the Blueprint as a clear framework for program structure and organization. Participants suggested that the workshop could be improved by allowing more time to expand on the main four coaching structural principles.

**Discussion:**

The Coaching Program Blueprint is a useful resource to help educational leaders develop a clinical coaching program. Ongoing education differentiating coaching from mentoring or advising programs is important to consider when developing a clinical coaching program.

## Educational Objectives

By the end of this session, learners will be able to:

1. Identify the purpose and benefits of coaching in health professions education.
2. Examine the main structural components, facilitators, and barriers in developing a coaching program.
3. Design a coaching program blueprint relevant to your institution.

## Introduction

Coaching is a tool to support trainee development that is distinct from advising or mentoring and emphasizes the principle that learning is never finished and that to reach one's maximum potential, accurate self-reflection, external feedback, and goal setting are essential. Long used by other professions, coaching has a new and growing role in health professions education (HPE). In 2021, the Coalition of Physician Accountability recommended that training programs utilize coaching to optimize the UME to GME transition for developing learners.^1^ In addition, the American Medical Association (AMA) and Canadian Royal College of Physicians and Surgeons recommend coaching as a modality for improving clinical skills.^2,3^ There are different types of coaching, including executive coaching aimed at enhancing leadership skills, and academic coaching aimed at ensuring trainee's academic success or career exploration. Clinical coaching supports professional growth and clinical skill development by aiding medical learners in setting clinical performance goals, with coaches then directly observing learners in a variety of clinical care areas, guiding their self-reflection, providing feedback, and facilitating future goal setting.

Coaching has been shown to improve physicians’ self-reflection practices, goal setting, and professionalism, and also can reduce burnout.^4–6^ Additionally, the literature supports the use of coaching techniques for the development of clinical skills, procedural skills, communication, teaching skills, well-being, and professional development.^7–15^ Several curricula available on *MedEdPORTAL* promote the use of coaching techniques for teaching these skills^16,17^; however, they do not provide guidance for programs to systematically develop and/or optimize their own HPE coaching program. It is imperative for the success of a coaching program to have well-defined program aims, structure, funding strategy, and faculty and coachee development, as well as a rigorous evaluation process.^18,19^ Variation exists in the aims and implementation of coaching programs in HPE,^20^ and a structured, systematic approach to formation of a coaching program for interested institutions is needed. Our program builds on prior work published in *MedEdPORTAL* by providing an innovative Coaching Program Blueprint, designed to provide structure and allow for program customization to meet the unique coaching needs of an institution. This workshop is designed to guide participants through the key logistical questions of the who, what, where, when, and how of coaching program development, enabling them to use the Coaching Program Blueprint at their own home institution.

## Methods

### Workshop Development

We developed the workshop following Kern's 6-step model for curriculum development.^21^ For Kern's Steps 1 and 2, we performed an extensive literature search on coaching in medical education, including peer-reviewed manuscripts and curricula (including those in *MedEdPORTAL*). For Step 3, we created three learning objectives to best assist our participants in designing, building, and refining a coaching program using our Coaching Program Blueprint, as follows:

• *Objective 1*: Identify the purpose and benefits of coaching in HPE.
• *Objective 2*: Examine the main structural components, facilitators, and barriers in developing a coaching program.
• *Objective 3*: Design a coaching program blueprint that aligns with your program's goals.

For Step 4, we used interactive educational strategies, including polls, skits, brief didactics with PowerPoint presentation, small-group discussions, large-group Q&A panels, and information sharing via the platform Padlet. For Step 5, we presented the workshop at national and international medical conferences targeted toward faculty within HPE. For Step 6, we developed and distributed a postworkshop evaluation based on the workshop objectives and content. The blueprint and workshop design were supported by Kolb's Learning Cycle^22^—Concrete Experience (feeling), Reflective Observation (watching), Abstract Conceptualization (thinking), and Active Experimentation (doing). The concrete experience came from the didactic sessions, with reflective observation promoted by the small-group facilitation, with abstract conceptualization and active experimentation taking place as participants developed their own plans using the blueprint.

### Facilitators

The workshop author group consists of educational leaders who direct clinical coaching programs at several pediatric residency programs across the US, and who developed, facilitated, and evaluated this coaching blueprint and workshop. As facilitators we all had robust experiences in coaching program development at our respective academic institutions. As a group we defined specific roles ahead of time, including leading didactics, acting out coaching scenarios, leading small-group discussions, and facilitating large-group panels. We created the blueprint to guide educators who may be novice to clinical coaching. However, for institutions who do not have access to leaders in the field of coaching, facilitators may consider additional background training through workshops on coaching provided by the AMA Accelerating Change in Medical Education Academic Coaching team, or other clinical coaching workshops.

### Audience

We delivered this to an audience of various educational leaders, including faculty, educational leaders, and HPE administrators who currently have, are developing, or are planning to develop a coaching program. We envision the use of this workshop and Coaching Program Blueprint locally, as a framework for educational leaders to brainstorm and devise their own coaching program.

### The Workshop

This 105-minute workshop was created to support participants in developing and refining a coaching program in HPE. The workshop includes a structured process that incorporates foundational knowledge in coaching, interactive discussion, and hands-on activities to ensure participants leave with a plan for future implementation at their home institution. The workshop is presented using the Clinical Coaching Program Development PowerPoint presentation (Appendix A) and follows the agenda outlined in the facilitator guide (Appendix B).^23–26^ The facilitator guide (Appendix B) additionally includes instructions for preworkshop preparation, and the detailed agenda includes instructions on when workshop materials are to be used. The format was intentionally designed with flexible timing and successfully adapted to fit 75–105-minute time frames based on individual conference requirements. Our recommended workshop includes an extended time frame that offers the advantage of deeper engagement in the main small-group activity, where participants can actively brainstorm, share experiences, and learn new coaching frameworks and resources.

The workshop begins with a brief introduction in which facilitators highlight the 3 main learning objectives. Participants complete polling questions to reflect on their personal and professional experiences with coaching, followed by a think-pair-share activity where they discuss effective coaching experiences. A 10-minute didactic session follows, which introduces core coaching concepts, the benefits of coaching in HPE, and key recommendations. This didactic segment compares and contrasts the academic roles of coaching, mentoring, and advising, while highlighting the role of coaching to support learner development. The coaching cycle is introduced as an iterative process, paralleling the Plan, Do, Study, Act (PDSA) cycle for continuous process improvement in quality improvement work. The coaching cycle parallels the PDSA cycle's message of continuous and ongoing study to promote growth and development, with an emphasis on goal setting, direct observation, self-reflection, and feedback. The Ask, Discuss, Ask, Plan Together (ADAPT) framework^27^ is presented as a structured coaching tool used in the coaching cycle.

The workshop transitions to two brief role-playing skits (5 minutes each) that juxtapose a directive feedback approach with a coaching approach, simulating a clinical debrief after a failed lumbar puncture attempt. Full written scripts are provided for the skits (Appendix C). The first scenario depicts a directive approach, with a focus on feedback only, while the second scenario demonstrates effective coaching by applying the ADAPT framework to guide thoughtful questioning, promote self-reflection, and support goal setting. This segment is followed by a 10-minute facilitated large-group discussion, during which participants review structures and aims of different coaching programs at Stanford University, Johns Hopkins All Children's Hospital (JHACH), the University of Washington, and the University of Kentucky. Through guided questions and open dialogue, participants collaboratively examine different coaching models and share how these approaches might inform the development of their own programs.

The next segment of the workshop introduces participants to phase 1 of the Coaching Program Blueprint. Participants are provided an editable coaching blueprint (Appendix D) and guided through a brief didactic presentation (10 minutes) reviewing the who, what, where and when, and how questions and barriers related to coaching program development. Participants are encouraged to take notes and begin populating their blueprint with ideas for their own coaching program. Participants then engage in a second think-pair-share activity (5 minutes) to reflect on why coaching is meaningful to them, recording their insights in the blueprint's relevant section, followed by a brief group share-out discussion.

The workshop advances to phase 2 of the Coaching Program Blueprint, in which participants flip to the back side of the blueprint (Appendix D) and are introduced to the four main structural components: 1) program structure, 2) tools for feedback and facilitated reflection, 3) faculty development, and 4) evaluation and outcomes. These four structural components serve as the core building blocks for thoughtful design, implementation, and ongoing refinement of a coaching program. Appendix A outlines the main aims and core resources to guide discussion and planning for each structural component, preparing participants for the next small-group activity. Participants then engage in a 30-minute small-group activity, selecting their top two structural components to explore in depth. Four tables will be set up, each representing one of the four structural components, allowing participants to rotate between tables in 15-minute intervals. Each table is led by one or two facilitators who have reviewed relevant materials, including Example Coaching Program Blueprint-JHACH (Appendix E), Example Coaching Program Blueprint-Medical University of South Carolina (Appendix F), Example Coaching Program Blueprint-Stanford (Appendix G), Structured Clinical Observation Tool (Appendix H), and Resident Self-Reflection and Goal Setting Form (Appendix I). Facilitators remain at their assigned table while participants rotate between the structural components. The goal is to engage in facilitated discussion using guided questions, explore key frameworks, and review practical resources to conceptualize their coaching program. If workshop time can be extended, additional rotations can be added in 15-minute increments to allow participants to discuss all four structural components.

The workshop concludes with a 10-minute large-group report-out discussion, during which participants share unifying themes, key insights, identified barriers, and preliminary action items for coaching program development. Participants may also present their initial design proposals for the main structural components they worked on in small groups. In the final 5 minutes, participants complete a commitment-to-action exercise by writing one SMART (Specific, Measurable, Achievable, Relevant, Time-bound) goal specific to their coaching program building. Participants end the workshop with completion of the postworkshop survey (Appendix J).

### Evaluation and Analysis

At the workshop conclusion, individuals participating in the workshop at the Association of Pediatric Program Directors (APPD) Conference, International Conference on Residency Education (ICRE), and the Pediatric Academic Societies’ (PAS) Meeting were provided with an anonymous postworkshop evaluation form that was created by the authors, and which had been used previously in workshops and publications (Appendix J). This postworkshop evaluation used a combination of six statements with response ratings on a 5-point Likert scale (1 = *strongly disagree*, 5 = *strongly agree*), and four open-ended questions to evaluate effectiveness of the workshop and, specifically, the Coaching Program Blueprint.

The ACGME mandated their own survey for the workshop conducted at their conference. The ACGME survey had eight evaluation statements with response ratings based on a 5-point Likert scale (1 = *very low/I disagree*, 2 = *low*, 3 = *moderate/I'm neutral*, 4 = *high*, 5 = *very high/I agree*). The ACGME survey also included three open-ended qualitative questions targeting workshop successes and areas of improvement. Participants at the ACGME conference were required to complete the ACGME survey, and therefore we did not administer our own survey at that conference, to avoid survey fatigue by workshop participants.

Descriptive statistics were used to describe the evaluations. Content analysis was used to code the open-ended questions. Using inductive content analysis, two coders (Alice Walz and Rebecca Blankenburg) independently assigned codes to each of the open-ended answers and then met to reconcile any differences. The two coders then generated categories, or themes, which were shared with the full study team, who reviewed and refined the themes for clarity. In addition, following our workshop presentations, we recorded field notes of our reflections on the questions asked by the audience and areas that required further explanation. These field notes were triangulated with the content analysis to improve the reliability of the data.

The workshop was submitted for review by the Stanford University Institutional Review Board for use across the multiple sites and was determined to not meet the definition of human subject research (protocol 73310; decision date: December 10, 2024).

## Results

We presented this workshop at two national academic conferences, the APPD conference in May 2022 and the ACGME conference in February 2023, and at two international academic conferences, the ICRE in October 2022 and the PAS Meeting in May 2023. A total of 79 participants completed the ACGME evaluation and 45 participants completed our postworkshop survey (23 at APPD, 11 at ICRE, 11 at PAS). ACGME conference attendees (*N* = 79) rated the overall quality of the workshop very highly on the 5-point Likert scale (mean score 4.54/5; Table 1). Moreover, participants from the APPD, ICRE, and PAS conferences agreed that the workshop met its objectives (mean score 4.78), was a valuable use of time (mean score 4.85), and provided useful resources (mean score 4.94; Table 2). Our participants highlighted wanting to learn more about coaching and wanting to provide faculty development about coaching as commitments to action following our workshop.

**Table 1.**
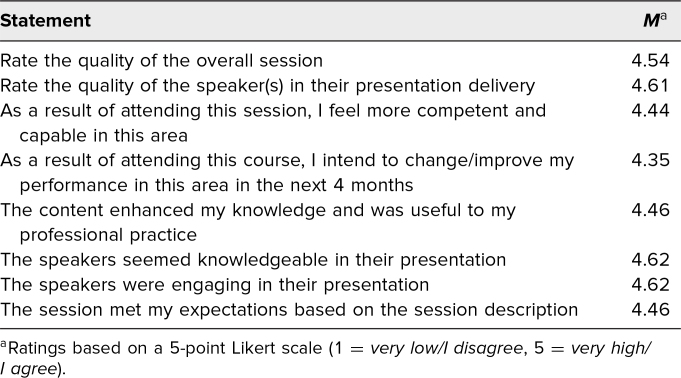
Quantitative Evaluations of the Clinical Coaching Workshop From ACGME Conference Attendees (*N* = 79)

**Table 2.**
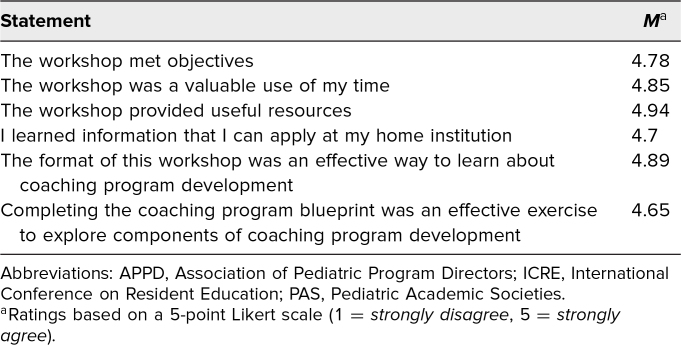
Quantitative Evaluations of Effectiveness of the Clinical Coaching Workshop From Attendees at the APPD Conference, ICRE, and PAS Meeting (*N* = 45)

The 124 participants responding to the postworkshop surveys wrote 119 comments to the open-ended questions. We asked participants to identify the most valuable aspect of the workshop and analyzed the results using conventional content analysis, where several themes emerged (summarized in Table 3). Participants appreciated the shared experience of hearing the clear examples of different coaching programs and their evolution in structure. Participants valued the resources provided in the workshop and found that the blueprints were helpful for brainstorming and organization. Finally, participants indicated that the small groups created opportunities for robust discussions, allowed participants to learn about the different structures and stages of various clinical coaching programs, and provided a forum to ask questions. One respondent commented that this workshop provided “a roadmap for getting started.” Another participant stated, “I left with concrete steps to start a pilot coaching team at my program.”

**Table 3.**
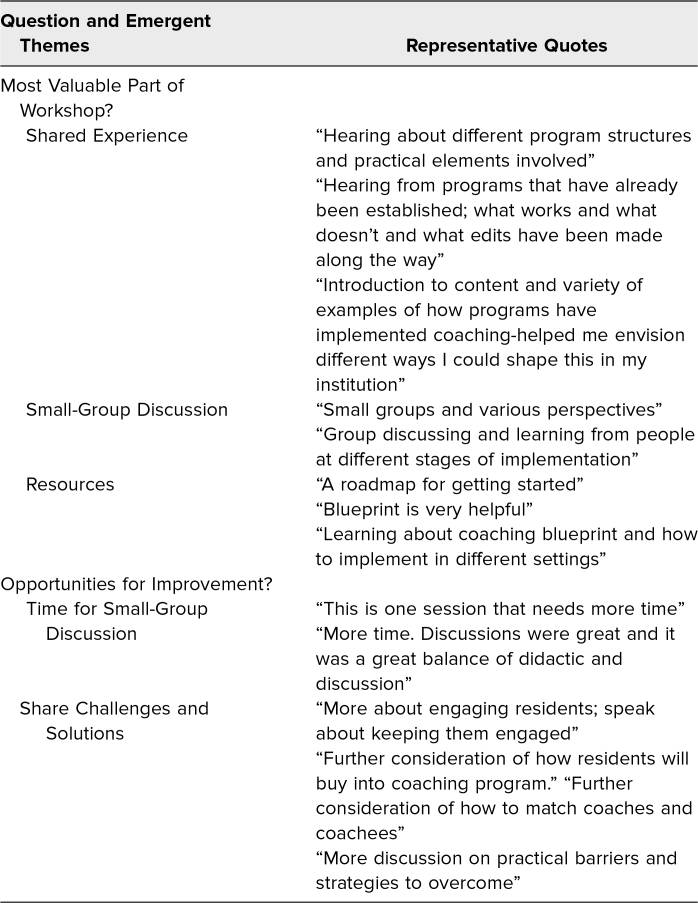
Summary of Participant Responses to Open-Ended Questions on the Postworkshop Surveys

Participants were additionally asked what adjustments could be made to improve the workshop for future presentations. Themes emerged related to time allocation for small-group discussion, as well as increased discussion of barriers and solutions to program implementation. These data were consistent with our field notes and informed future iterations of our workshop, when additional time was allocated to small-group discussion and the final large-group activity was expanded to specifically include discussion of key insights and identified barriers.

## Discussion

We developed and implemented a workshop to teach educational leaders how to systematically develop a clinical coaching program. We presented this workshop at two national academic conferences and two international academic conferences, where it was well received by participants with diverse roles in medical education. Overall, participants indicated that the workshop was an effective format and that the Coaching Program Blueprint was a useful resource to develop the structure of a clinical coaching program.

The focus of our workshop is on building or refining a coaching program that addresses an intended skill development. In the course of presenting multiple iterations of this workshop, we learned several lessons that helped us to optimize the workshop content and delivery. Most importantly, we found that workshop attendees entered our sessions with variable baseline understanding of the difference between coaching, mentoring, and advising. Thus, we found it important that our workshop first highlight the ways in which coaching is a different and complementary approach to enhancing learner development, before coaching program development is discussed. To that end, the final iteration of our workshop includes dedicated time highlighting these differences. Additionally, a common focus of discussion in our workshop surrounded models of funded versus unfunded support of coaching programs. We found that participants particularly appreciated discussion surrounding unfunded strategies for clinical coach support, including the use of volunteer faculty, such as professor emeriti or adjunct faculty, use of clinical fellows, providing coaching bonuses, providing educational value units, or building coaching into existing structures such as primary continuity clinic attendings. As the ACGME begins implementing requirements for funding of core faculty, it may be an additional opportunity to support clinical coach faculty.

Finally, because the most helpful part of the workshop for many attendees was the small-group discussions exploring facets of program development, we found that it was beneficial to prime attendees to the wide applicability of coaching for learner development by sharing specific examples of the use of coaching in different program structures. We also found it helpful to group participants by stage of coaching program development.

This workshop fills a needed gap by providing a roadmap for developing clinical coaching programs in HPE at a time when multiple organizations, including the AAMC, ACGME, AMA, and Canadian Royal College of Physicians and Surgeons, have called for the use of coaching broadly in these professions. This workshop and blueprint are generalizable across the HPE field and guide participants to build their coaching programs based on their unique goals for their clinical coaching programs. We recommend bringing the faculty coaches into the discussion and co-creation of program structure using the Coaching Program Blueprint to increase the diversity of perspectives and engagement of the entire coaching team.

Our workshop has several limitations. Attendees at our workshop may have had an existing interest in coaching or coaching program development, and thus there may be an element of selection bias in the feedback that we received. Additionally, without the total number of workshop participants at each of the conferences, we are unable to calculate a survey response rate, which could impact interpretation of the data. However, as we received similar feedback across audiences from multiple specialties and educational roles, we feel that our data are generalizable. We were able to measure the intended impact of our workshop by asking participants to commit to actions in program development. However, we are unable to fully assess the actual impact of our work. Future studies should examine actual behavior change related to program development and assess what additional resources educators would find useful to help streamline coaching program development.

## Appendices


Coaching Program Development.pptxFacilitator Guide.docxCoaching Skits.docxEditable Coaching Program Blueprint.docxExample Coaching Program Blueprint - JHACH.docxExample Coaching Program Blueprint - MUSC.docxExample Coaching Program Blueprint - Stanford.docxStructured Clinical Observation Coaching Tool.docxResident Self-Reflection and Goal Setting Form.docxPostworkshop Survey.docx
*All appendices are peer reviewed as integral parts of the Original Publication.*
