# Supplementary material for: From Blueprints to Build: A Workshop for Developing a Clinical Coaching Program
Source: MedEdPORTAL. 2025 Sep 26;21:11548. doi: 10.15766/mep_2374-8265.11548 (PMC12464251; doi:10.15766/mep_2374-8265.11548)
Supplement: Supplementary file 1 — Coaching Program Development.pptxFacilitator Guide.docxCoaching Skits.docxEditable Coaching Program Blueprint.docxExample Coaching Program Blueprint - JHACH.docxExample Coaching Program Blueprint - MUSC.docxExample Coaching Program Blueprint - Stanford.docxStructured Clinical Observation Coaching Tool.docxResident Self-Reflection and Goal Setting Form.docxPostworkshop Survey.docx [file mep_2374-8265.11548-s001.zip › H. Structured Clinical Observation Coaching Tool.docx]

**Instructions:** Facilitators can showcase this form as a coaching tool at the table for “Tools for Feedback and Facilitated Reflection." Faculty coaches use this form to document notes during and after a clinical observation of their coachee.

| HISTORY-TAKING, RELATIONSHIP SKILLS, & PERSONAL MANNER |
| --- |
|  |
| PHYSICAL EXAM |
|  |
| PATIENT AND FAMILY INTERACTION, RELATIONSHIP SKILLS, AND PERSONAL MANNER |
|  |
| PRESENTATION TO INPATIENT PRECEPTOR |
|  |
| REFLECTIONS & THOUGHTS |
|  |
| OPPORTUNITIES FOR GROWTH |
|  |

**THINGS TO THINK ABOUT**

***The following points can be used to guide feedback about the observation experience.***

***You do not need to comment on all of the points below.***

**HISTORY-TAKING**

Introduces self and explains role

Positions self and others to facilitate communication

Uses open-ended and pointed questions appropriately. Avoids leading questions.

Gathers essential information and inquires thoroughly about the presenting problem/chief complaint (allows patient/parent to talk).

Deliberately explores the patient’s/family’s perspective, *e.g.,* explanations for symptoms, fears or worries, expectations, or priorities for treatment.

Performs efficient history, in a hypothesis-directed manner, that targets the reason for patient’s visit and initial differential diagnosis.

Avoids interrupting.

Uses straight-forward language (*e.g.*, avoids using medical jargon).

**PHYSICAL EXAM**

Performs Infection Prevention practices appropriately, *e.g.*, hand hygiene or contact/droplet/airborne precautions.

Explains to the patient or child what s/he is doing.

Demonstrates correct exam technique.

Includes exam elements appropriate for the patient's age.

Modifies approach and sequence to exam in response to changes in patient’s level of cooperation or comfort.

Performs efficient exam that targets reason for patient’s visit and initial differential diagnosis.

**PATIENT AND FAMILY INTERACTION, RELATIONSHIP SKILLS, AND PERSONAL MANNER**

Appropriate body language, eye contact, tone of voice, and facial expressions. Appears comfortable, unhurried, and appropriately confident.

Gathers patient/family input and includes patient/family in discussion as appropriate.

Explains actual or potential diagnoses.

Explains management plan and rationale using accurate and evidence-based information.

Checks for understanding of patient/family.

Responds well to patient and family questions.

Conveys support, concern, and respect (verbally and nonverbally).

Recognizes ambiguity and uncertainty are a part of clinical medicine, and utilizes appropriate resources to address these issues.

Demonstrates sensitivity and responsiveness to patient’s diversity, *e.g.,* age, gender, culture, race, religion, disabilities, or sexual orientation.

**PRESENTATION TO INPATIENT PRECEPTOR**

Presents appropriate amount of detail in history, including relevant events and patient and family concerns.

Presents appropriate amount of detail in physical exam, including relevant vital signs and growth parameters.

Presents diagnostic studies with an appropriate amount of detail

Includes pertinent lab results only. Avoids a detailed list of labs with extraneous information.

Includes trends or comparisons with past findings.

Creates an accurate, concise, prioritized assessment, including

Uses semantic qualifiers (acute/chronic, mild/severe)

Severity and course of illness (better/worse/same)

Provides an appropriately prioritized differential diagnosis for new/undiagnosed problems.

Creates a prioritized problem list with assessment/plan for each problem.

Creates a reasonable plan.

Incorporates evidence-based medicine into plan.

Incorporates patient or family preferences into plan when appropriate.

Incorporates considerations of cost-awareness and risk-benefit analysis in patient care, as appropriate.

Clarifies or summarizes action items.

Well-organized presentation.

**REFLECTIONS AND THOUGHTS / OPPORTUNITIES FOR GROWTH**

The coach should allow the learner to lead that discussion as much as possible.

The learner should reflect on the experience and provide self-directed feedback, and the coach should provide commentary regarding the learner’s self-directed feedback.

The coach should provide feedback regarding his/her observation of the learner’s encounter and allow the resident to reflect upon that feedback.

From this discussion, the learner and coach should discuss opportunities for future growth.
