# Supplementary material for: From Blueprints to Build: A Workshop for Developing a Clinical Coaching Program
Source: MedEdPORTAL. 2025 Sep 26;21:11548. doi: 10.15766/mep_2374-8265.11548 (PMC12464251; doi:10.15766/mep_2374-8265.11548)
Supplement: Supplementary file 1 — Coaching Program Development.pptxFacilitator Guide.docxCoaching Skits.docxEditable Coaching Program Blueprint.docxExample Coaching Program Blueprint - JHACH.docxExample Coaching Program Blueprint - MUSC.docxExample Coaching Program Blueprint - Stanford.docxStructured Clinical Observation Coaching Tool.docxResident Self-Reflection and Goal Setting Form.docxPostworkshop Survey.docx [file mep_2374-8265.11548-s001.zip › I. Resident Self-Reflection and Goal Setting Form.docx]

**Appendix I: Resident Self-Reflection & Goal Setting Form**

| **Clinical Goals**  (Inpatient, outpatient, career path) | **Professional Goals**  (Scholarship, leadership) | **Educational Goals**  (Conferences, Boards studying) | **Personal Goals**  (Wellness, work-life balance) |
| --- | --- | --- | --- |
|  |  |  |  |

**Areas of strength and pride:**

**Areas of challenge and/or areas that continue to develop:**
