# Supplementary material for: From Blueprints to Build: A Workshop for Developing a Clinical Coaching Program
Source: MedEdPORTAL. 2025 Sep 26;21:11548. doi: 10.15766/mep_2374-8265.11548 (PMC12464251; doi:10.15766/mep_2374-8265.11548)
Supplement: Supplementary file 1 — Coaching Program Development.pptxFacilitator Guide.docxCoaching Skits.docxEditable Coaching Program Blueprint.docxExample Coaching Program Blueprint - JHACH.docxExample Coaching Program Blueprint - MUSC.docxExample Coaching Program Blueprint - Stanford.docxStructured Clinical Observation Coaching Tool.docxResident Self-Reflection and Goal Setting Form.docxPostworkshop Survey.docx [file mep_2374-8265.11548-s001.zip › J. Postworkshop Survey.docx]

Strongly disagree Strongly agree

Workshop met objectives 1 2 3 4 5

Workshop was a valuable use of my time 1 2 3 4 5

Workshop provided useful resources 1 2 3 4 5

I learned information that I can apply 1 2 3 4 5

at my home institution

The format of this workshop was an effective way 1 2 3 4 5

to learn about coaching program development

Completing the coaching program blueprint was an 1 2 3 4 5

effective exercise to explore components of coaching

program development

1. What was/were the most valuable aspect(s) of this workshop?
2. What other information do you wish had been provided?
3. After completing this workshop training, I commit to implementing or developing the following 2 coaching program components in the next 6 weeks.
4. How can this coaching program development workshop be improved for next time?
