# Supplementary material for: From Blueprints to Build: A Workshop for Developing a Clinical Coaching Program
Source: MedEdPORTAL. 2025 Sep 26;21:11548. doi: 10.15766/mep_2374-8265.11548 (PMC12464251; doi:10.15766/mep_2374-8265.11548)
Supplement: Supplementary file 1 — Coaching Program Development.pptxFacilitator Guide.docxCoaching Skits.docxEditable Coaching Program Blueprint.docxExample Coaching Program Blueprint - JHACH.docxExample Coaching Program Blueprint - MUSC.docxExample Coaching Program Blueprint - Stanford.docxStructured Clinical Observation Coaching Tool.docxResident Self-Reflection and Goal Setting Form.docxPostworkshop Survey.docx [file mep_2374-8265.11548-s001.zip › A. Coaching Program Development.pptx]

## Slide 1
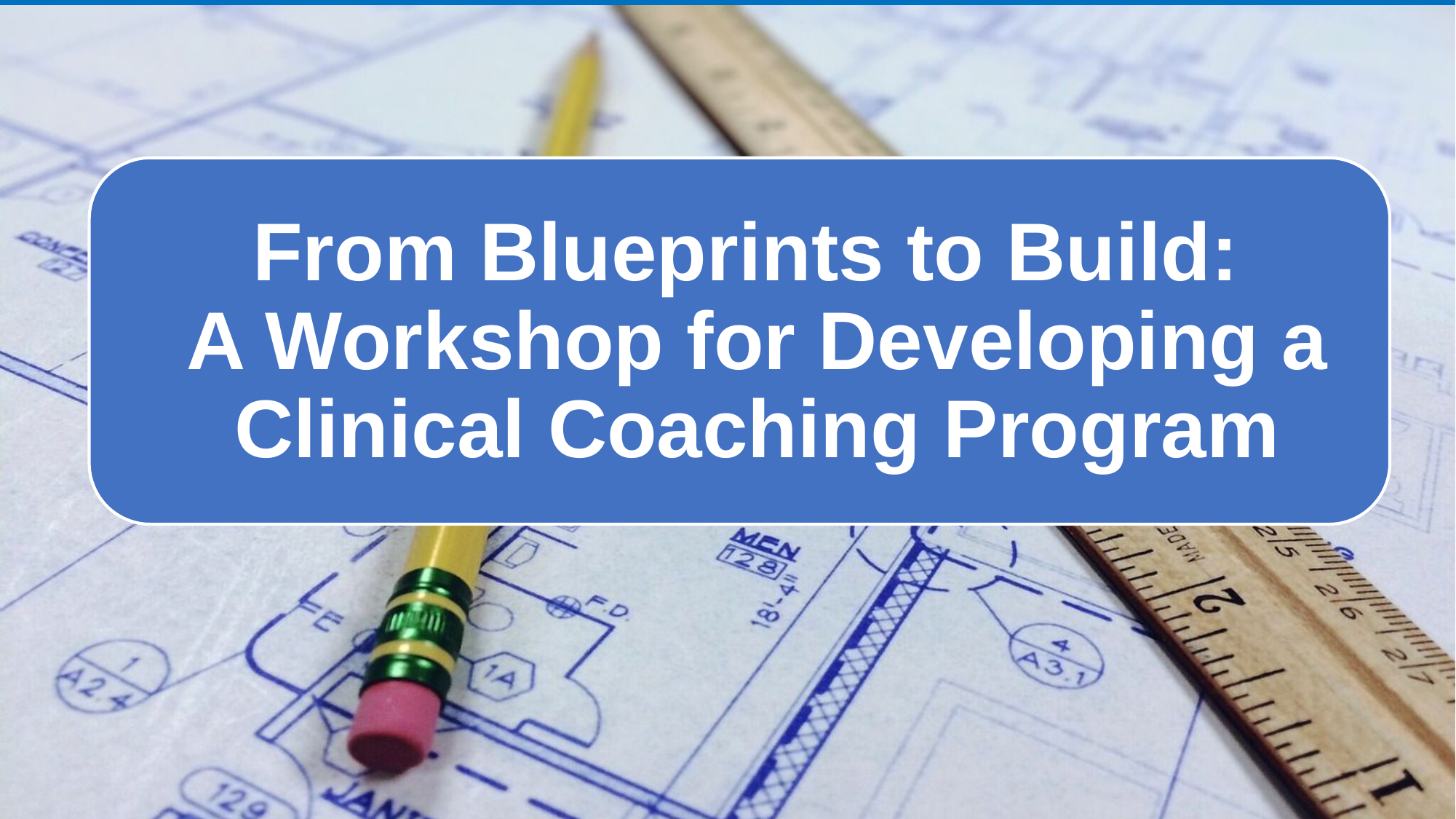

From Blueprints to Build:
A Workshop for Developing a Clinical Coaching Program

## Slide 2
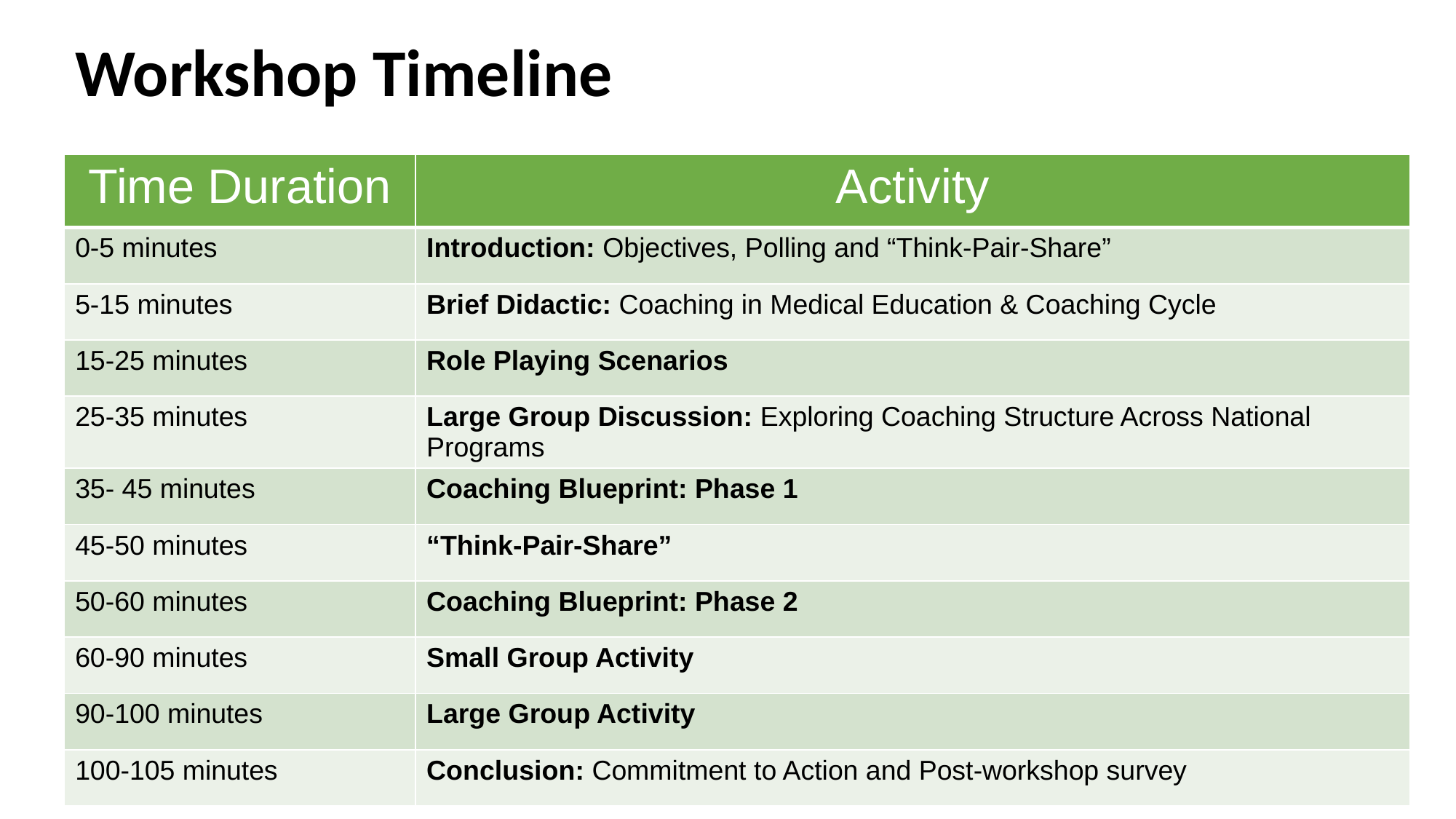

# Workshop Timeline
| Time Duration | Activity |
| --- | --- |
| 0-5 minutes | Introduction: Objectives, Polling and “Think-Pair-Share” |
| 5-15 minutes | Brief Didactic: Coaching in Medical Education & Coaching Cycle |
| 15-25 minutes | Role Playing Scenarios |
| 25-35 minutes | Large Group Discussion: Exploring Coaching Structure Across National Programs |
| 35- 45 minutes | Coaching Blueprint: Phase 1 |
| 45-50 minutes | “Think-Pair-Share” |
| 50-60 minutes | Coaching Blueprint: Phase 2 |
| 60-90 minutes | Small Group Activity |
| 90-100 minutes | Large Group Activity |
| 100-105 minutes | Conclusion: Commitment to Action and Post-workshop survey |

## Slide 3
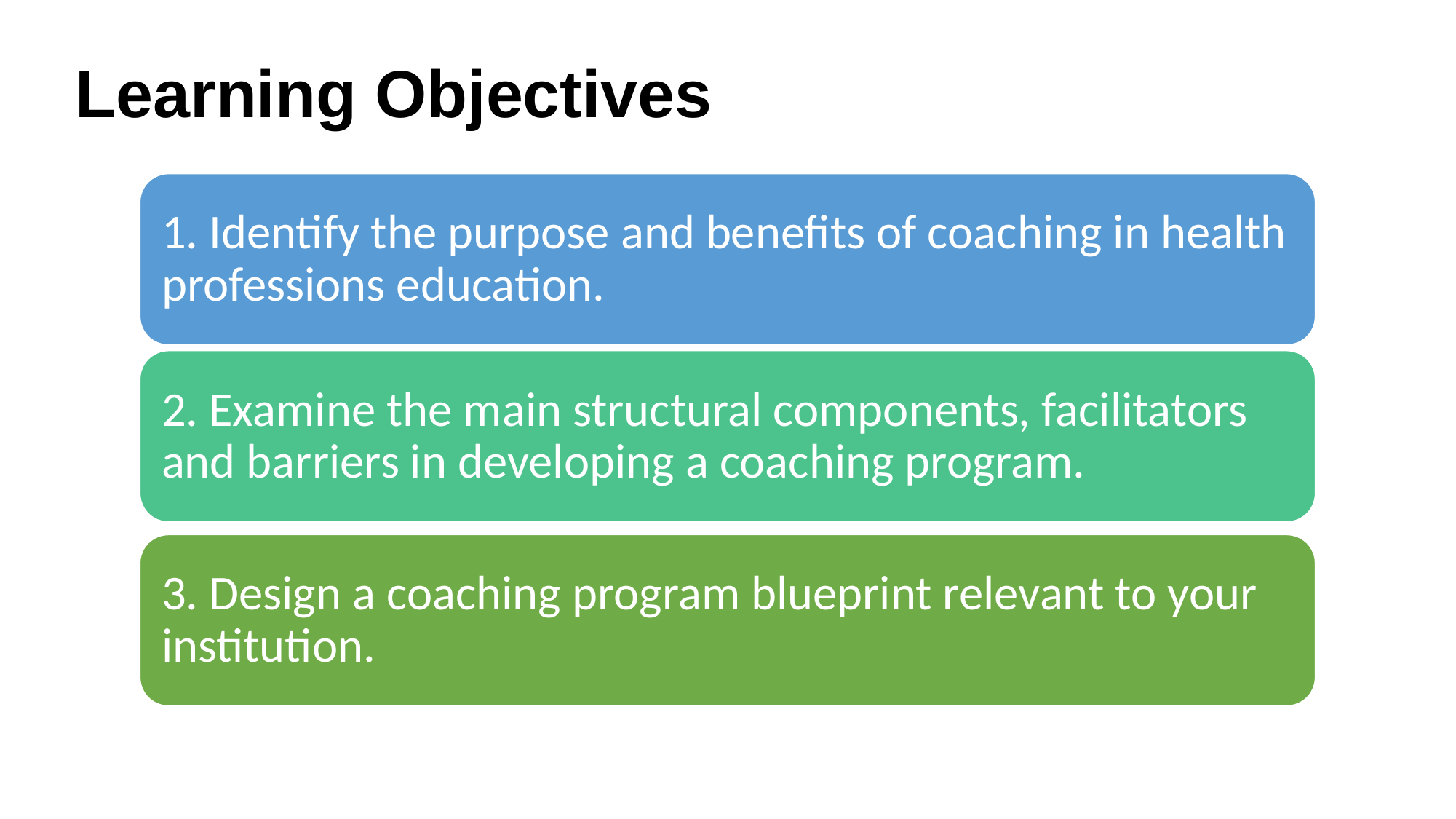

Learning Objectives
1. Identify the purpose and benefits of coaching in health professions education.
2. Examine the main structural components, facilitators and barriers in developing a coaching program.
3. Design a coaching program blueprint relevant to your institution.

## Slide 4
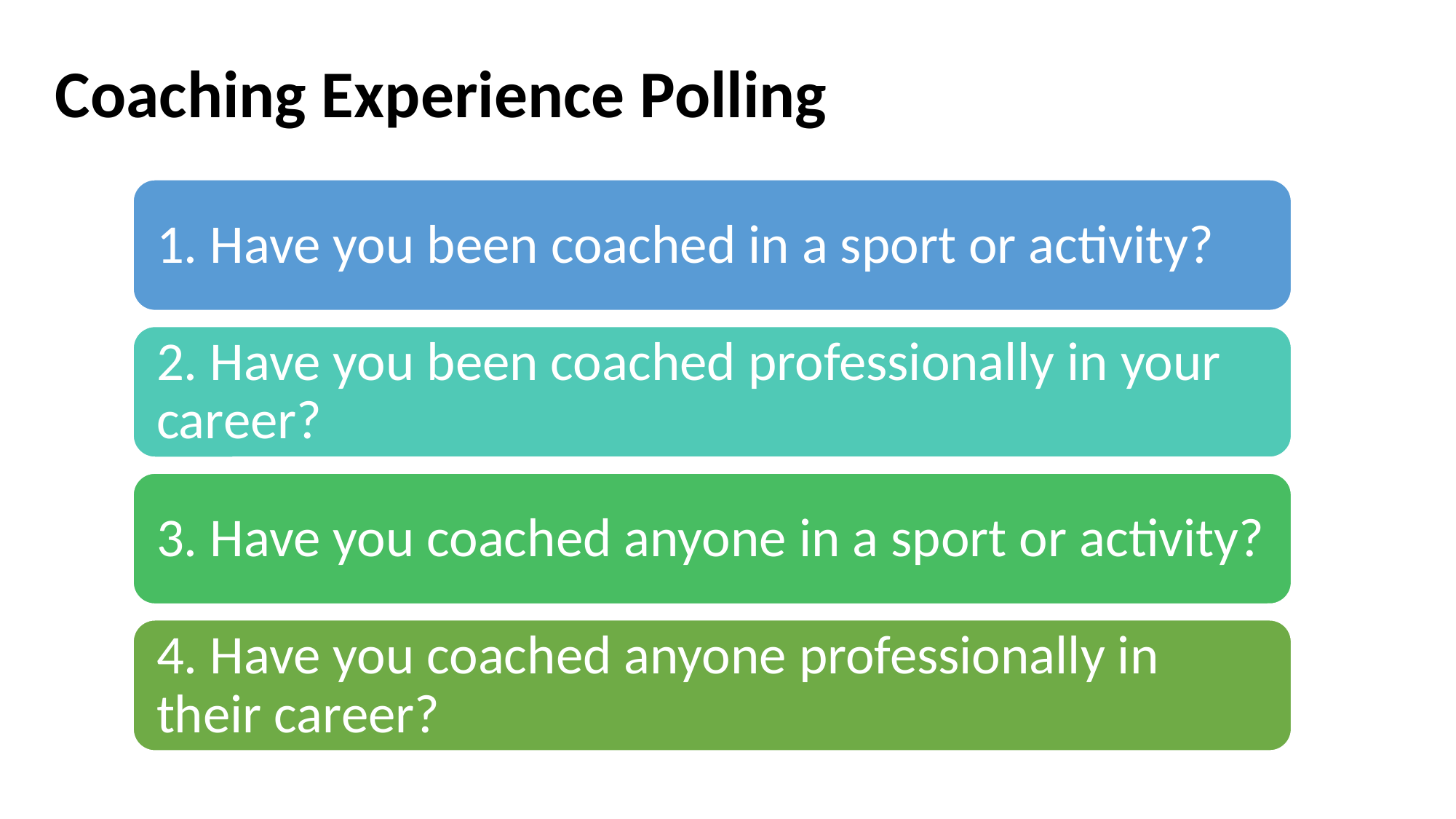

# Coaching Experience Polling
1. Have you been coached in a sport or activity?
2. Have you been coached professionally in your career?
3. Have you coached anyone in a sport or activity?
4. Have you coached anyone professionally in their career?

## Slide 5
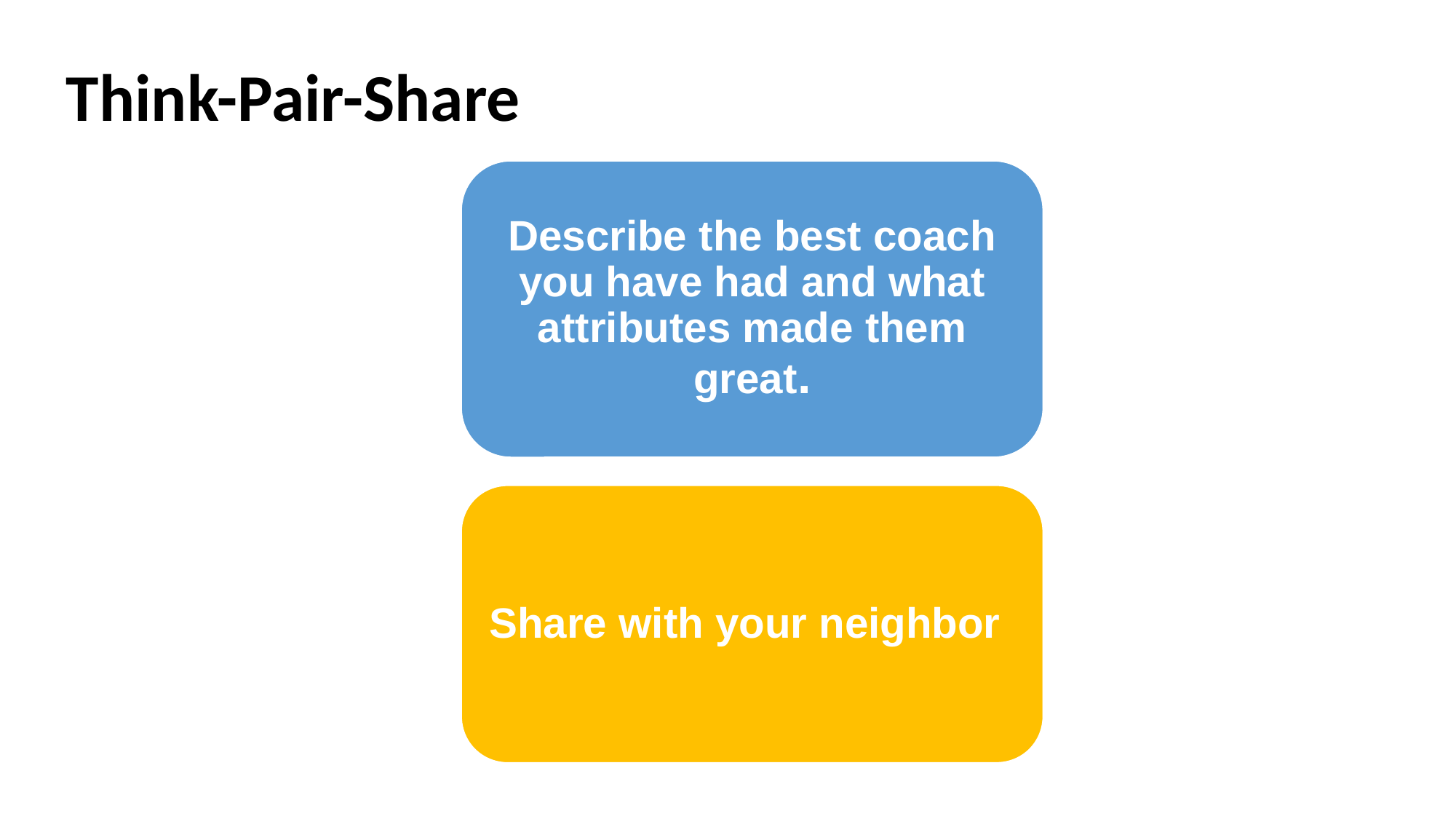

# Think-Pair-Share
Describe the best coach you have had and what attributes made them great.
Share with your neighbor

## Slide 6
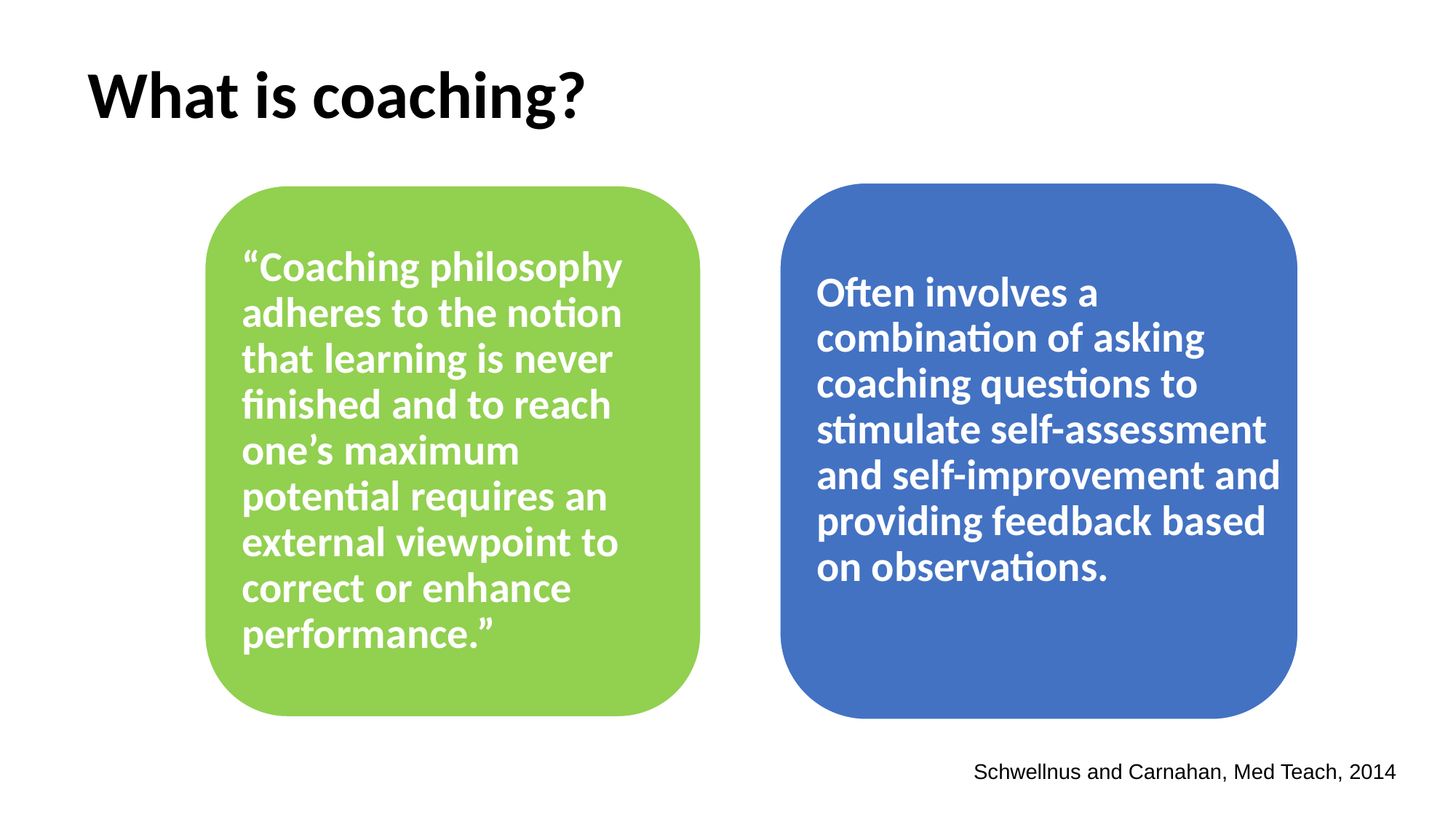

# What is coaching?
Often involves a combination of asking coaching questions to stimulate self-assessment and self-improvement and providing feedback based on observations.
“Coaching philosophy adheres to the notion that learning is never finished and to reach one’s maximum potential requires an external viewpoint to correct or enhance performance.”
Schwellnus and Carnahan, Med Teach, 2014

## Slide 7
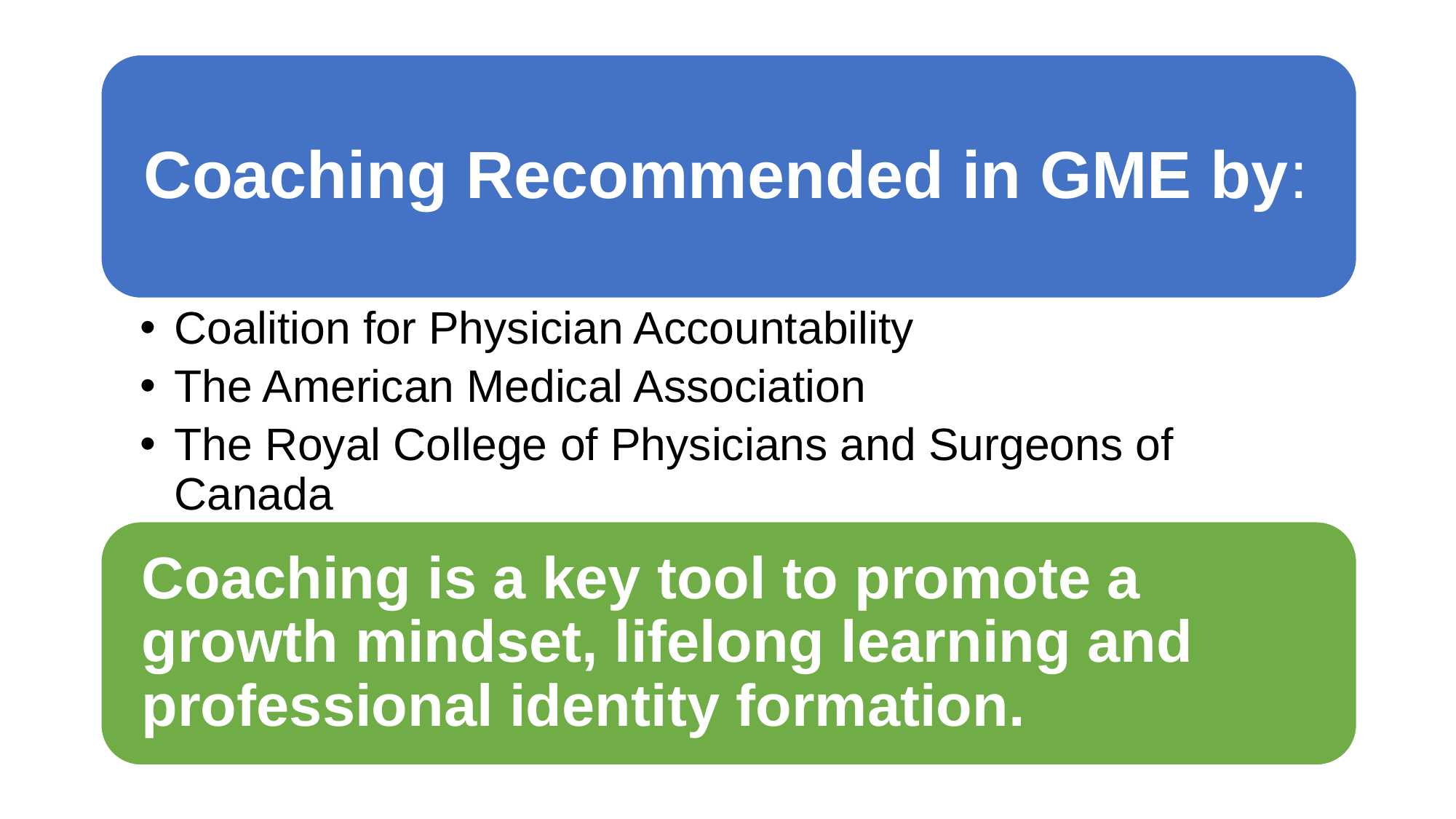

## Slide 8
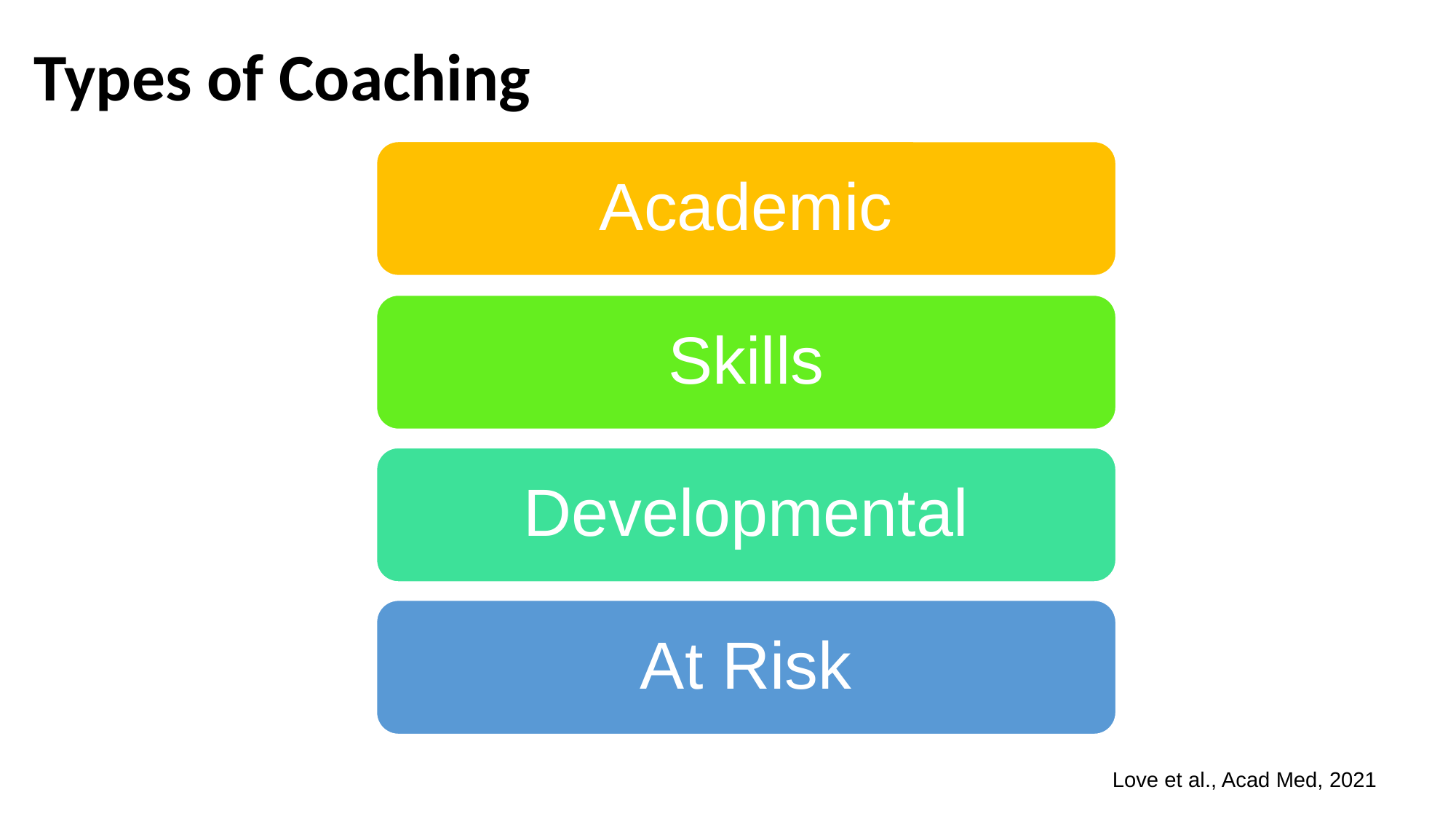

# Types of Coaching
Academic
Skills
Developmental
At Risk
Love et al., Acad Med, 2021

## Slide 9
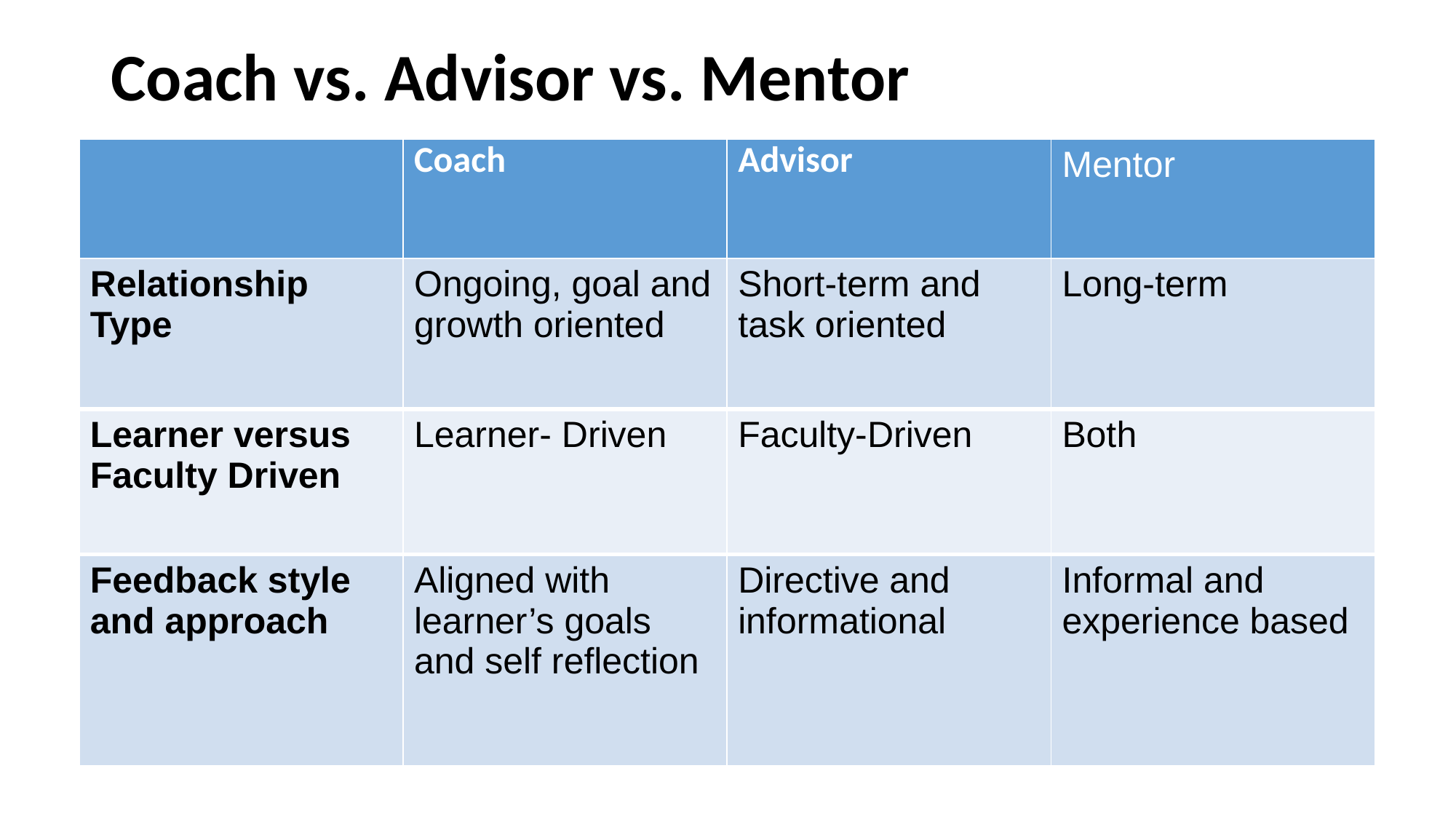

# Coach vs. Advisor vs. Mentor
| | Coach | Advisor | Mentor |
| --- | --- | --- | --- |
| Relationship Type | Ongoing, goal and growth oriented | Short-term and task oriented | Long-term |
| Learner versus Faculty Driven | Learner- Driven | Faculty-Driven | Both |
| Feedback style and approach | Aligned with learner’s goals and self reflection | Directive and informational | Informal and experience based |

## Slide 10
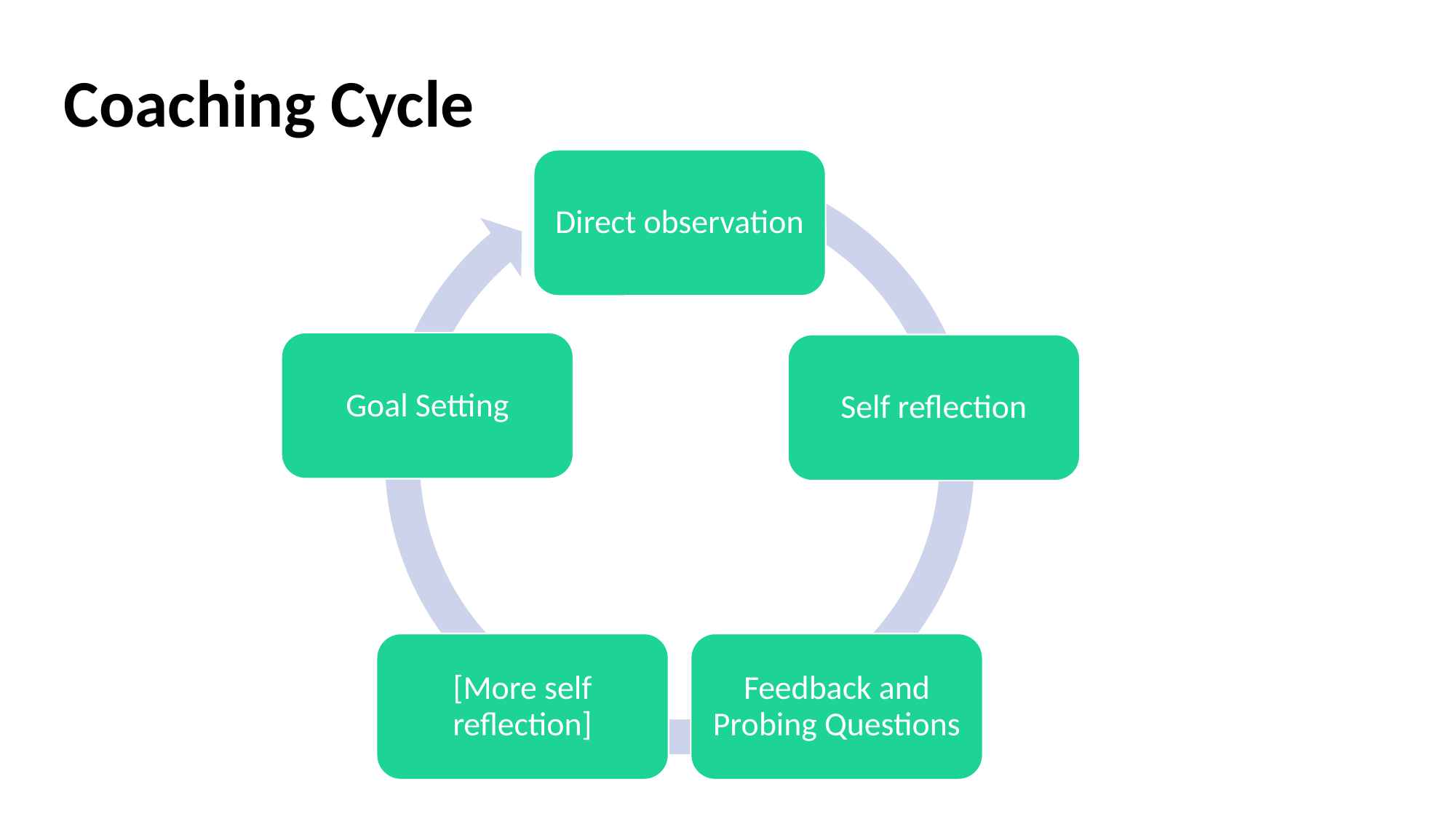

# Coaching Cycle
Direct observation
Goal Setting
Self reflection
[More self reflection]
Feedback and Probing Questions

## Slide 11
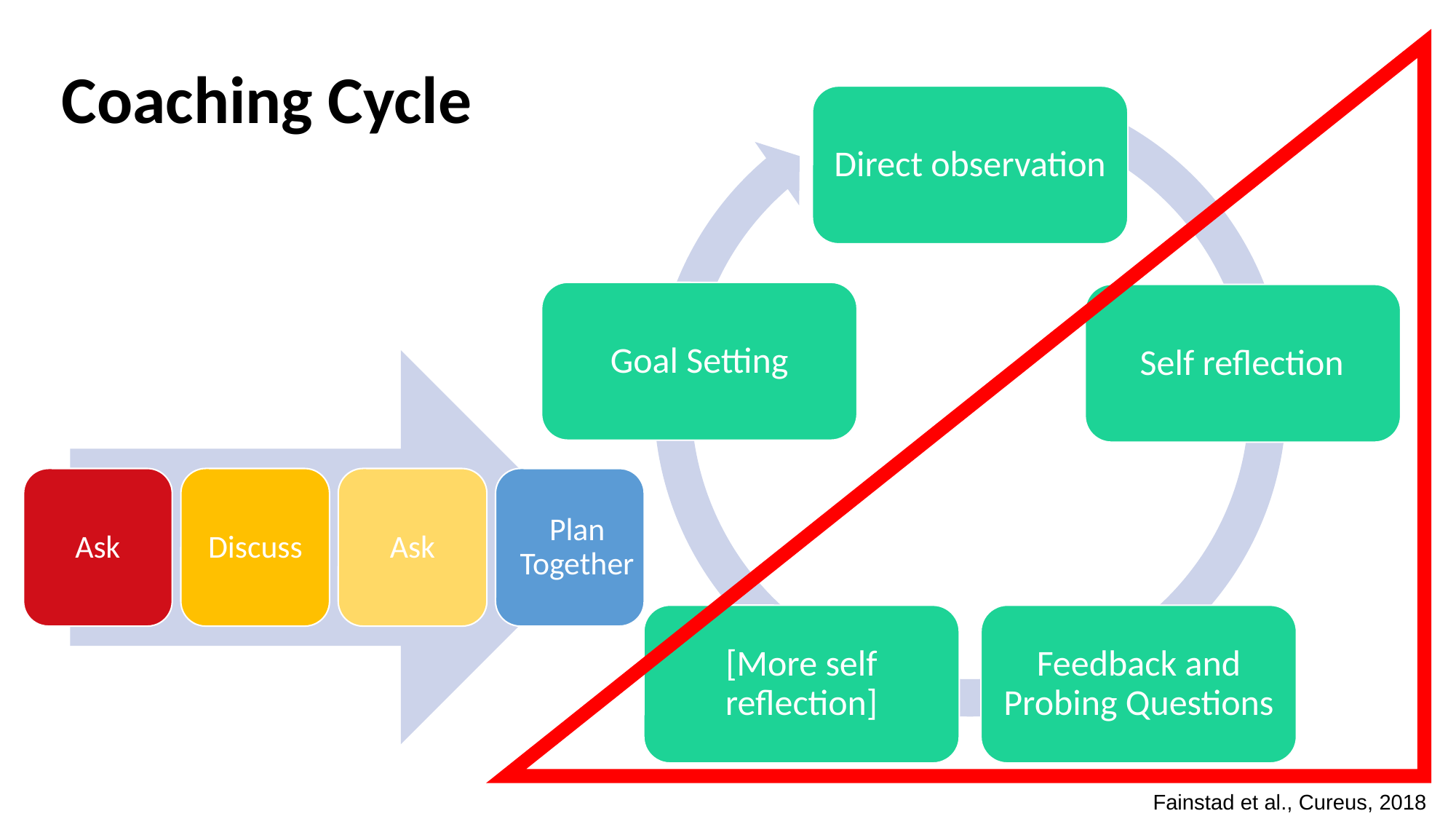

# Coaching Cycle
Direct observation
Goal Setting
Self reflection
[More self reflection]
Feedback and Probing Questions
Ask
Discuss
Ask
Plan Together
Fainstad et al., Cureus, 2018

## Slide 12
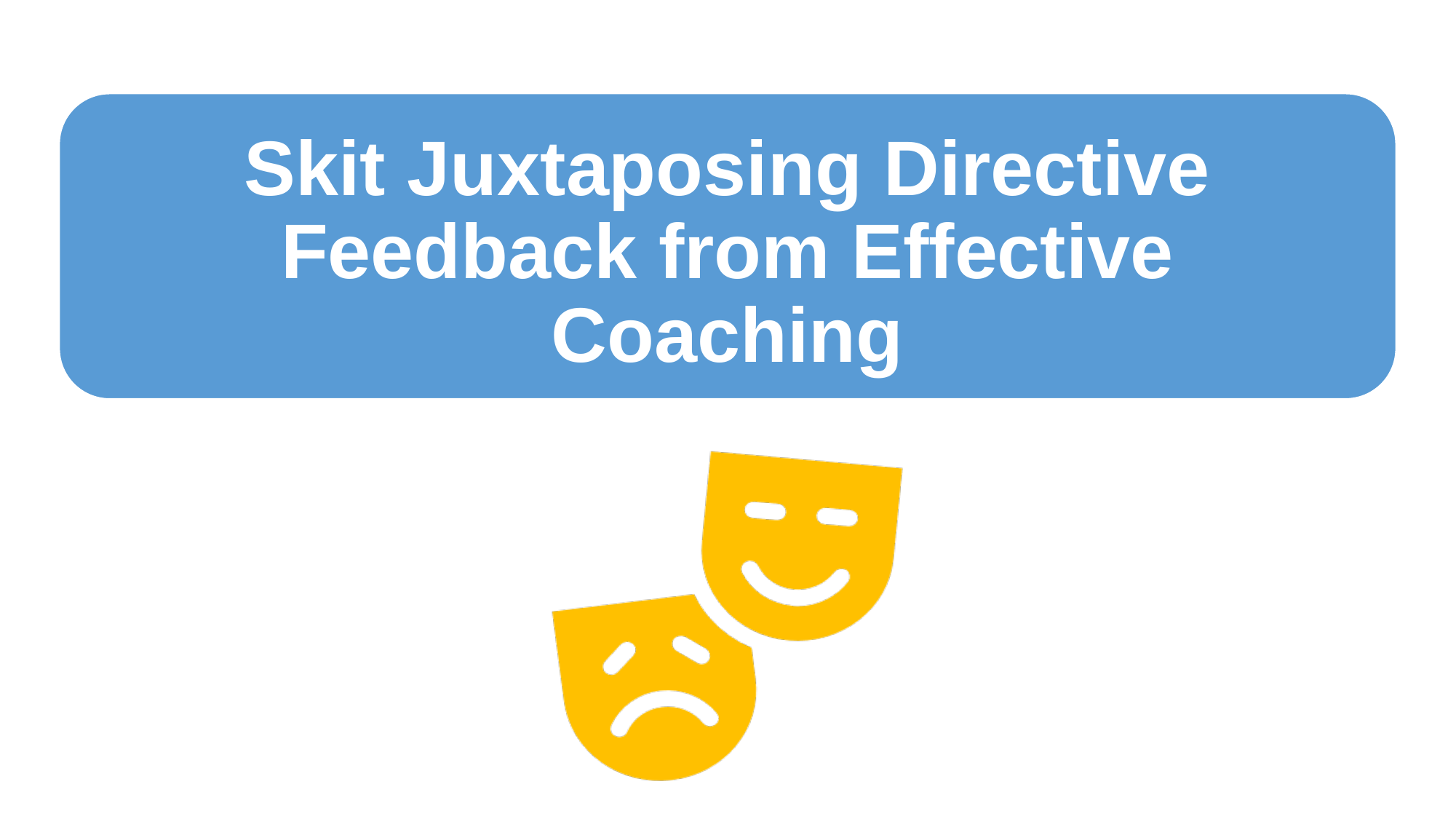

Skit Juxtaposing Directive Feedback from Effective Coaching

## Slide 13
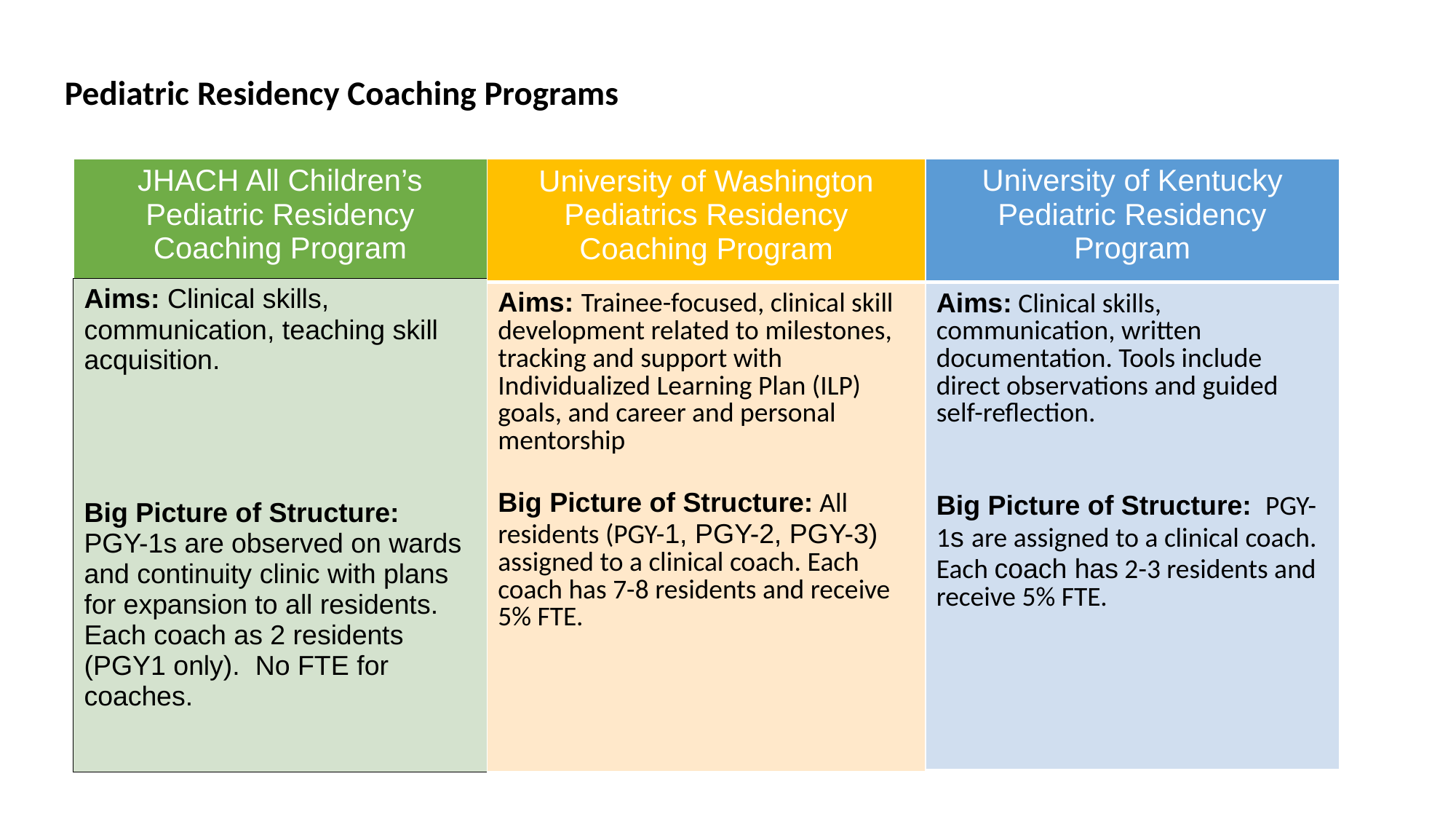

# Pediatric Residency Coaching Programs
| JHACH All Children’s Pediatric Residency Coaching Program |
| --- |
| Aims: Clinical skills, communication, teaching skill acquisition. Big Picture of Structure: PGY-1s are observed on wards and continuity clinic with plans for expansion to all residents. Each coach as 2 residents (PGY1 only). No FTE for coaches. |
| University of Kentucky Pediatric Residency Program |
| --- |
| Aims: Clinical skills, communication, written documentation. Tools include direct observations and guided self-reflection. Big Picture of Structure: PGY-1s are assigned to a clinical coach. Each coach has 2-3 residents and receive 5% FTE. |
| University of Washington Pediatrics Residency Coaching Program |
| --- |
| Aims: Trainee-focused, clinical skill development related to milestones, tracking and support with Individualized Learning Plan (ILP) goals, and career and personal mentorship Big Picture of Structure: All residents (PGY-1, PGY-2, PGY-3) assigned to a clinical coach. Each coach has 7-8 residents and receive 5% FTE. |

## Slide 14
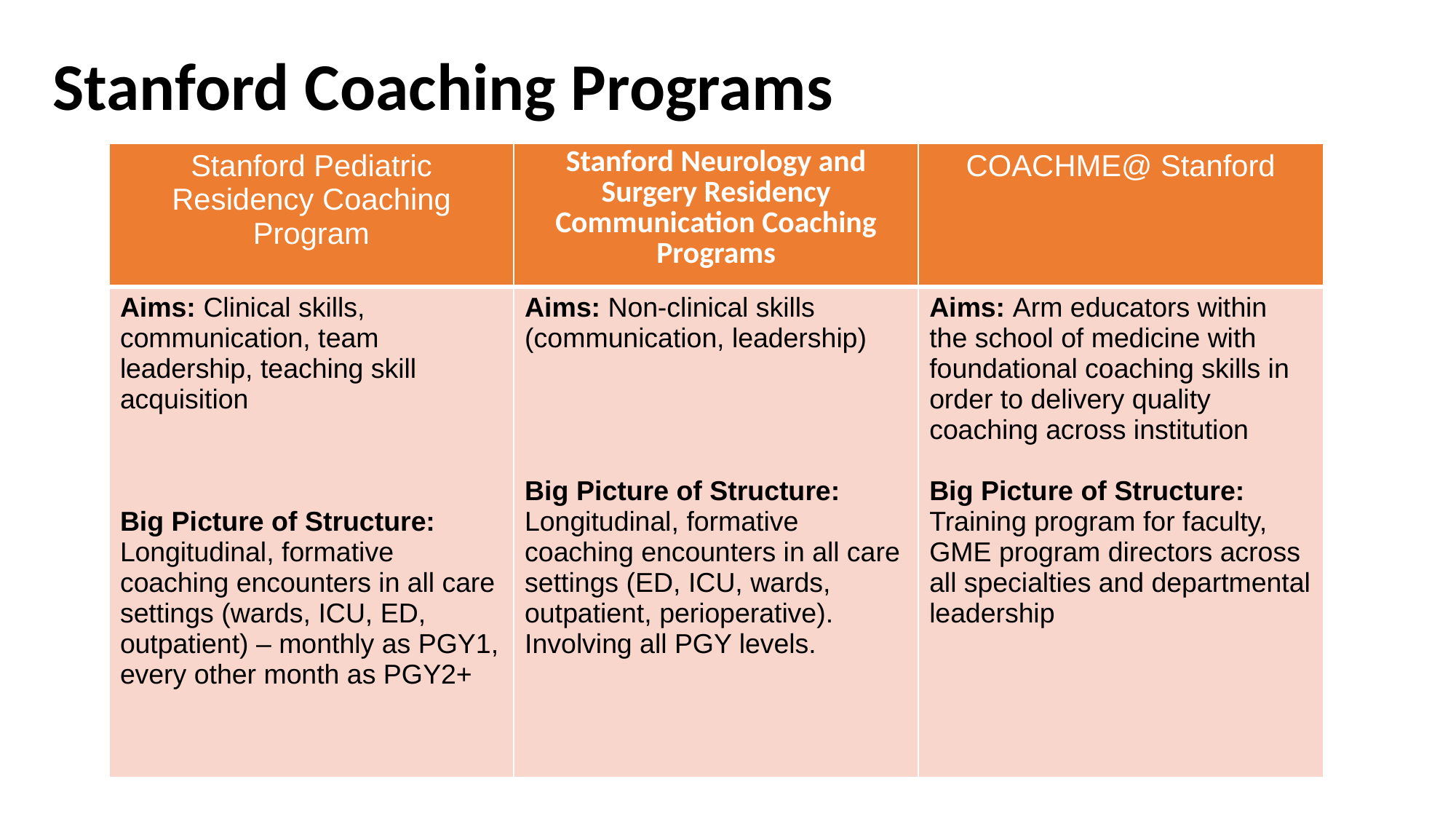

# Stanford Coaching Programs
| Stanford Pediatric Residency Coaching Program | Stanford Neurology and Surgery Residency Communication Coaching Programs | COACHME@ Stanford |
| --- | --- | --- |
| Aims: Clinical skills, communication, team leadership, teaching skill acquisition Big Picture of Structure: Longitudinal, formative coaching encounters in all care settings (wards, ICU, ED, outpatient) – monthly as PGY1, every other month as PGY2+ | Aims: Non-clinical skills (communication, leadership) Big Picture of Structure: Longitudinal, formative coaching encounters in all care settings (ED, ICU, wards, outpatient, perioperative). Involving all PGY levels. | Aims: Arm educators within the school of medicine with foundational coaching skills in order to delivery quality coaching across institution Big Picture of Structure: Training program for faculty, GME program directors across all specialties and departmental leadership |

## Slide 15
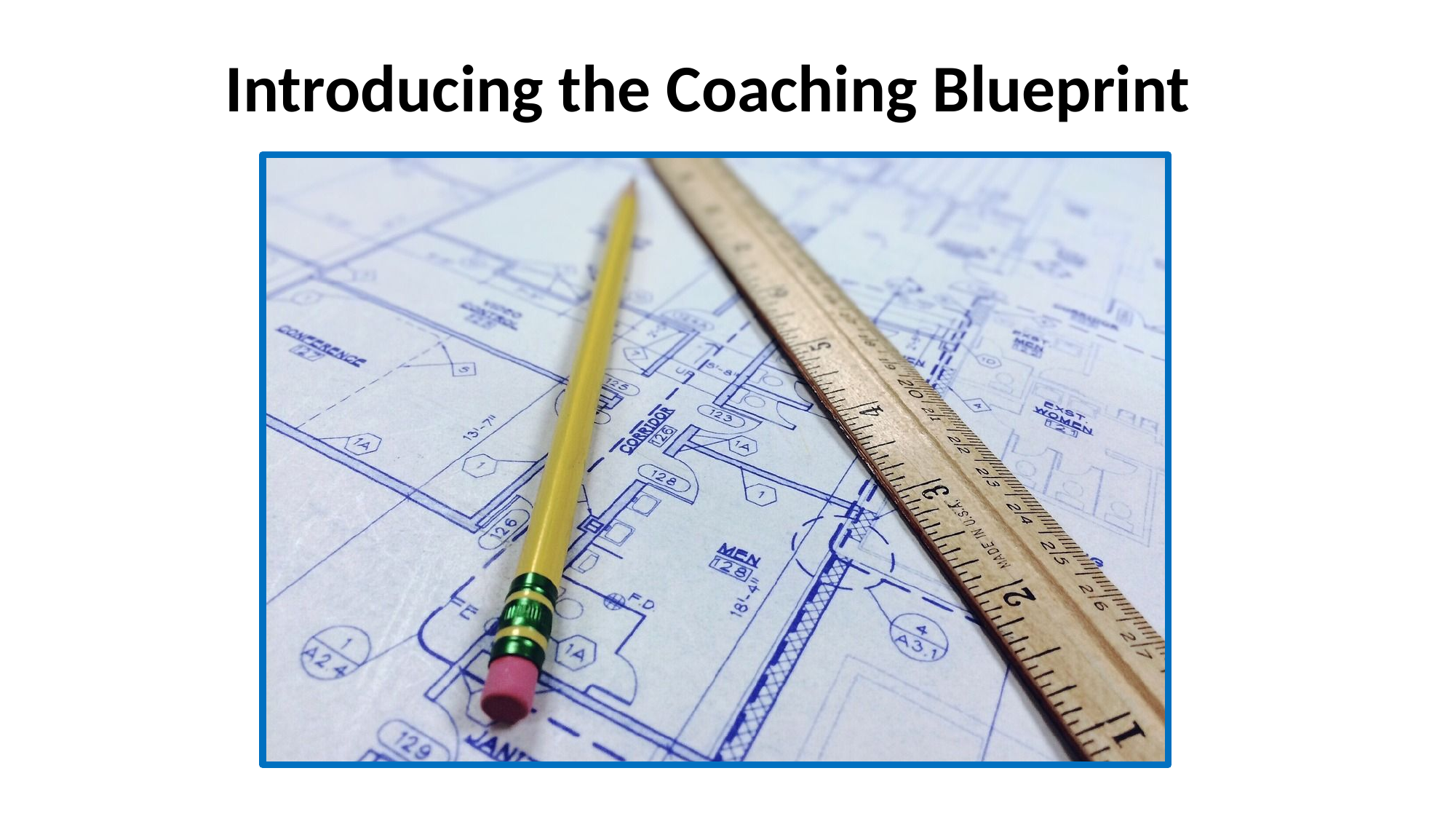

# Introducing the Coaching Blueprint

## Slide 16
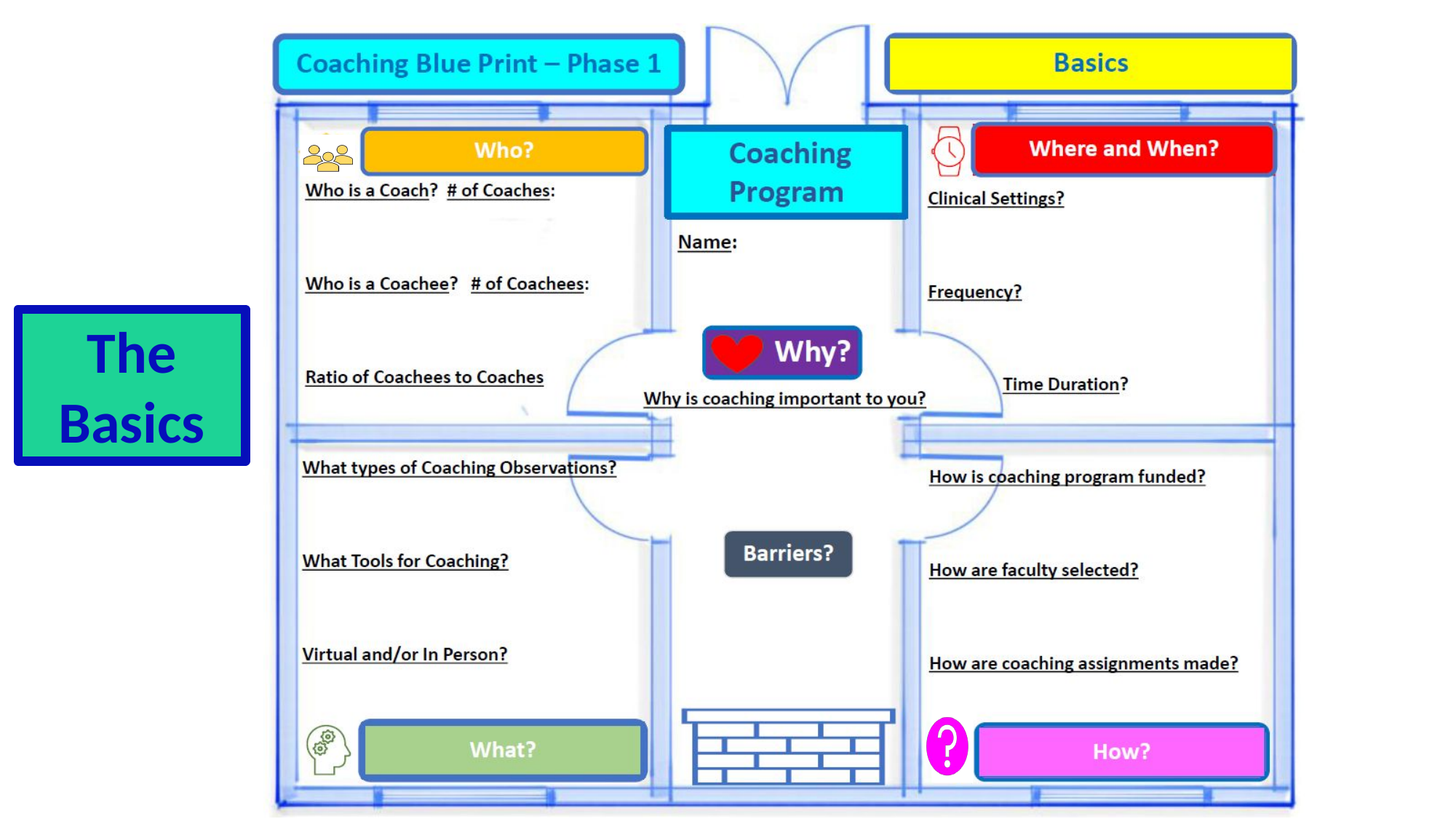

Coaching Blueprint Phase 1
The Basics

## Slide 17
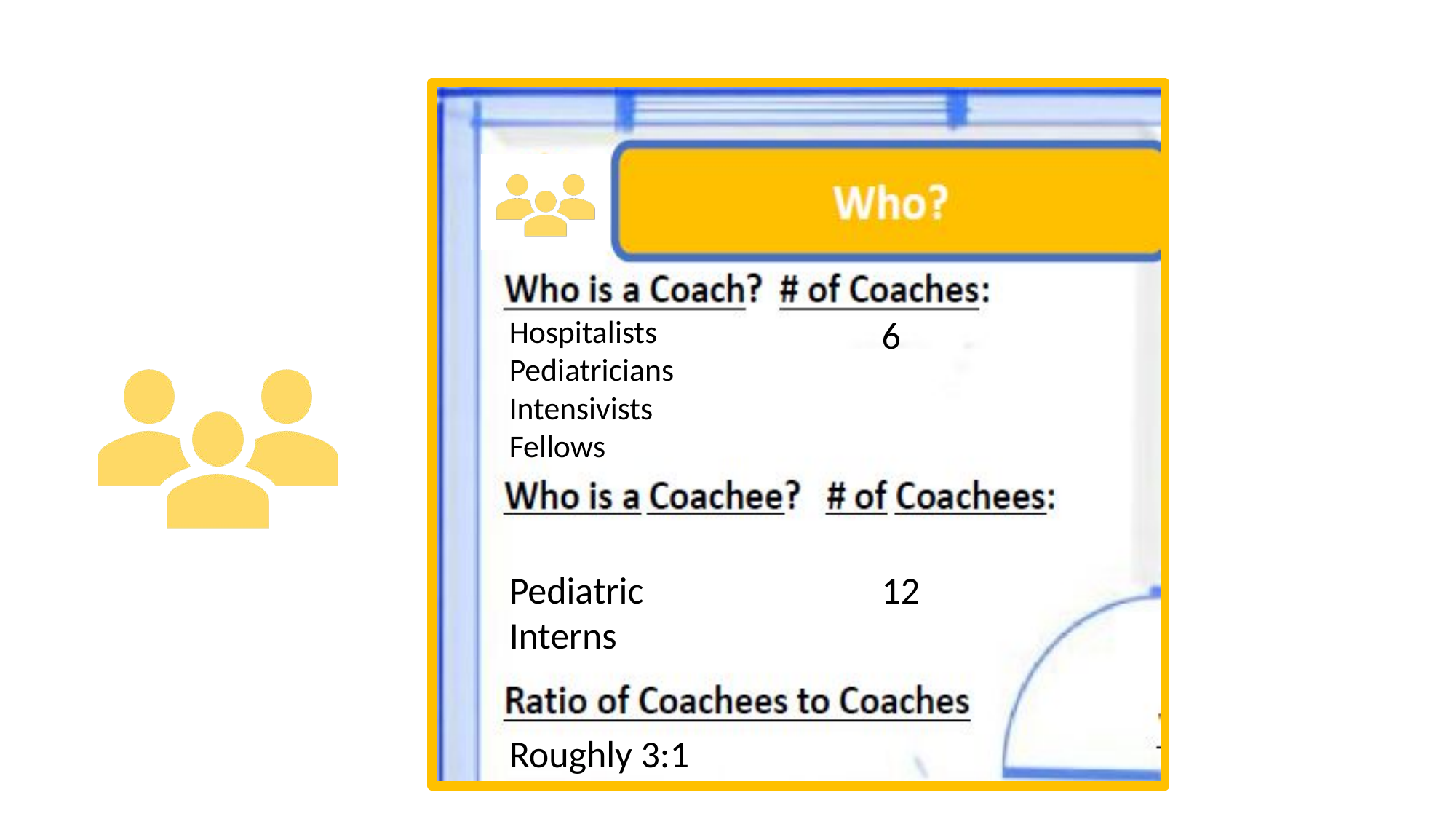

# Who?
6
Hospitalists
Pediatricians
Intensivists
Fellows
Pediatric Interns
12
Roughly 3:1

## Slide 18
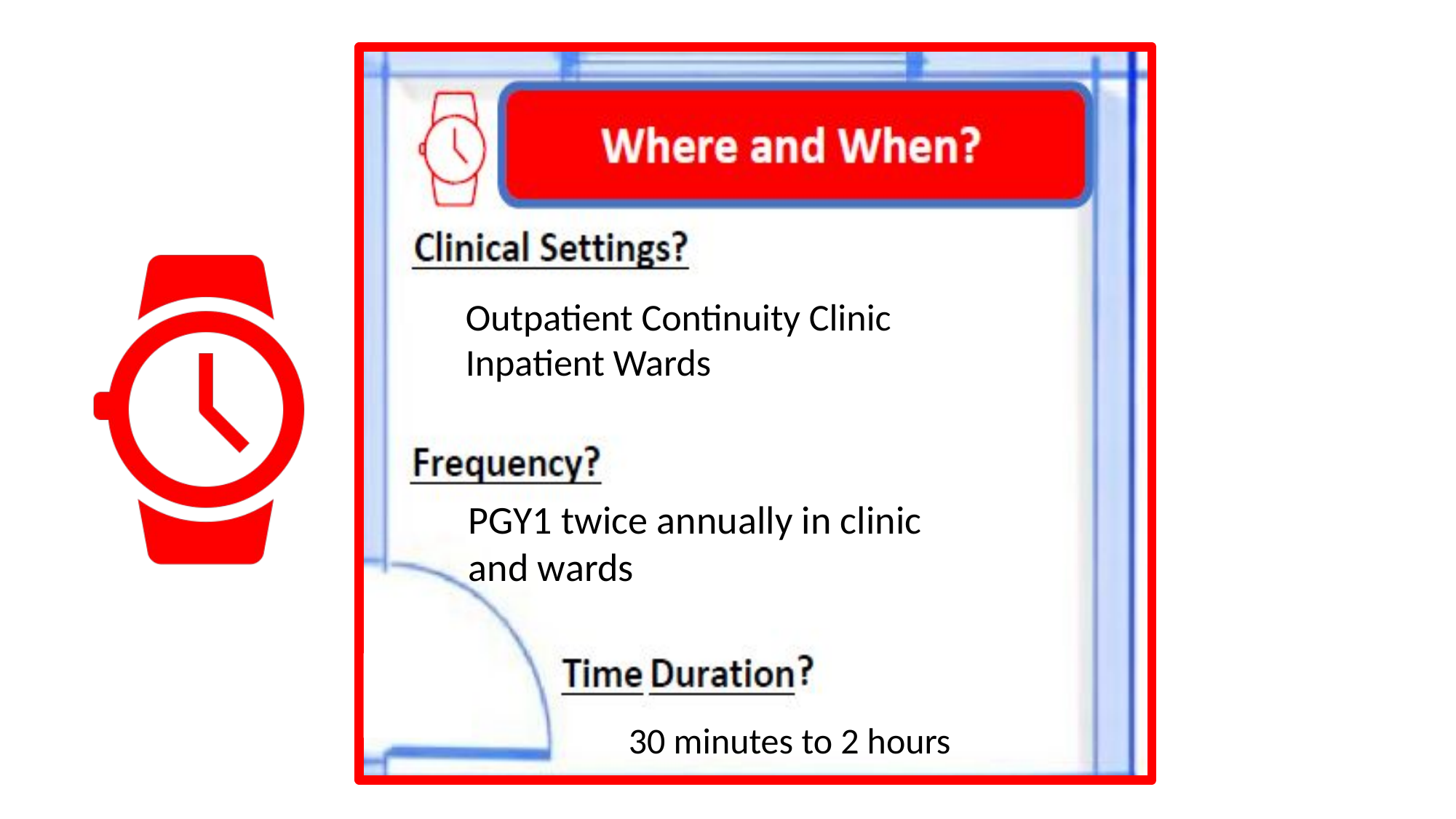

# Where and When?
Outpatient Continuity Clinic
Inpatient Wards
PGY1 twice annually in clinic and wards
30 minutes to 2 hours

## Slide 19
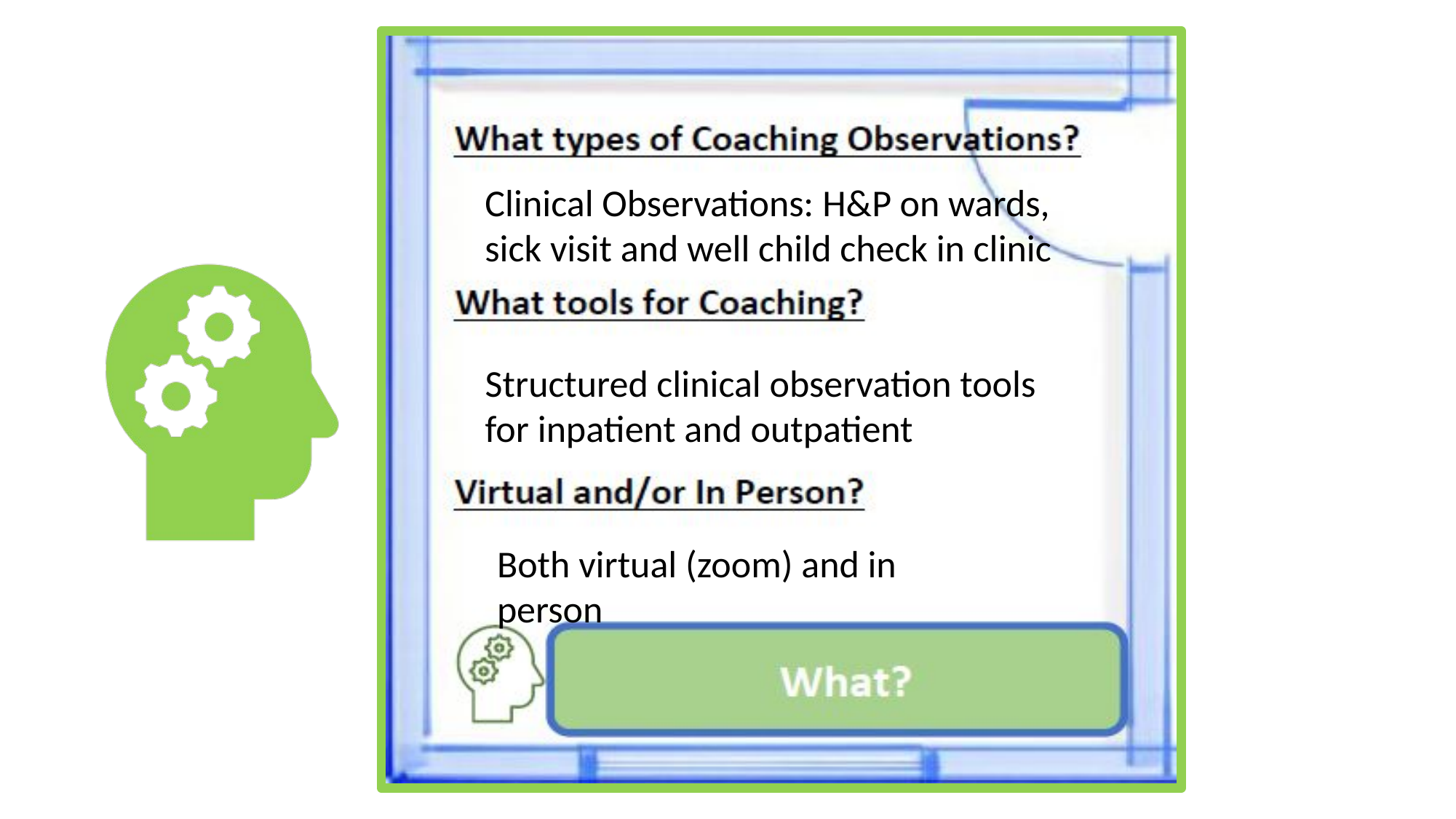

# What?
Clinical Observations: H&P on wards, sick visit and well child check in clinic
Structured clinical observation tools for inpatient and outpatient
Both virtual (zoom) and in person

## Slide 20
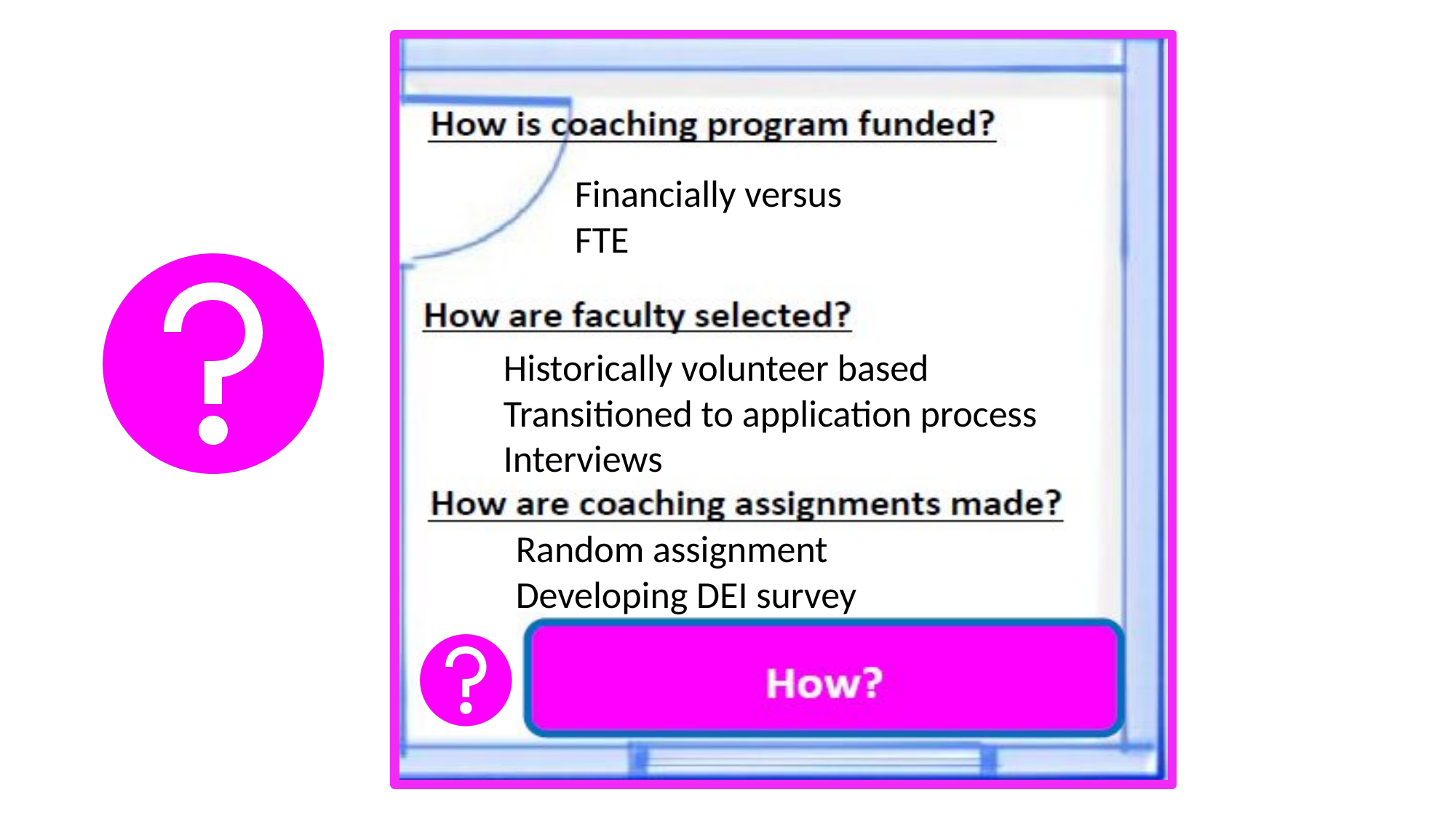

# How?
Financially versus FTE
Historically volunteer based
Transitioned to application process
Interviews
Random assignment
Developing DEI survey

## Slide 21
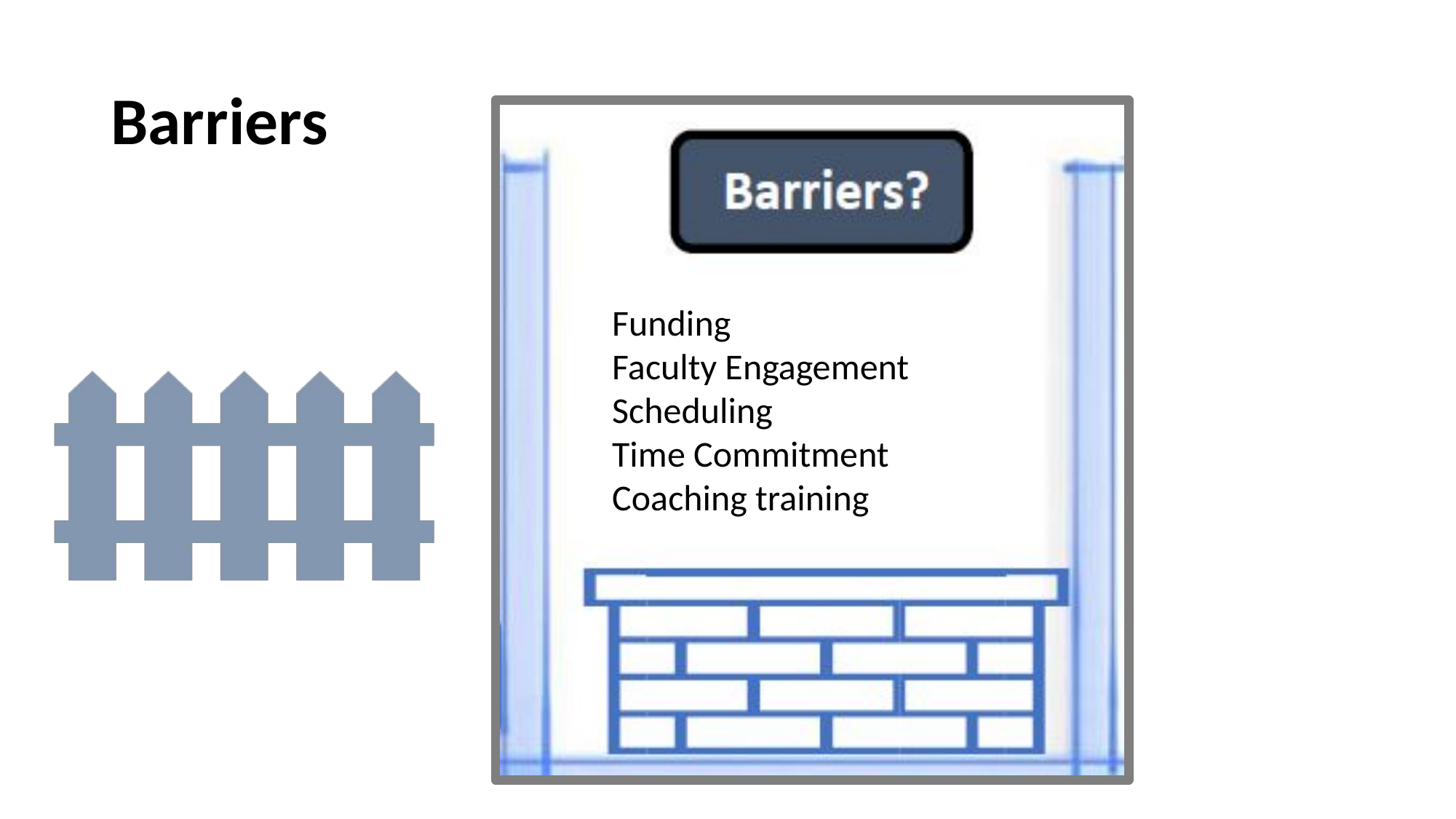

# Barriers
Funding
Faculty Engagement
Scheduling
Time Commitment
Coaching training

## Slide 22
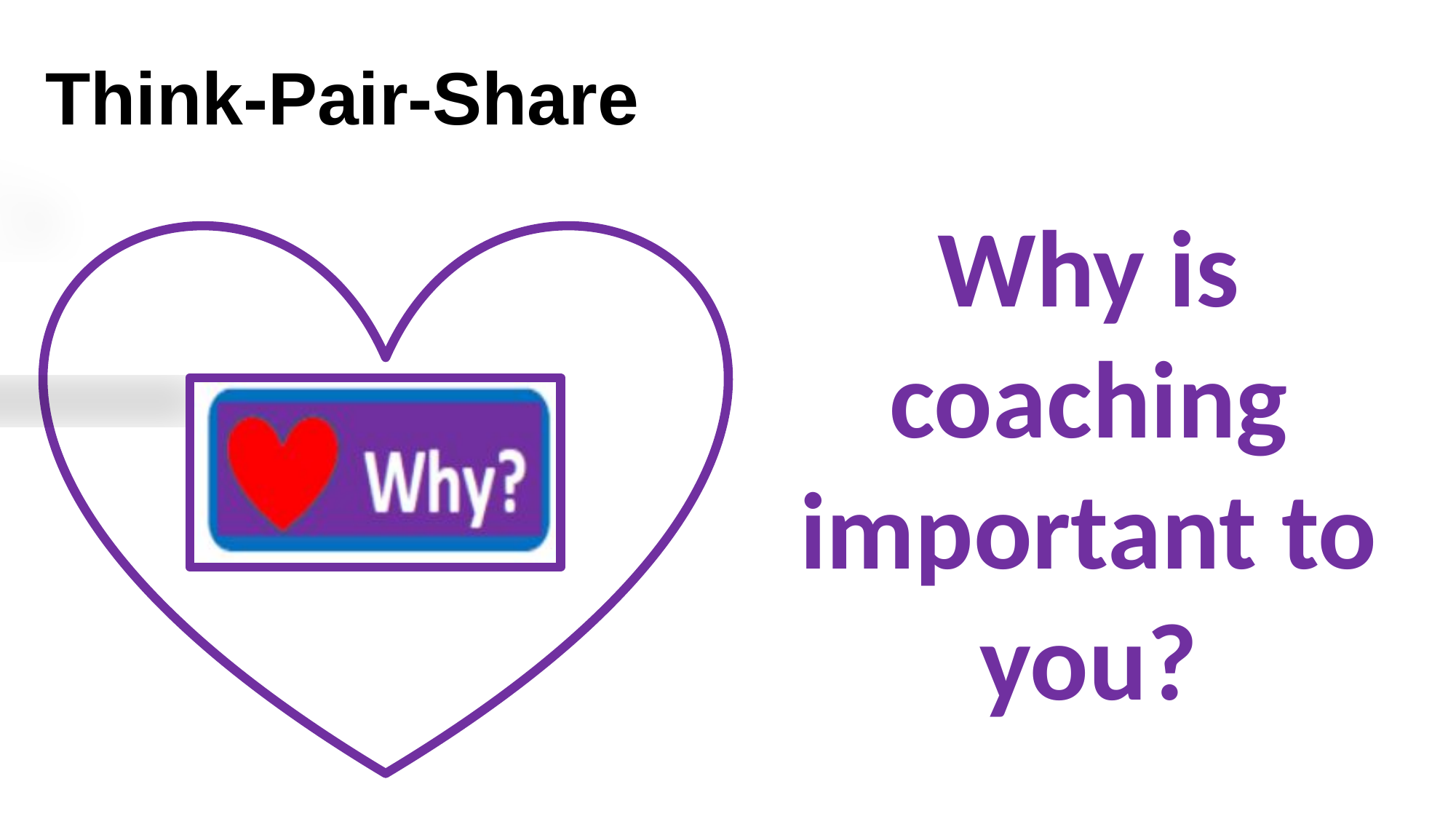

# Think-Pair-Share
Why is coaching important to you?

## Slide 23
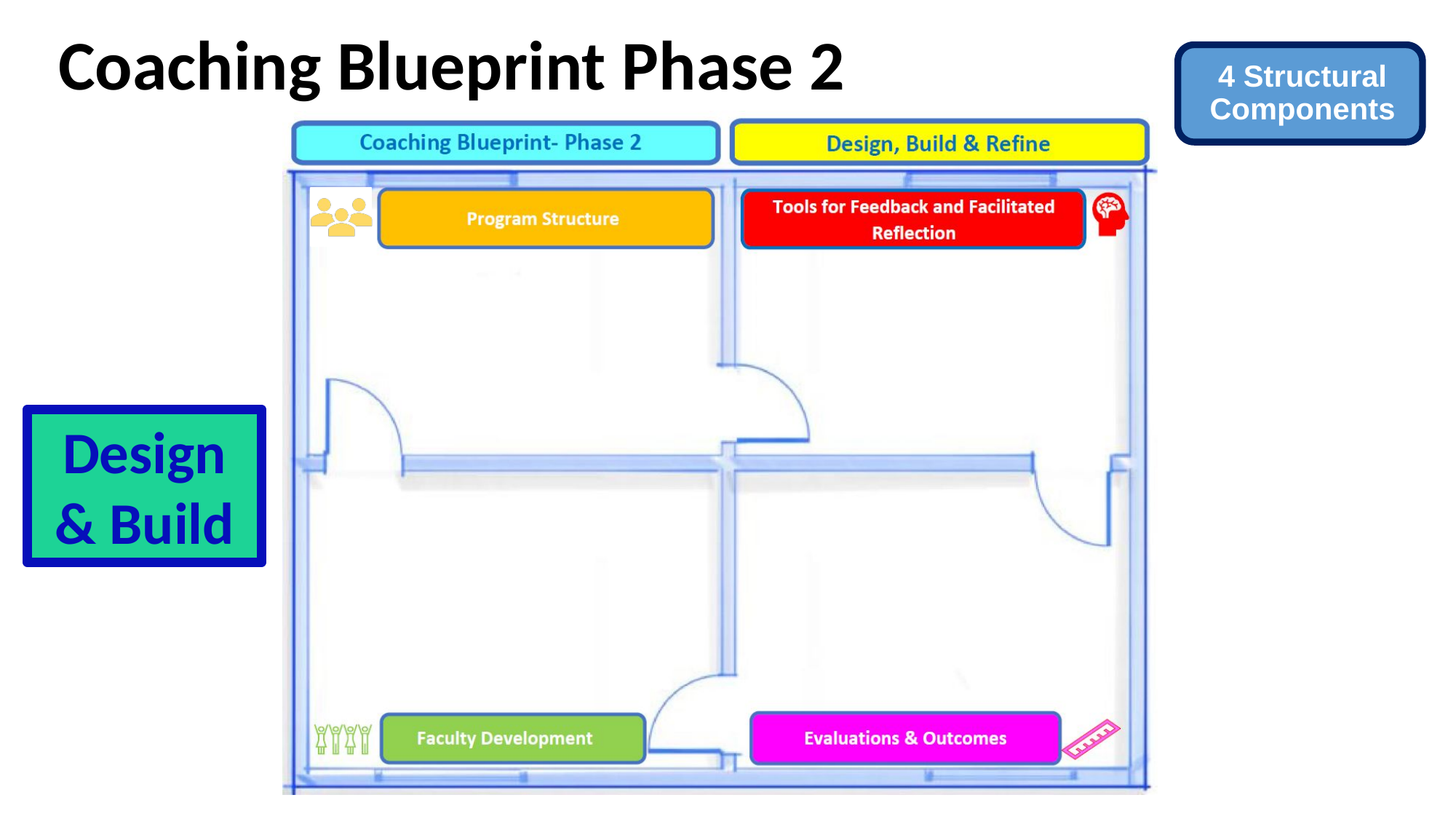

# Coaching Blueprint Phase 2
Design & Build

## Slide 24
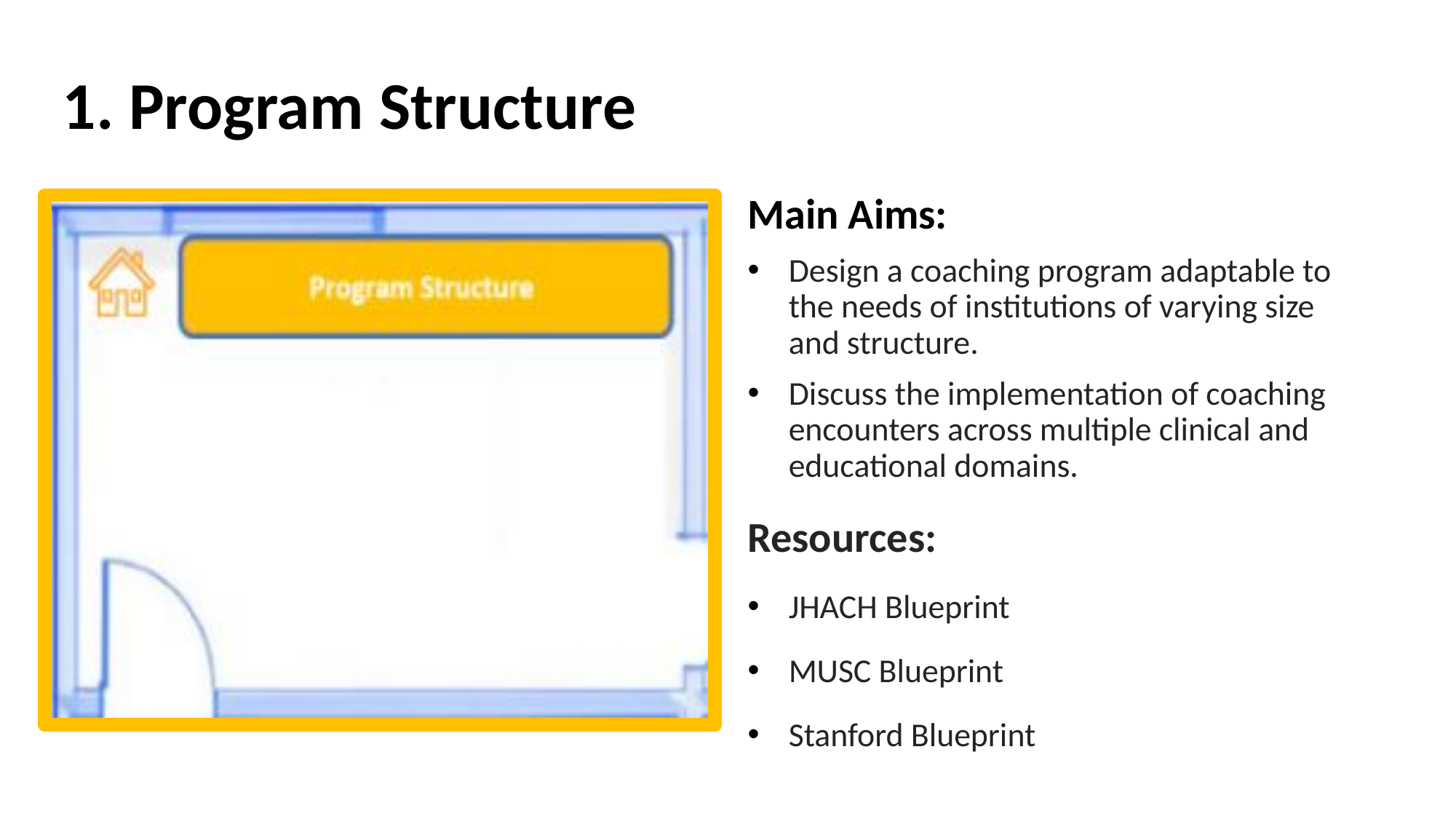

# 1. Program Structure
Main Aims:
Design a coaching program adaptable to the needs of institutions of varying size and structure.
Discuss the implementation of coaching encounters across multiple clinical and educational domains.
Resources:
JHACH Blueprint
MUSC Blueprint
Stanford Blueprint

## Slide 25
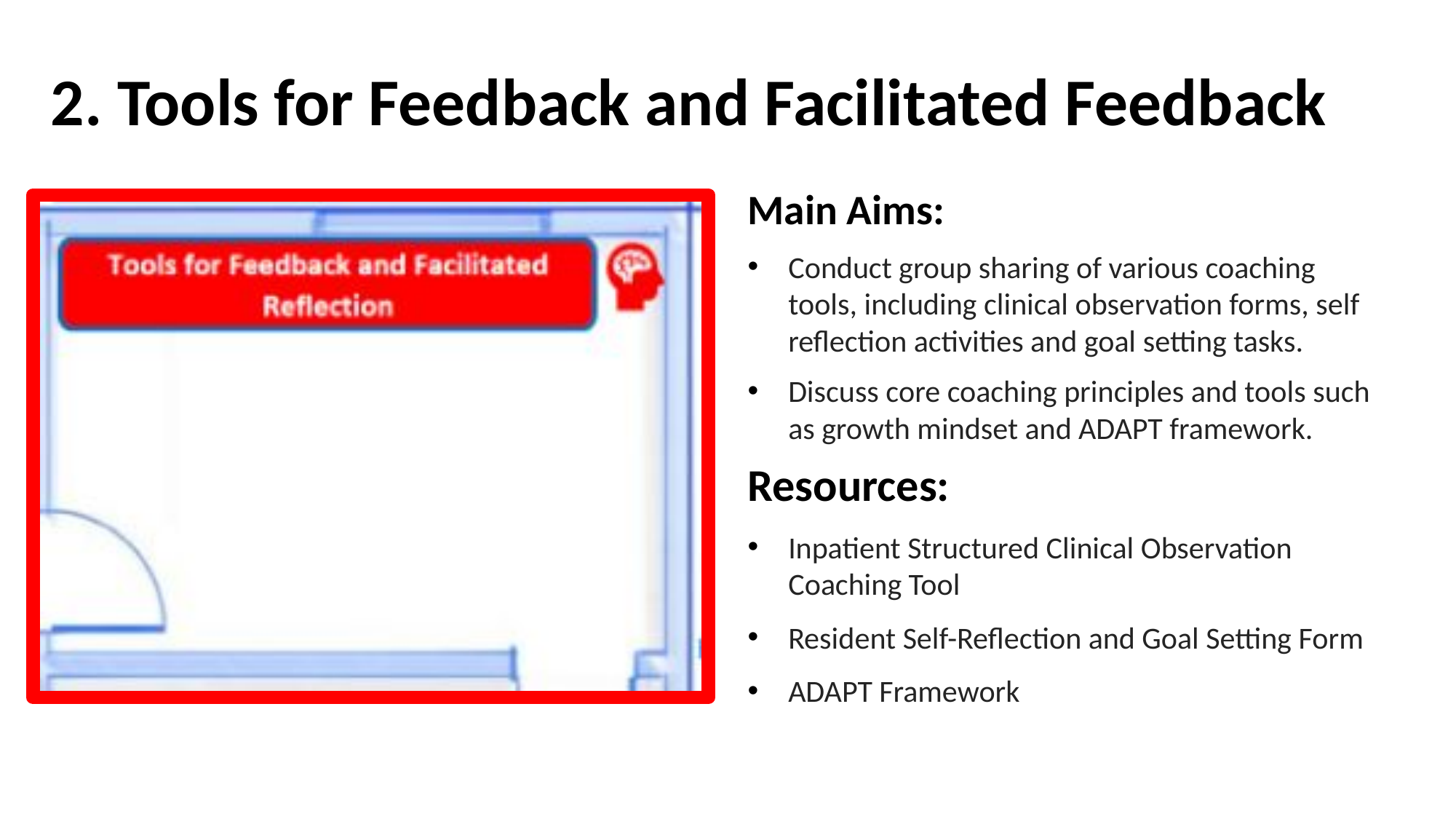

# 2. Tools for Feedback and Facilitated Feedback
Main Aims:
Conduct group sharing of various coaching tools, including clinical observation forms, self reflection activities and goal setting tasks.
Discuss core coaching principles and tools such as growth mindset and ADAPT framework.
Resources:
Inpatient Structured Clinical Observation Coaching Tool
Resident Self-Reflection and Goal Setting Form
ADAPT Framework

## Slide 26
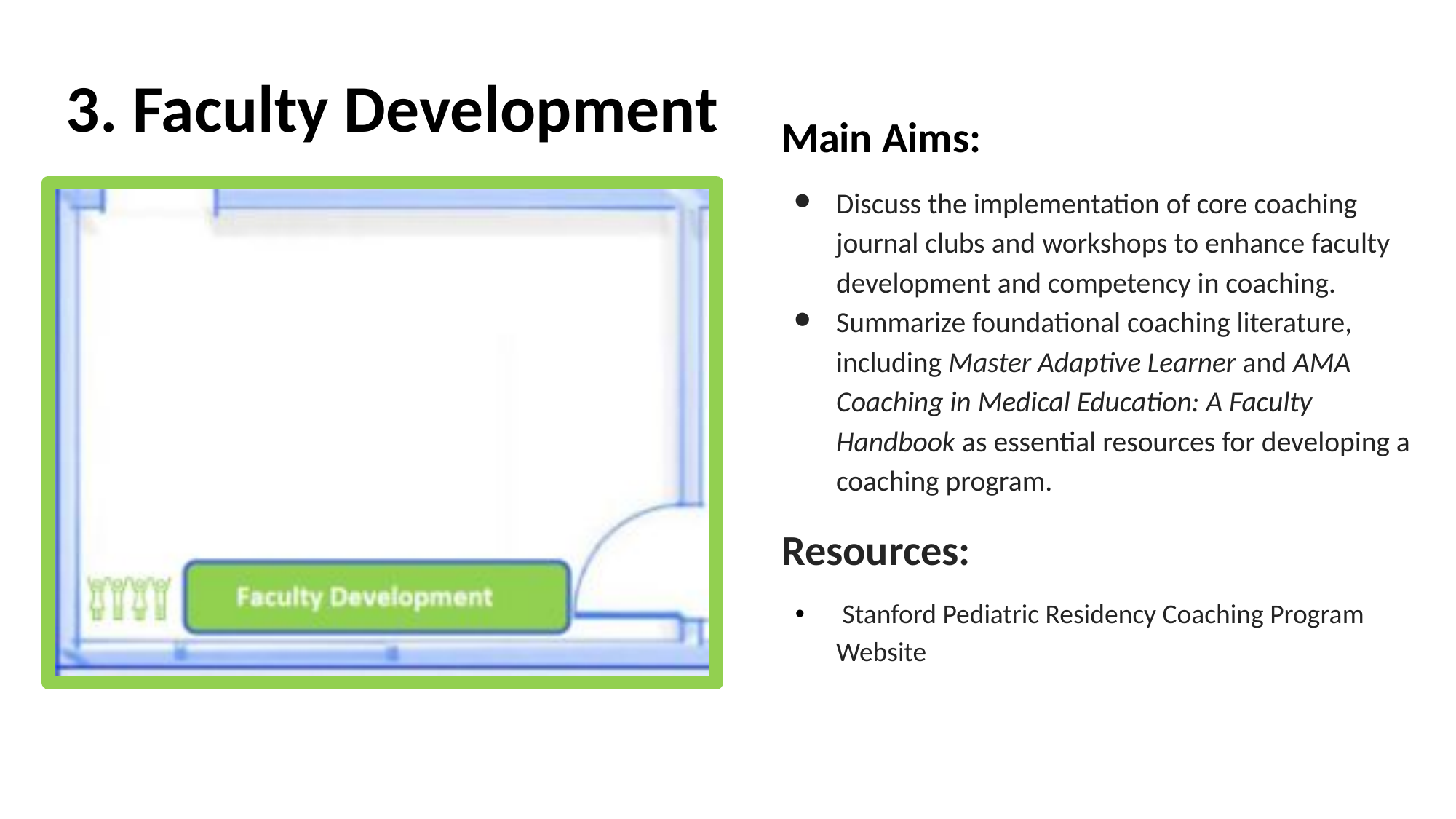

# 3. Faculty Development
Main Aims:
Discuss the implementation of core coaching journal clubs and workshops to enhance faculty development and competency in coaching.
Summarize foundational coaching literature, including Master Adaptive Learner and AMA Coaching in Medical Education: A Faculty Handbook as essential resources for developing a coaching program.
Resources:
 Stanford Pediatric Residency Coaching Program Website

## Slide 27
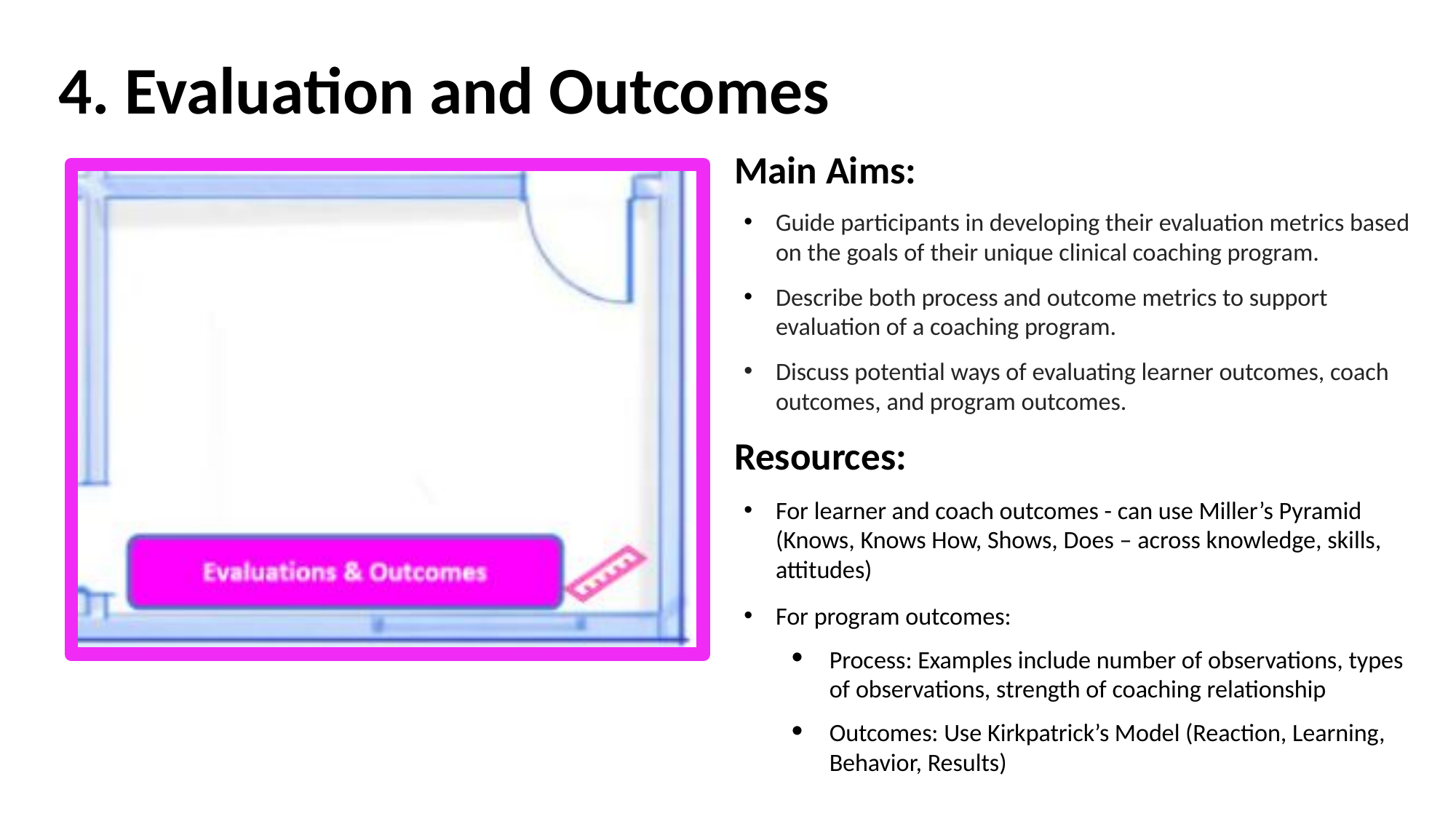

# 4. Evaluation and Outcomes
Main Aims:
Guide participants in developing their evaluation metrics based on the goals of their unique clinical coaching program.
Describe both process and outcome metrics to support evaluation of a coaching program.
Discuss potential ways of evaluating learner outcomes, coach outcomes, and program outcomes.
Resources:
For learner and coach outcomes - can use Miller’s Pyramid (Knows, Knows How, Shows, Does – across knowledge, skills, attitudes)
For program outcomes:
Process: Examples include number of observations, types of observations, strength of coaching relationship
Outcomes: Use Kirkpatrick’s Model (Reaction, Learning, Behavior, Results)

## Slide 28
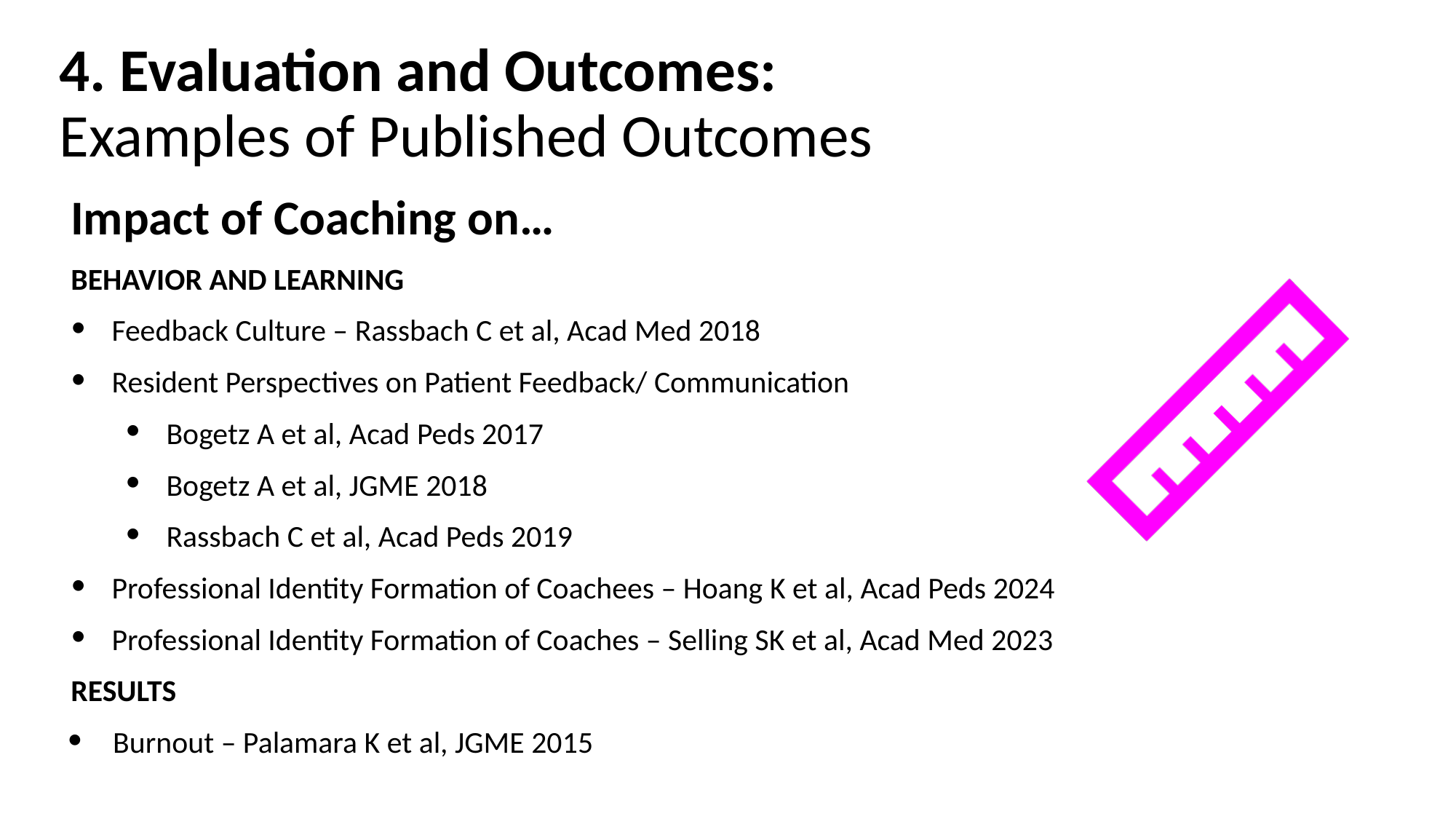

4. Evaluation and Outcomes:
Examples of Published Outcomes
Impact of Coaching on…
BEHAVIOR AND LEARNING
Feedback Culture – Rassbach C et al, Acad Med 2018
Resident Perspectives on Patient Feedback/ Communication
Bogetz A et al, Acad Peds 2017
Bogetz A et al, JGME 2018
Rassbach C et al, Acad Peds 2019
Professional Identity Formation of Coachees – Hoang K et al, Acad Peds 2024
Professional Identity Formation of Coaches – Selling SK et al, Acad Med 2023
RESULTS
Burnout – Palamara K et al, JGME 2015

## Slide 29
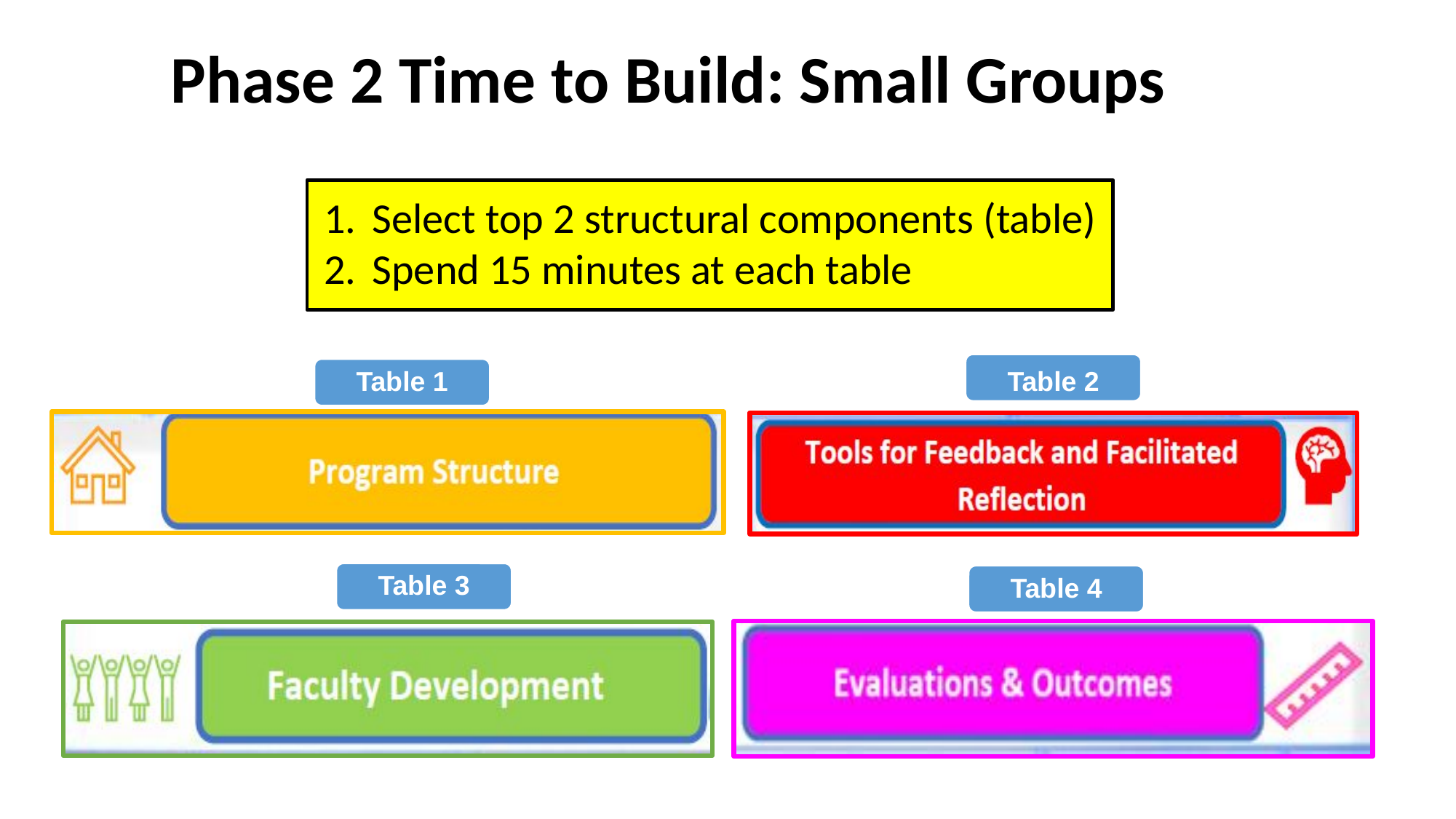

# Phase 2 Time to Build: Small Groups
Select top 2 structural components (table)
Spend 15 minutes at each table
Table 2
Table 1
Table 3
Table 4

## Slide 30
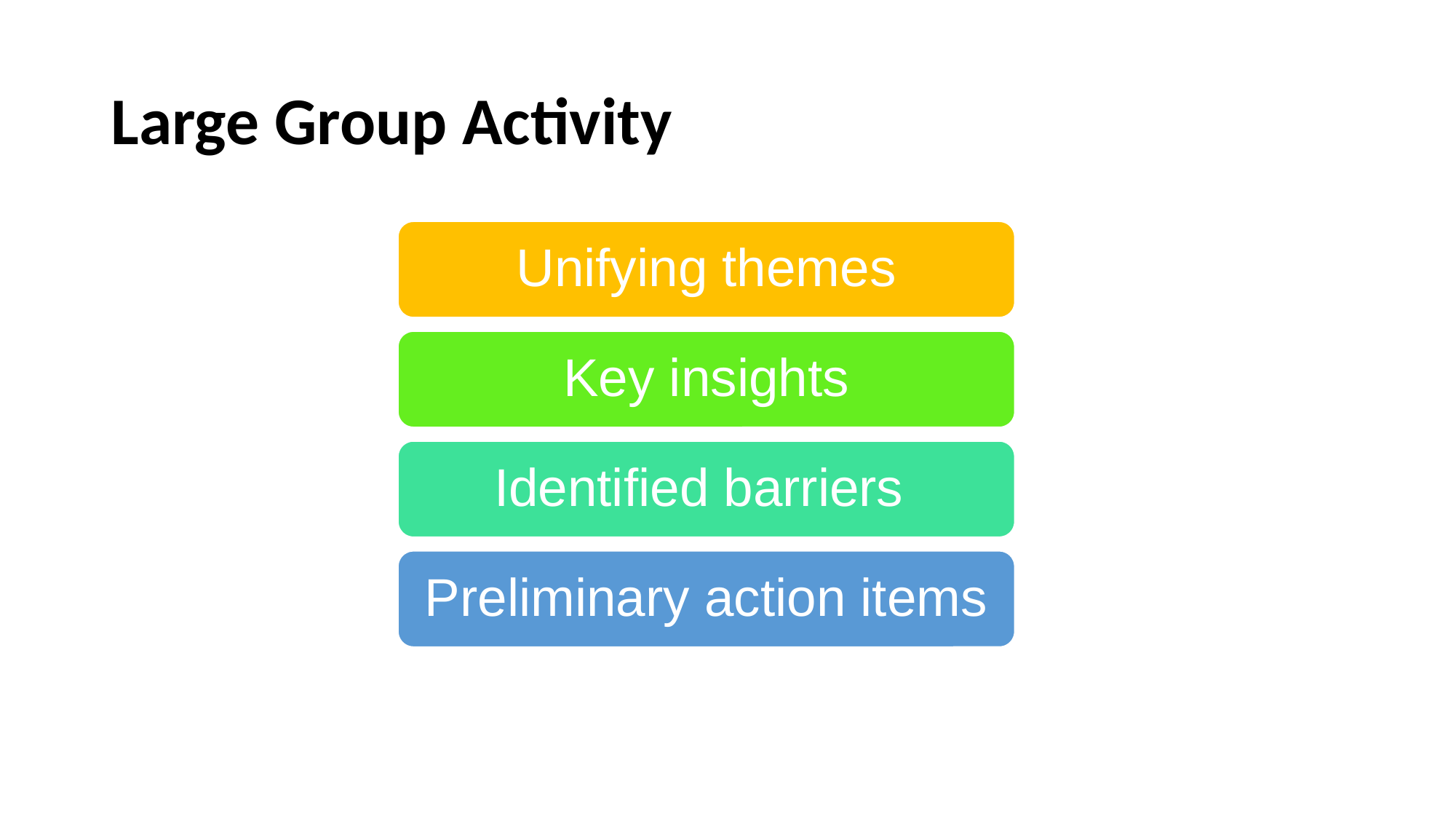

# Large Group Activity
Unifying themes
Key insights
Identified barriers
Preliminary action items

## Slide 31
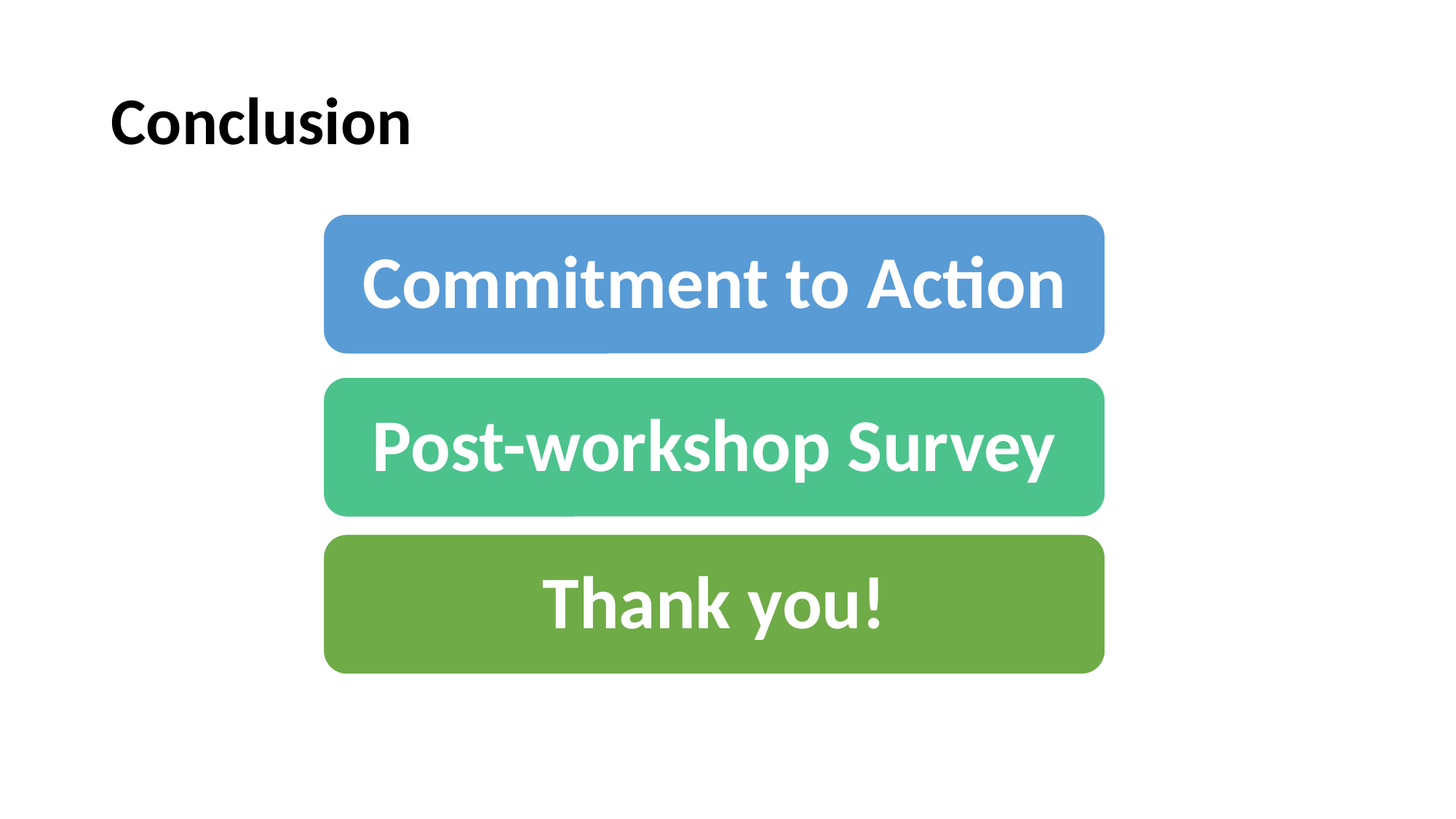

# Conclusion
Commitment to Action
Post-workshop Survey
Thank you!

## Slide 32
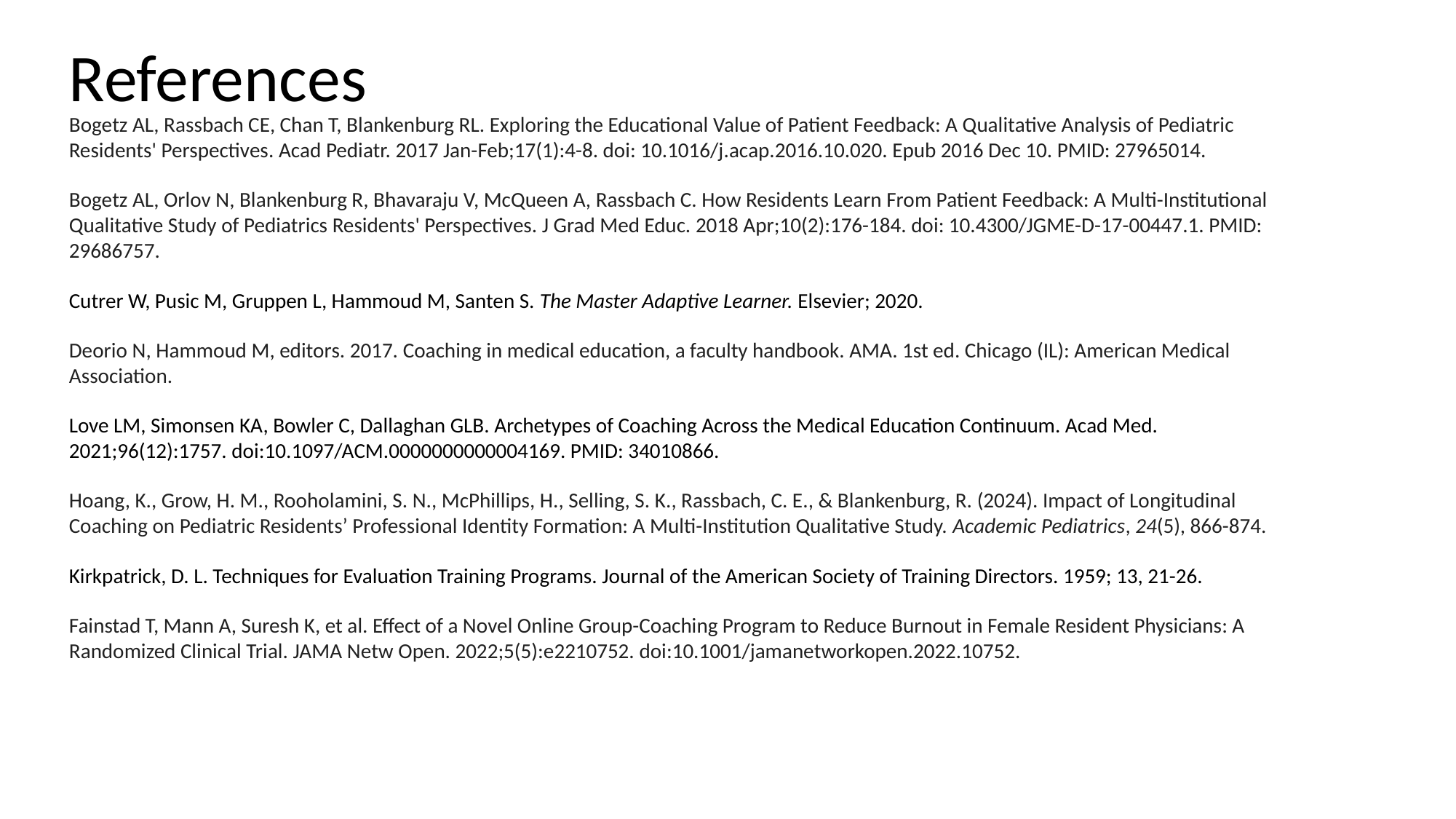

# References
Bogetz AL, Rassbach CE, Chan T, Blankenburg RL. Exploring the Educational Value of Patient Feedback: A Qualitative Analysis of Pediatric Residents' Perspectives. Acad Pediatr. 2017 Jan-Feb;17(1):4-8. doi: 10.1016/j.acap.2016.10.020. Epub 2016 Dec 10. PMID: 27965014.
Bogetz AL, Orlov N, Blankenburg R, Bhavaraju V, McQueen A, Rassbach C. How Residents Learn From Patient Feedback: A Multi-Institutional Qualitative Study of Pediatrics Residents' Perspectives. J Grad Med Educ. 2018 Apr;10(2):176-184. doi: 10.4300/JGME-D-17-00447.1. PMID: 29686757.
Cutrer W, Pusic M, Gruppen L, Hammoud M, Santen S. The Master Adaptive Learner. Elsevier; 2020.
Deorio N, Hammoud M, editors. 2017. Coaching in medical education, a faculty handbook. AMA. 1st ed. Chicago (IL): American Medical Association.
Love LM, Simonsen KA, Bowler C, Dallaghan GLB. Archetypes of Coaching Across the Medical Education Continuum. Acad Med. 2021;96(12):1757. doi:10.1097/ACM.0000000000004169. PMID: 34010866.
Hoang, K., Grow, H. M., Rooholamini, S. N., McPhillips, H., Selling, S. K., Rassbach, C. E., & Blankenburg, R. (2024). Impact of Longitudinal Coaching on Pediatric Residents’ Professional Identity Formation: A Multi-Institution Qualitative Study. Academic Pediatrics, 24(5), 866-874.
Kirkpatrick, D. L. Techniques for Evaluation Training Programs. Journal of the American Society of Training Directors. 1959; 13, 21-26.
Fainstad T, Mann A, Suresh K, et al. Effect of a Novel Online Group-Coaching Program to Reduce Burnout in Female Resident Physicians: A Randomized Clinical Trial. JAMA Netw Open. 2022;5(5):e2210752. doi:10.1001/jamanetworkopen.2022.10752.

## Slide 33
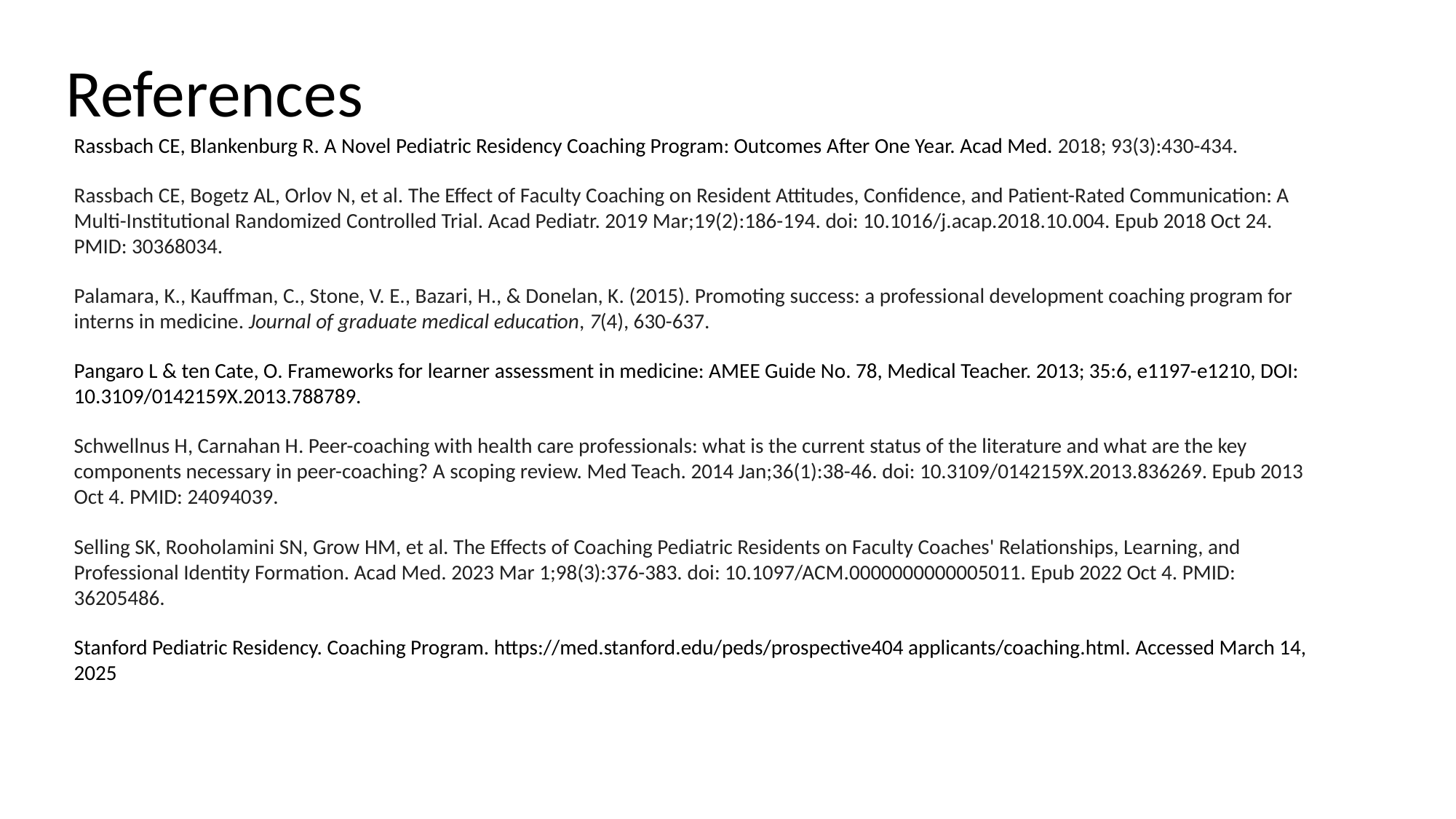

# References
Rassbach CE, Blankenburg R. A Novel Pediatric Residency Coaching Program: Outcomes After One Year. Acad Med. 2018; 93(3):430-434.
Rassbach CE, Bogetz AL, Orlov N, et al. The Effect of Faculty Coaching on Resident Attitudes, Confidence, and Patient-Rated Communication: A Multi-Institutional Randomized Controlled Trial. Acad Pediatr. 2019 Mar;19(2):186-194. doi: 10.1016/j.acap.2018.10.004. Epub 2018 Oct 24. PMID: 30368034.
Palamara, K., Kauffman, C., Stone, V. E., Bazari, H., & Donelan, K. (2015). Promoting success: a professional development coaching program for interns in medicine. Journal of graduate medical education, 7(4), 630-637.
Pangaro L & ten Cate, O. Frameworks for learner assessment in medicine: AMEE Guide No. 78, Medical Teacher. 2013; 35:6, e1197-e1210, DOI: 10.3109/0142159X.2013.788789.
Schwellnus H, Carnahan H. Peer-coaching with health care professionals: what is the current status of the literature and what are the key components necessary in peer-coaching? A scoping review. Med Teach. 2014 Jan;36(1):38-46. doi: 10.3109/0142159X.2013.836269. Epub 2013 Oct 4. PMID: 24094039.
Selling SK, Rooholamini SN, Grow HM, et al. The Effects of Coaching Pediatric Residents on Faculty Coaches' Relationships, Learning, and Professional Identity Formation. Acad Med. 2023 Mar 1;98(3):376-383. doi: 10.1097/ACM.0000000000005011. Epub 2022 Oct 4. PMID: 36205486.
Stanford Pediatric Residency. Coaching Program. https://med.stanford.edu/peds/prospective404 applicants/coaching.html. Accessed March 14, 2025

## Slide 34
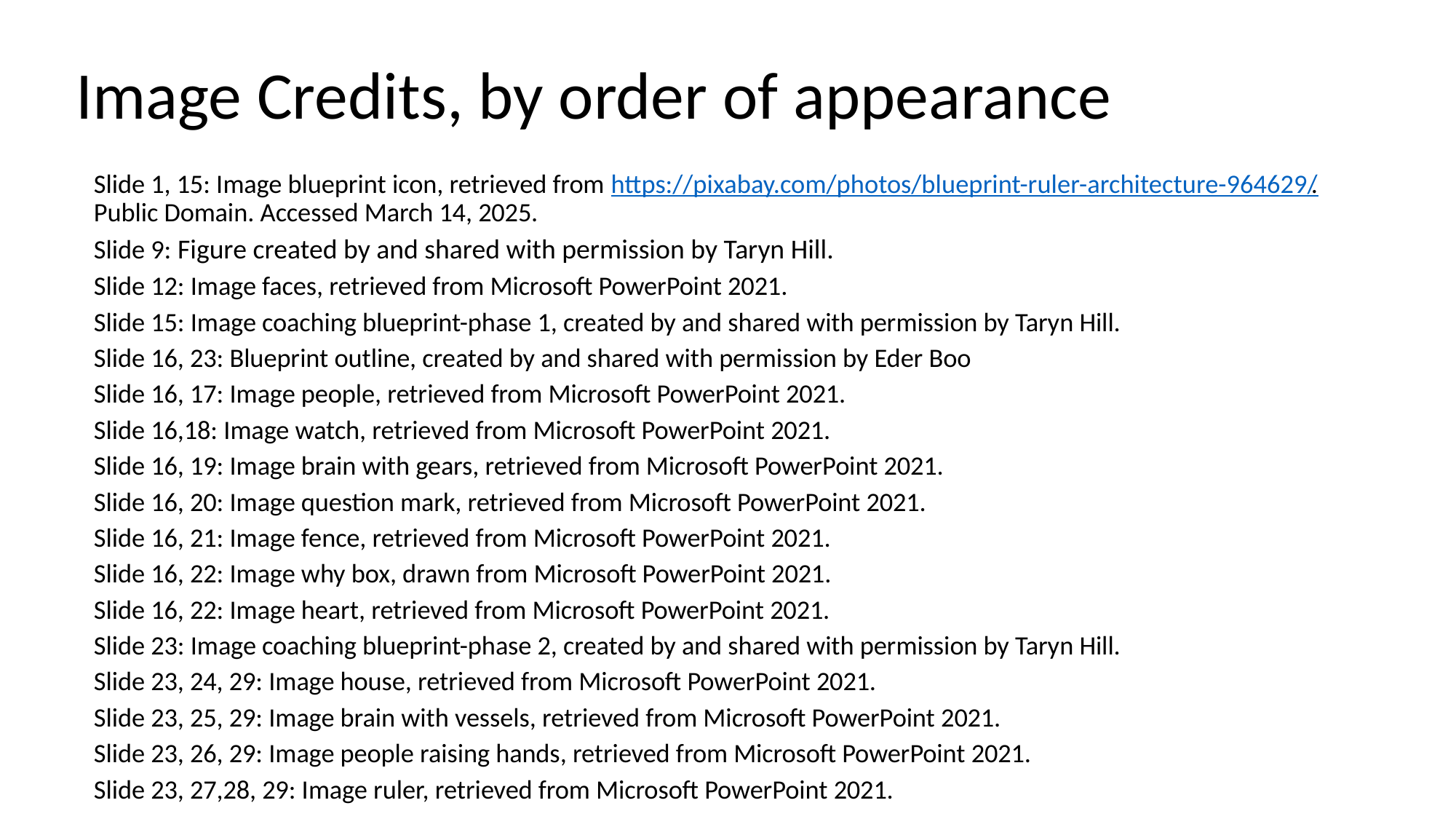

# Image Credits, by order of appearance
Slide 1, 15: Image blueprint icon, retrieved from https://pixabay.com/photos/blueprint-ruler-architecture-964629/. Public Domain. Accessed March 14, 2025.
Slide 9: Figure created by and shared with permission by Taryn Hill.
Slide 12: Image faces, retrieved from Microsoft PowerPoint 2021.
Slide 15: Image coaching blueprint-phase 1, created by and shared with permission by Taryn Hill.
Slide 16, 23: Blueprint outline, created by and shared with permission by Eder Boo
Slide 16, 17: Image people, retrieved from Microsoft PowerPoint 2021.
Slide 16,18: Image watch, retrieved from Microsoft PowerPoint 2021.
Slide 16, 19: Image brain with gears, retrieved from Microsoft PowerPoint 2021.
Slide 16, 20: Image question mark, retrieved from Microsoft PowerPoint 2021.
Slide 16, 21: Image fence, retrieved from Microsoft PowerPoint 2021.
Slide 16, 22: Image why box, drawn from Microsoft PowerPoint 2021.
Slide 16, 22: Image heart, retrieved from Microsoft PowerPoint 2021.
Slide 23: Image coaching blueprint-phase 2, created by and shared with permission by Taryn Hill.
Slide 23, 24, 29: Image house, retrieved from Microsoft PowerPoint 2021.
Slide 23, 25, 29: Image brain with vessels, retrieved from Microsoft PowerPoint 2021.
Slide 23, 26, 29: Image people raising hands, retrieved from Microsoft PowerPoint 2021.
Slide 23, 27,28, 29: Image ruler, retrieved from Microsoft PowerPoint 2021.
