# Supplementary material for: From Blueprints to Build: A Workshop for Developing a Clinical Coaching Program
Source: MedEdPORTAL. 2025 Sep 26;21:11548. doi: 10.15766/mep_2374-8265.11548 (PMC12464251; doi:10.15766/mep_2374-8265.11548)
Supplement: Supplementary file 1 — Coaching Program Development.pptxFacilitator Guide.docxCoaching Skits.docxEditable Coaching Program Blueprint.docxExample Coaching Program Blueprint - JHACH.docxExample Coaching Program Blueprint - MUSC.docxExample Coaching Program Blueprint - Stanford.docxStructured Clinical Observation Coaching Tool.docxResident Self-Reflection and Goal Setting Form.docxPostworkshop Survey.docx [file mep_2374-8265.11548-s001.zip › C. Coaching Skits.docx]

**Appendix C: Coaching Skits**

**Instructions:** Below are the two role playing scenarios acted out by facilitators. Five minutes is recommended per scenario. Each scenario may take 2-3 minutes with time to reflect following each skit. Scenario 2 highlights the application of ADAPT framework in Red.

**Summary of Scenarios:** A senior resident and attending are debriefing after resident’s failed attempt of a lumbar puncture**.** The two scenarios below juxtapose directive feedback style with a coaching approach.

**Roles:**

- **Actor 1:** Senior resident (Name: Alejandro Cruz) demonstrating fixed mindset after failing lumbar puncture.
- **Actor 2:** Attending (Name: Dr. Fatima Hassan) who coaches senior resident through an advising and effective coaching.

**Scenario 1: Directive Feedback Style.** *Attending gives Sr. Resident feedback in a directive and tell format. Attending does not help the learner self-reflect or identify areas for improvement and next steps. Attending is emotionally unsupportive.*

**1:** Dr. Hassan, I am so frustrated with myself. I am embarrassed that I was unable to get the lumbar puncture after three failed attempts. Dr. Hassan, I can’t believe that you had to finish the procedure for me.

**2:** Ok enough of that. Alejandro, I think we need to debrief on that procedure and how it went. There are a lot of things you need to improve on for LP procedures.

**1:** Yes, I agree. When you put it that way I feel like a failure.

**2**: You did not set up the sterile field properly. I didn’t hear you provide any direction to the holder and therefore the baby was moving a lot. What else do you think went wrong?

**1:** Well, geez, when you put it like that… I guess everything. I can’t think of one part of the procedure that went smoothly.

**2:** You are clearly upset, but now is not the time for pity or self-deprecation. You need to reflect on the procedure thoughtfully without having your emotions get in the way.

**1:** Ok. Well upon reflection without any emotion (said in monotone voice), I am feeling incompetent and without skill. I need to improve my LP skills.

**2:** Yes, I agree. That’s exactly what you need to do. You will need to spend some time in the simulation center practicing prior to your next lumbar puncture. Let me know if you have any questions.

*Attending exits scene and Sr. Resident is talking to himself.*

**1:** Well, thanks for the coaching Dr. Hassan (said with sarcasm). So, it sounds like I’m the worst resident ever who cannot complete a basic residency procedure. Dr. Hassan made me feel like nothing I did was right. I am not sure I want to do this anymore.

**Scenario 2:** **Effective Coaching**. *Attending is emotionally supportive and establishes good rapport prior to implementing coaching strategy. Attending uses questions to help learner self-reflect, identify errors and plan for improvement.*

**1:** I am so frustrated with myself. I am embarrassed I was unable to get the lumbar puncture after three failed attempts. Dr. Hassan, I can’t believe that you had to finish the procedure for me.

**2**: Hey Alejandro, I see you are upset. I think it is important that we debrief after that LP procedure. Is this an okay time for you to talk?

**1:** Yes…. (said with hesitancy.) It is probably a good idea.

**2:** First of all, ... Are you ok?

**1:** Not really. I feel like a failure and incompetent.

**2:** Let’s back up for a moment. I am sorry you are feeling this way. Why do you feel incompetent?

**1:** Well, I am a senior resident and I can’t successfully perform a lumbar puncture. This is a basic residency procedure. This is imposter syndrome 101!

**2:** Let’s talk about the procedure… **What went well, Alejandro? Ask**

**1:** Oh, so you are going to make me do this…I felt prepared in advance. I did a good job setting up the sterile field. We had a great holder. The baby was calm and didn’t move. Honestly, everything was going smoothly. **Discuss**

**2:** I agree. You had great preparation for the procedure. Your sterile technique was spot on! **Discuss**

**Now tell me, what part was challenging? Ask**

**1:** I had a hard time locating the landmarks. I am not even sure if my angle was accurate. And I definitely did not feel the pop! **Discuss**

**2:** What **I noticed** is that you could have provided more feedback to the holder for best positioning. I felt you were nervous. You seemed hesitant advancing the needle as you were shaking. **Discuss**

**1:** I agree (biting nails), I was super nervous and not confident with how far to advance needle. I am fearful to do a lumbar puncture again. **Discuss**

**2:** Practice and mistakes are part of residency training. The most important part is how we respond and learn from our mistakes. **So, what are your next steps from here?** **Plan Together**

**1:** I honestly think I need more practice. I have only done a few LP’s this year.

**2:** Exactly! I want to support you. **How would you like to go about that?** **Plan Together**

**1:** I would like to schedule time in the simulation center. Would you be able to observe and coach me? **Plan Together**

**2:** Of course! I think that’s an excellent goal. Let’s meet at sim center, say…next week? Would that work for you? **Plan Together**

**1:** Sounds great! Thank you. Dr. Hassan, thanks for coaching me!

**2:** I’m happy to be your coach! High Five!

**ADAPT Framework Application:**

The iterative nature of coaching cycle is demonstrated in scenario 2, which highlights effective coaching in action. Phrases labeled in **BOLD** apply to Ask-Discuss-Ask-Plan Together (ADAPT) framework utilized as a coaching tool.

- **Ask** (first): Ask learner to reflect on event.
- **Discuss:** Coach shares observations and/or provides feedback in supportive non-judgmental manner. Learner engages in self-reflection and responds. A conversation takes place about the observation.
- **Ask** (again): Ask another question, invite learner to self-reflect or respond to the feedback.
- **Plan Together:** Learner and coach work together to establish clear, actionable goals.
