# Supplementary material for: From Blueprints to Build: A Workshop for Developing a Clinical Coaching Program
Source: MedEdPORTAL. 2025 Sep 26;21:11548. doi: 10.15766/mep_2374-8265.11548 (PMC12464251; doi:10.15766/mep_2374-8265.11548)
Supplement: Supplementary file 1 — Coaching Program Development.pptxFacilitator Guide.docxCoaching Skits.docxEditable Coaching Program Blueprint.docxExample Coaching Program Blueprint - JHACH.docxExample Coaching Program Blueprint - MUSC.docxExample Coaching Program Blueprint - Stanford.docxStructured Clinical Observation Coaching Tool.docxResident Self-Reflection and Goal Setting Form.docxPostworkshop Survey.docx [file mep_2374-8265.11548-s001.zip › D. Editable Coaching Program Blueprint.docx]

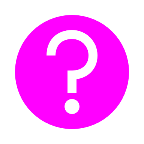

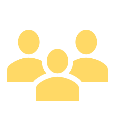


**Appendix D**


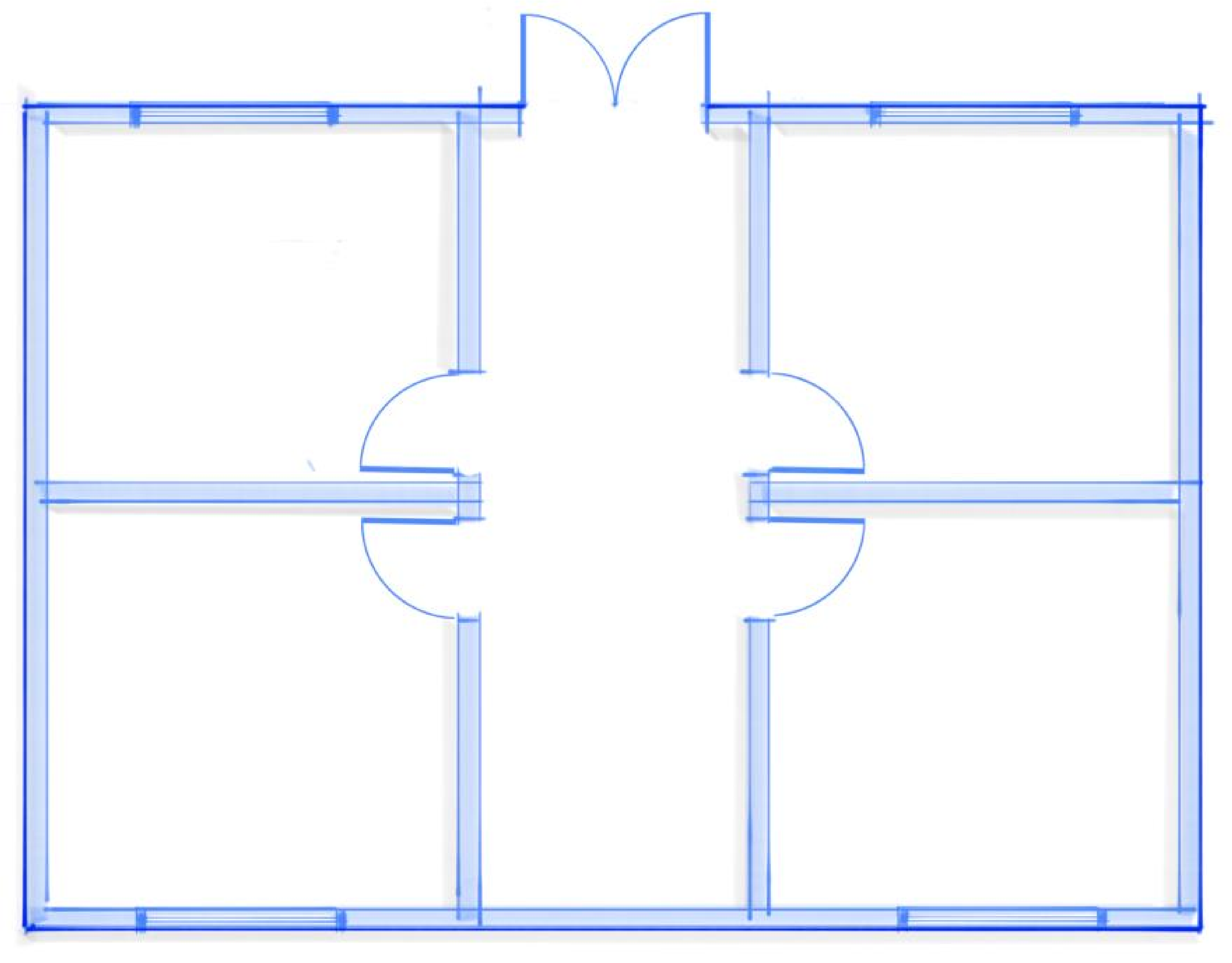


**Who?**

**Where and When?**

**What?**

**Who is a Coach**

**?**

**# of Coaches**

**:**

**Who is a**

**Coachee**

**?**

**# of**

**Coachees**

**:**

**Ratio of**

**Coachees**

**to**

**Coaches**

**Clinical Settings?**

**Frequency?**

**Time**

**Duration**

**?**

**Barriers?**

**How is coaching program funded?**

**How are faculty selected?**

**How are coaching assignments made?**

**Coaching Program**

**Coaching Blueprint Phase 1**

**Basics**

**Why?**

**Name**

**:**

**How?**

**Why is coaching important to you?**

**What types of Coaching Observations?**

**What tools for Coaching?**

**Virtual and/or In Person?**


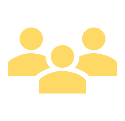


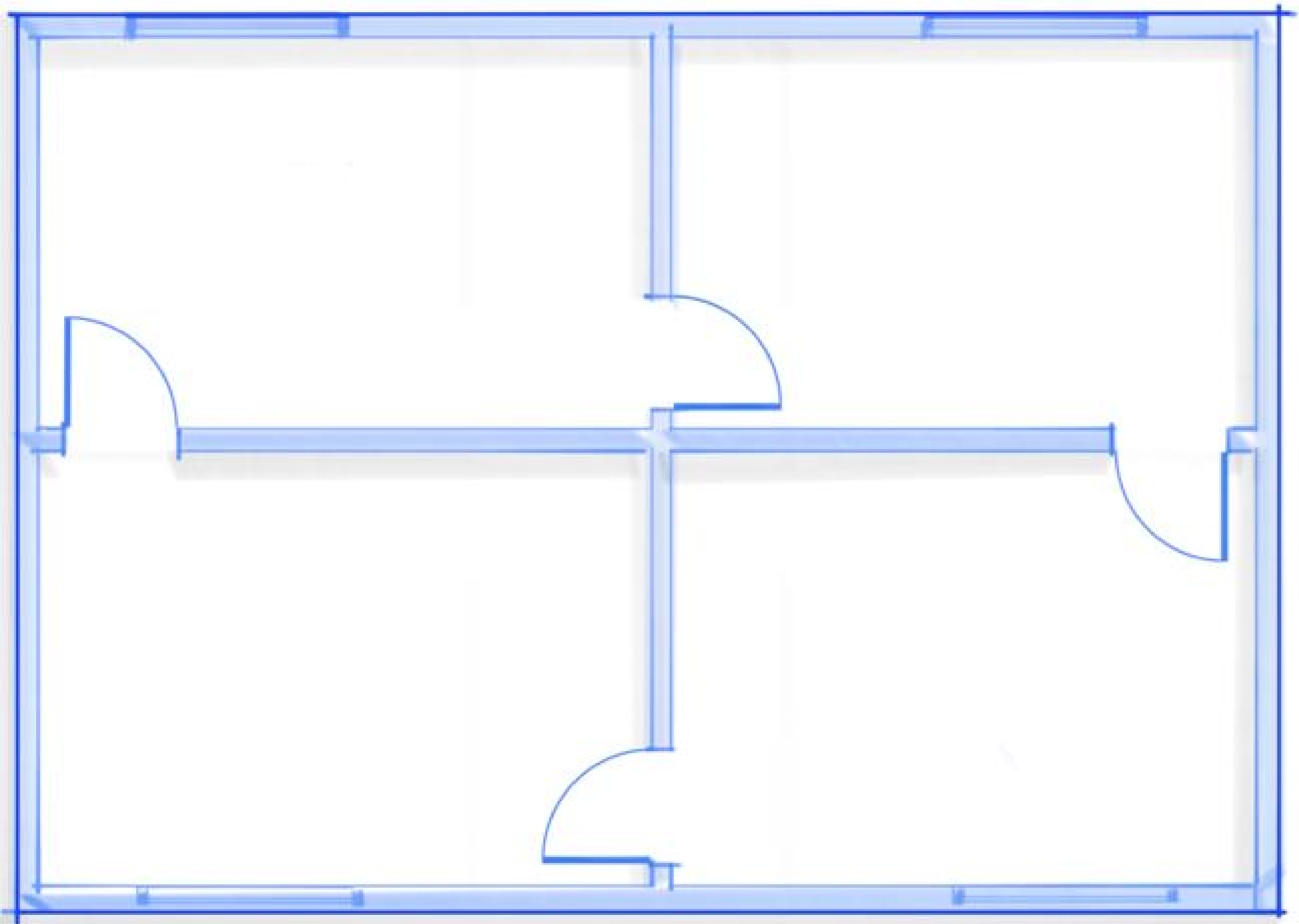


**Coaching Blueprint**

**Phase 2**

**Evaluations & Outcomes**

**Faculty Development**

**Tools for Feedback and Facilitated Reflection**

**Program Structure**

**Design, Build & Refine**

Citations:

Image coaching blueprint-phase 1 and phase 2, created by and shared with permission by Taryn Hill.

Image blueprint outline page 1 and 2, created by and shared with permission by Eder Boo.

Image “Why box” drawn from Microsoft PowerPoint 2021.

Image people, watch, brain with gears, dollar sign, fence, heart, house, brain with vessels, people raising hands, ruler retrieved from Microsoft PowerPoint 2021.
