# Supplementary material for: From Blueprints to Build: A Workshop for Developing a Clinical Coaching Program
Source: MedEdPORTAL. 2025 Sep 26;21:11548. doi: 10.15766/mep_2374-8265.11548 (PMC12464251; doi:10.15766/mep_2374-8265.11548)
Supplement: Supplementary file 1 — Coaching Program Development.pptxFacilitator Guide.docxCoaching Skits.docxEditable Coaching Program Blueprint.docxExample Coaching Program Blueprint - JHACH.docxExample Coaching Program Blueprint - MUSC.docxExample Coaching Program Blueprint - Stanford.docxStructured Clinical Observation Coaching Tool.docxResident Self-Reflection and Goal Setting Form.docxPostworkshop Survey.docx [file mep_2374-8265.11548-s001.zip › E. Example Coaching Program Blueprint - JHACH.docx]

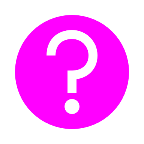

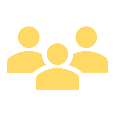


**Appendix E**

Clinical Observations: H&P on wards, Sick visit and well child check in clinic

Educational Observations: Morning reports and case conferences


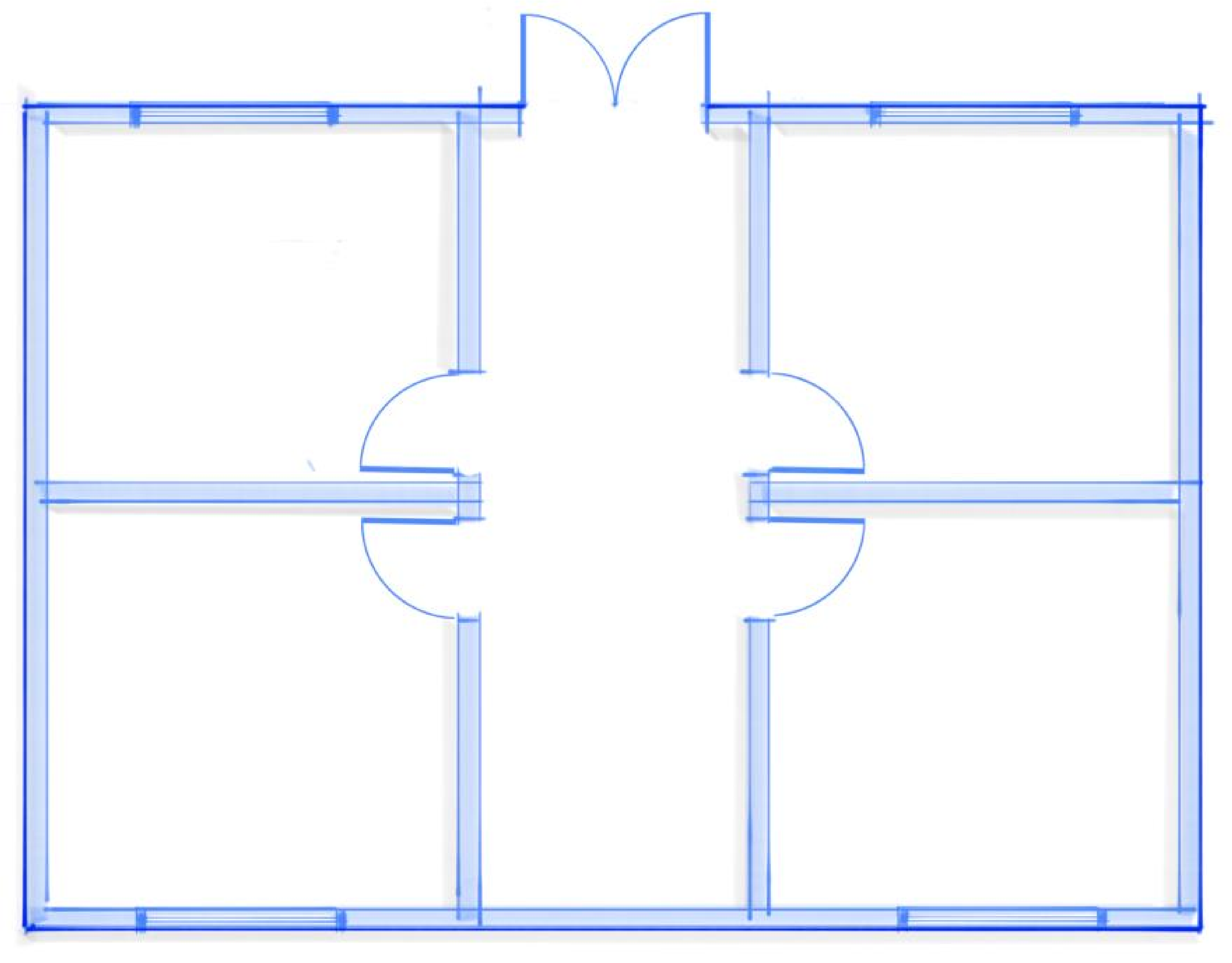


**Who?**

**Where and When?**

**What?**

**Who is a Coach**

**?**

**# of Coaches**

**:**

**Who is a**

**Coachee**

**?**

**# of**

**Coachees**

**:**

**Ratio of**

**Coachees**

**to**

**Coaches**

**Clinical Settings?**

**Frequency?**

**Time**

**Duration**

**?**

**Barriers?**

**How is coaching program funded?**

**How are faculty selected?**

**How are coaching assignments made?**

**Coaching**

**Program**

**Coaching Blueprint**

**–**

**Phase 1**

**Basics**

**Why?**

**How?**

**Why is coaching important to you?**

**What types of Coaching Observations?**

**What Tools for Coaching?**

**Virtual and/or In Person?**

1. FTE for Coaching Directors

No FTE for Faculty Coaches

Random assignment and mostly based on faculty schedule.

Volunteer based. Offered to faculty who have an interest in observing interns.

**Johns Hopkins All Children’s Hospital Pediatric Residency Coaching Program**

General Pediatricians

Hospitalist

ER

NICU

15 faculty coaches

Promotes longitudinal growth of residents. Encourages residents to improve, reflect and grow in all domains of residency. Rewarding as faculty!

Roughly 2:1 because small scale program and few observations annually.

12 PGY1

12 PGY2

PGY1 and PGY2

30 minutes to 2 hours

PGY1: Twice annually in CC clinic; Twice annually on inpatient wards

Outpatient Continuity Clinic (CC)

Inpatient Wards

Funding

Faculty Motivation

Schedule

Time Commitment

Both virtual (zoom) and in person

Structured Clinical Observation Tools for inpatient and outpatient (modeled after Stanford and JHU tools)

b


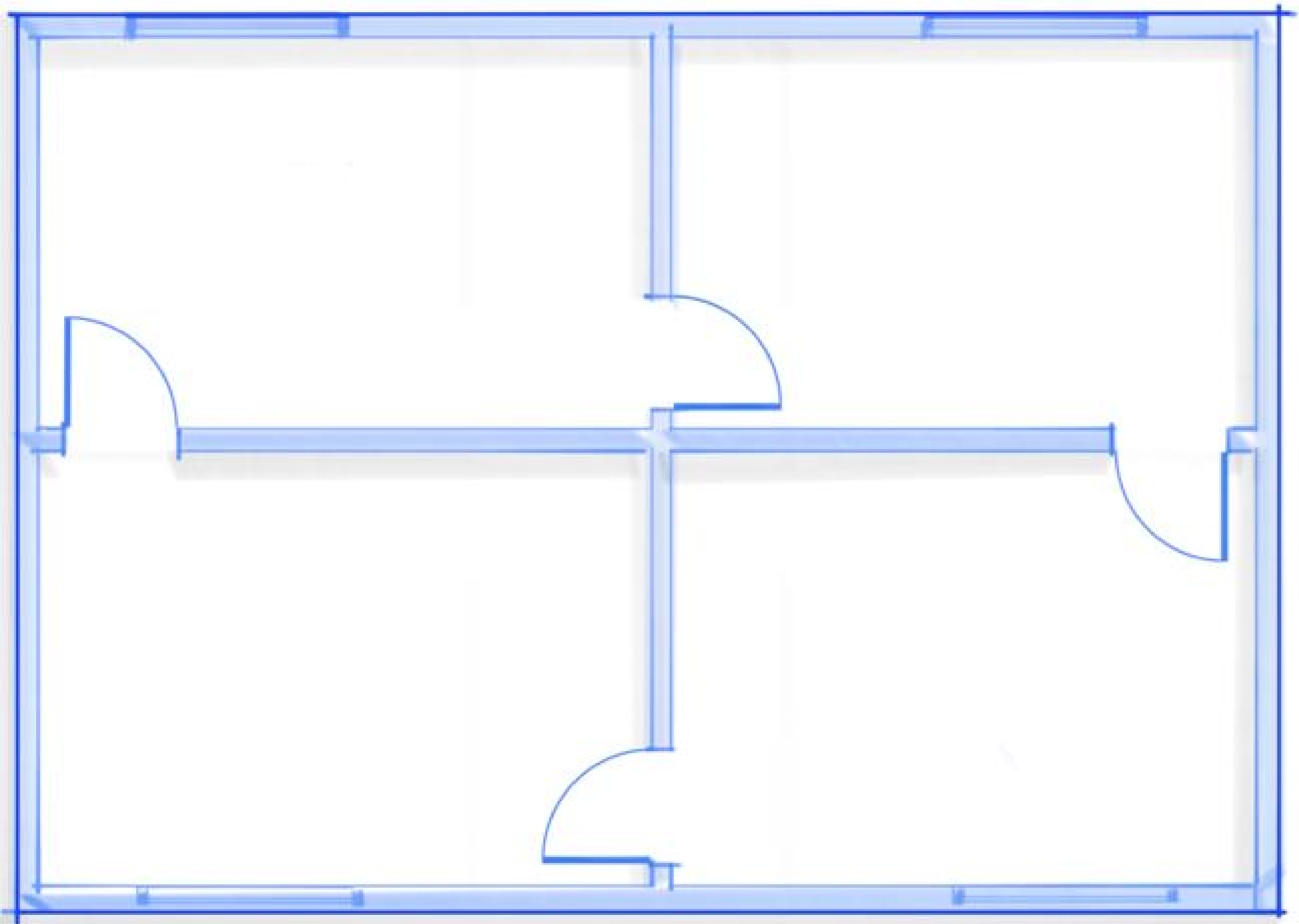


**Coaching Blueprint**

**-**

**Phase 2**

**Evaluations & Outcomes**

**Faculty Development**

**Program**

**Structure**

**and**

**Observations**

**Design, Build & Refine**

**Tools for Feedback and Facilitated Reflection**

- We have surveyed faculty and interns after 1 year completion of pilot coaching program with data pending.
- Evaluation of coach and coachee perspective of how coaching influenced clinical, communication and educational skills.
- Provide monthly faculty development sessions on coaching principles, leadership, master adaptive learner, wellness, professional identity formation, etc.
- Faculty meets with intern following each clinical observation to facilitate learner self-reflection. Faculty provide feedbacks through deliberate coaching strategies (Ask-Tell-Ask ).
- Faculty trained in helping the learner identify areas of improvement through questions.
- Longitudinal Coaching Program launched in 2022.
- Each intern is assigned to one coach who performs all clinical and educational observations throughout the academic year for all 3 years of residency.
- Each faculty assigned to one intern each year and has a total of 3 residents at one time.
- Faculty coaches observe each PGY1 perform two general pediatrics visits (well child/sick visit) and two inpatient H&P’s.
- Faculty coaches observe biannual morning reports and annual case conferences.

Citations:

Image coaching blueprint-phase 1 and phase 2, created by and shared with permission by Taryn Hill.

Image blueprint outline page 1 and 2, created by and shared with permission by Eder Boo.

Image “Why box” drawn from Microsoft PowerPoint 2021.

Image people, watch, brain with gears, dollar sign, fence, heart, house, brain with vessels, people raising hands, ruler retrieved from Microsoft PowerPoint 2021.
