# Supplementary material for: From Blueprints to Build: A Workshop for Developing a Clinical Coaching Program
Source: MedEdPORTAL. 2025 Sep 26;21:11548. doi: 10.15766/mep_2374-8265.11548 (PMC12464251; doi:10.15766/mep_2374-8265.11548)
Supplement: Supplementary file 1 — Coaching Program Development.pptxFacilitator Guide.docxCoaching Skits.docxEditable Coaching Program Blueprint.docxExample Coaching Program Blueprint - JHACH.docxExample Coaching Program Blueprint - MUSC.docxExample Coaching Program Blueprint - Stanford.docxStructured Clinical Observation Coaching Tool.docxResident Self-Reflection and Goal Setting Form.docxPostworkshop Survey.docx [file mep_2374-8265.11548-s001.zip › F. Example Coaching Program Blueprint - MUSC.docx]

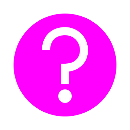

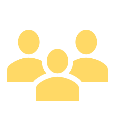


**Appendix F**

Coordinated within the residency program’s learning community model

Funding/time

Sometimes buy in from learners

Celebrates growth mindset as an important component of lifelong learning and skill development. Love the focus on longitudinal relationship formation.

Average 2 interns: 1 coach

PGY1: categorical pediatrics, pediatric primary care, pediatric neurology, med/peds. 22 interns per year.

12

PGY1: Clinical observations on wards, ED, clinic

PGY2&3: Focused feedback on teaching sessions

In person

Rubrics for clinical observations

Faculty development workshop series

Coaches receive ‘citizenship points’ which can be used as credits in place of otherwise required institutional faculty activities

Nomination and general review by coaching team to assure fit

Avg 30 min – 60 min per coaching session

Goal: five times per year

Inpatient wards, ED, clinic


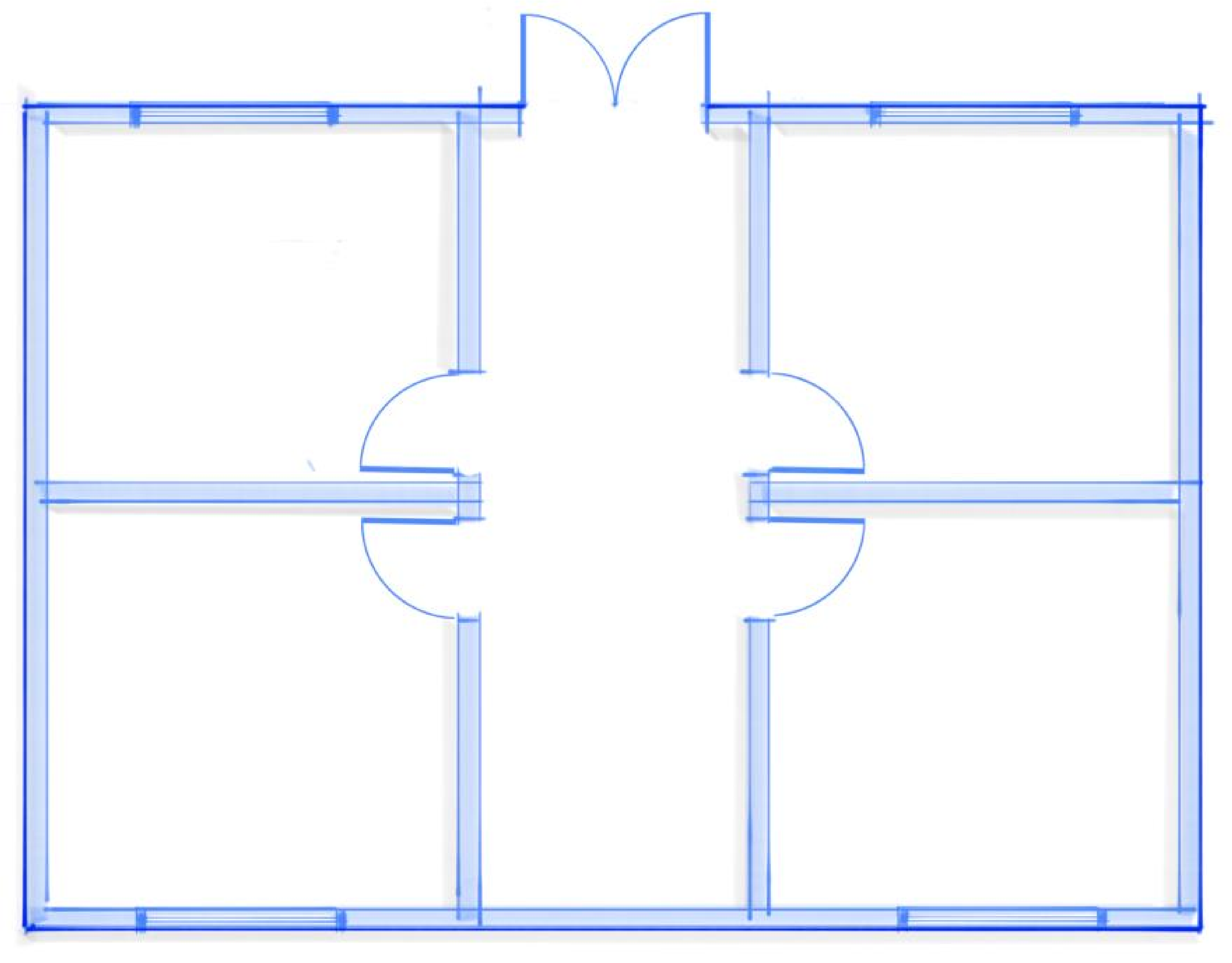


**Who?**

**Where and When?**

**What?**

**Who is a Coach**

**?**

**# of Coaches**

**:**

**Who is a**

**Coachee**

**?**

**# of**

**Coachees**

**:**

**Ratio of**

**Coachees**

**to**

**Coaches**

**Clinical Settings?**

**Frequency?**

**Time**

**Duration**

**?**

**Barriers?**

**How is coaching program funded?**

**How are faculty selected?**

**How are coaching assignments made?**

**Coaching Program**

**Coaching Blueprint Phase 1**

**Basics**

**Why?**

**Name**

**:**

**How?**

**Why is coaching important to you?**

**What types of Coaching Observations?**

**What tools for Coaching?**

**Virtual and/or In Person?**

Faculty, PHM and PEM fellows

**Medical University of**

**South Carolina**


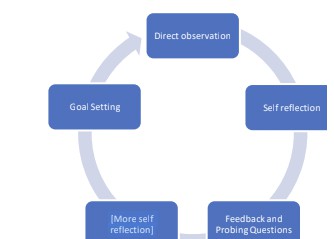


Coaches are integrated in a learning community structure within the residency program.

Coaches work clinically with PGY1 coachees on communication, efficiency, and clinical reasoning in various clinical settings.

PGY 2 & PGY 3 receive specific coaching related to teaching skills.

Coaching encounters are formative and focus on guided self-reflection, targeted feedback and goal setting. Coaching forms are not included in resident assessment or Clinical Competency Committee (CCC) evaluation.


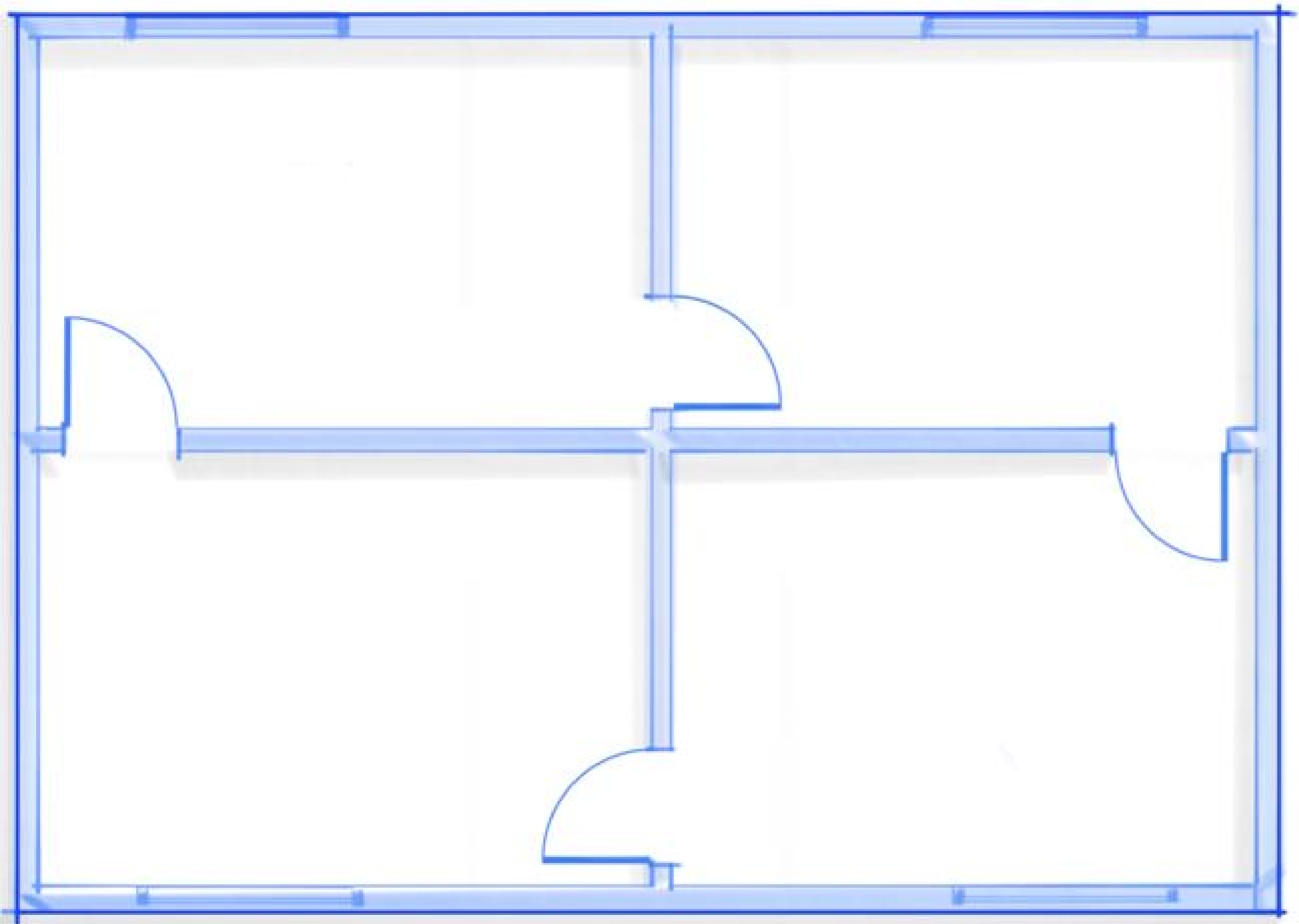


**Coaching Blueprint**

**Phase 2**

**Evaluations & Outcomes**

**Faculty Development**

**Tools for Feedback and Facilitated Reflection**

**Program Structure**

c

**Design, Build & Refine**

Collecting data on feasibility. (Number of coaching sessions), qualitative feedback from residents and coaches. Trending ACGME program and internal program data related to satisfaction with feedback received.

Longitudinal faculty development workshop series: every other month for 1.5 hours. Training topics include the components of the coaching cycle as well as topics suggested by coaches.

Intern development workshop series: annually, 3 sessions over the first 3 months of intern year for all interns:

- Introduction to coaching
- Receiving feedback
- Self-reflection and setting goals

Follow the coaching cycle. Emphasize Ask Discuss Ask Plan Together (ADAPT) or ask-tell-ask framework for delivering feedback.

Citations:

Image coaching blueprint-phase 1 and phase 2, created by and shared with permission by Taryn Hill.

Image blueprint outline page 1 and 2, created by and shared with permission by Eder Boo.

Image “Why box” drawn from Microsoft PowerPoint 2021.

Image people, watch, brain with gears, dollar sign, fence, heart, house, brain with vessels, people raising hands, ruler retrieved from Microsoft PowerPoint 2021.
