# Supplementary material for: From Blueprints to Build: A Workshop for Developing a Clinical Coaching Program
Source: MedEdPORTAL. 2025 Sep 26;21:11548. doi: 10.15766/mep_2374-8265.11548 (PMC12464251; doi:10.15766/mep_2374-8265.11548)
Supplement: Supplementary file 1 — Coaching Program Development.pptxFacilitator Guide.docxCoaching Skits.docxEditable Coaching Program Blueprint.docxExample Coaching Program Blueprint - JHACH.docxExample Coaching Program Blueprint - MUSC.docxExample Coaching Program Blueprint - Stanford.docxStructured Clinical Observation Coaching Tool.docxResident Self-Reflection and Goal Setting Form.docxPostworkshop Survey.docx [file mep_2374-8265.11548-s001.zip › G. Example Coaching Program Blueprint - Stanford.docx]

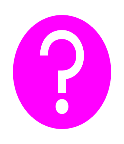

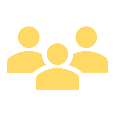


**Appendix G**


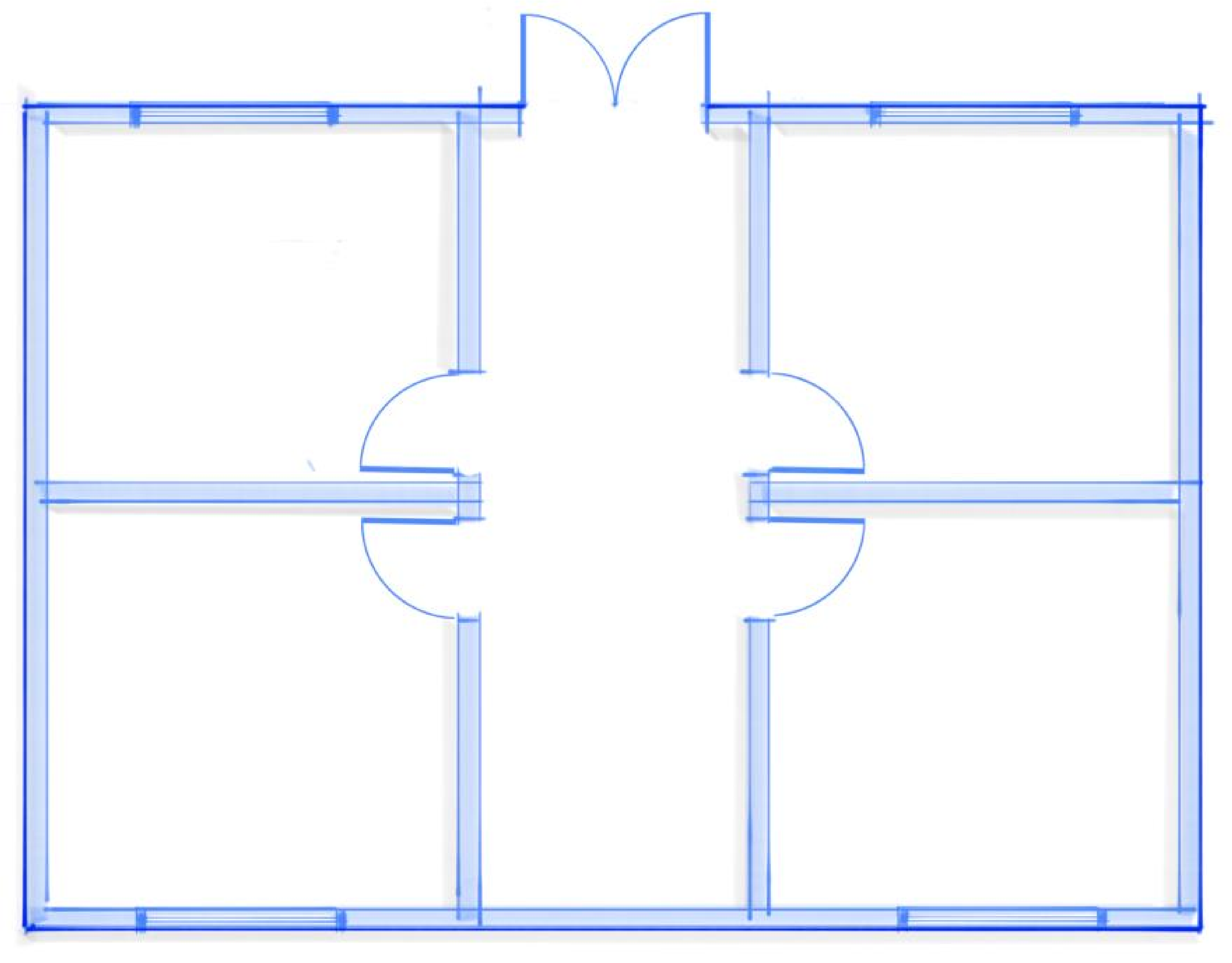


**Who?**

**Where and When?**

**What?**

**Who is a Coach**

**?**

**# of Coaches**

**:**

**Who is a**

**Coachee**

**?**

**# of**

**Coachees**

**:**

**Ratio of**

**Coachees**

**to**

**Coaches**

**Clinical Settings?**

**Frequency?**

**Time**

**Duration**

**?**

**Barriers?**

**How is coaching program funded?**

**How are faculty selected?**

**How are coaching assignments made?**

**Coaching**

**Program**

**Coaching Blueprint**

**–**

**Phase 1**

**Basics**

**Why?**

**Name**

**:**

**How?**

**Why is coaching important to you?**

**What types of Coaching Observations?**

**What Tools for Coaching?**

**Virtual and/or In Person?**

9-10 Coachees per Coach

Match with coaches they will otherwise work with early on; match with coach not in their desired field of specialty.

Application process, interviews using structured interview questions and roleplays, review evaluations from learners.

Funding from Stanford Children’s Hospital

PGY1: 1x / month

PGY2-5: 1x / 2 months

All pediatrics and combined pediatrics residents (Categorical Peds, Child Neuro, Peds Genetics, Combined Peds and Anesthesia, and Research)

12

Faculty

Both in-person and virtual

Helps learners develop growth mindset, self-reflection skills, and sense of belonging. Helps learners develop clinical skills.

20 minutes to 2 hours

All (Wards, ICU, ED, Clinic, Consults, Handoffs, Care Conferences, Teaching Sessions)

Time

Scheduling

Rubrics for clinical observations

Faculty Development

Clinical observations (wards, ICU, ED, clinic), care conferences, teaching sessions.

**Stanford Coaching Program**

f


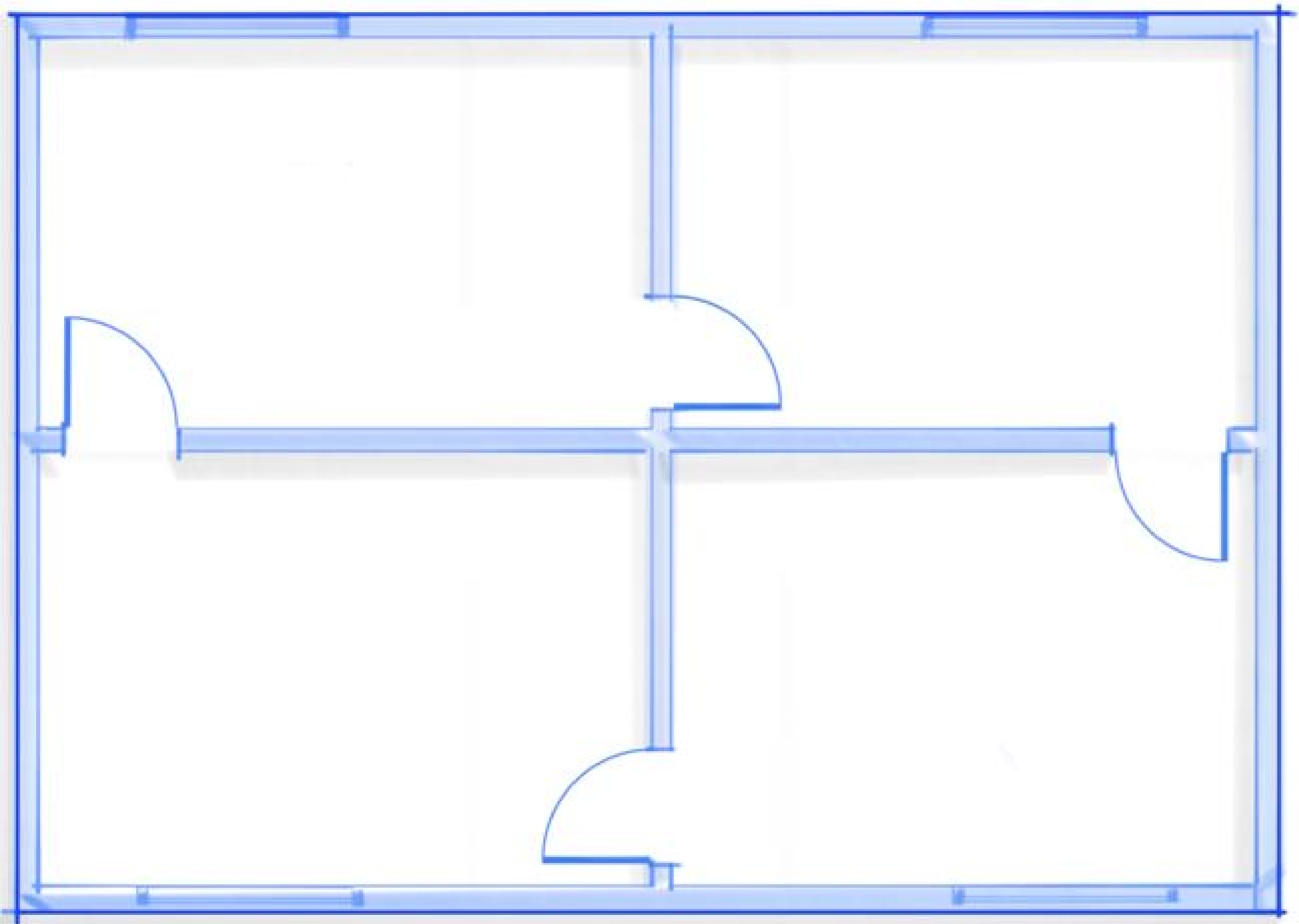


**Coaching Blueprint**

**-**

**Phase 2**

**Evaluations & Outcomes**

**Faculty Development**

**Program**

**Structure**

**and**

**Observations**

Coaches work with coachees all years of residency (1x/month as a PGY1, 1x/2 months as PGY2-5).

Formative role – facilitate reflection, give feedback, and support goal-setting.

Attend Clinical Competency Committee (CCC) for shared mental model, but not in voting capacity.

Observation tools (optional) available at:

- Stanford University School of Medicine. Coaching program for prospective pediatric applicants. Stanford Medicine. Accessed March 14, 2025.<https://med.stanford.edu/peds/prospective-applicants/coaching.html>*.(Optional)*

**Tools for Feedback and Facilitated Reflection**

Faculty Development every month. We meet for 2 hours each month (1 hour to discuss challenging coaching cases, 1 hour for faculty development session). Coaches select which faculty development sessions they want.

Faculty development (optional) resources:

- Stanford University School of Medicine. Coaching program for prospective pediatric applicants. Stanford Medicine. Accessed March 14, 2025.<https://med.stanford.edu/peds/prospective-applicants/coaching.html>*. (Optional)*

**Design, Build & Refine**

Stanford Coaching program found that coaching:

1. Improved resident feedback (more reflection, goal-setting).
2. Did not decrease feedback from other faculty.
3. Improved residents considering discordant patient feedback and increased residents wanting patient feedback.
4. Improved teams’ abilities to do shared decision-making.
5. Improved Coachees’ Professional Identity Formation.
6. Improved Professional Identity Formation, engagement, learning, and relationships in coaches.

Citations:

Image coaching blueprint-phase 1 and phase 2, created by and shared with permission by Taryn Hill.

Image blueprint outline page 1 and 2, created by and shared with permission by Eder Boo.

Image “Why box” drawn from Microsoft PowerPoint 2021.

Image people, watch, brain with gears, dollar sign, fence, heart, house, brain with vessels, people raising hands, ruler retrieved from Microsoft PowerPoint 2021.

Image in Tools for Feedback and Facilitated Reflection: **Rassbach CE, Blankenburg R.** The Stanford Pediatric Residency Coaching Program: outcomes after one year. Acad Med. 2018;93(3):429-434. doi:10.1097/ACM.0000000000001916.

Stanford University School of Medicine. Coaching program for prospective pediatric applicants. Stanford Medicine. Accessed March 14, 2025.<https://med.stanford.edu/peds/prospective-applicants/coaching.html>*. (Optional)*
