# Supplementary material for: From Blueprints to Build: A Workshop for Developing a Clinical Coaching Program
Source: MedEdPORTAL. 2025 Sep 26;21:11548. doi: 10.15766/mep_2374-8265.11548 (PMC12464251; doi:10.15766/mep_2374-8265.11548)
Supplement: Supplementary file 1 — Coaching Program Development.pptxFacilitator Guide.docxCoaching Skits.docxEditable Coaching Program Blueprint.docxExample Coaching Program Blueprint - JHACH.docxExample Coaching Program Blueprint - MUSC.docxExample Coaching Program Blueprint - Stanford.docxStructured Clinical Observation Coaching Tool.docxResident Self-Reflection and Goal Setting Form.docxPostworkshop Survey.docx [file mep_2374-8265.11548-s001.zip › B. Facilitator Guide.docx]

**Appendix B: Facilitator Guide- Framework for Workshop Minutes**

**Pre-Workshop Preparation:**

- Facilitators: Recommend a minimum of 4-8 facilitators. 1-2 per table for small group activity.
  - All facilitators review Appendix A-D in detail.
  - Since facilitators will be assigned to one of the four structural components, corresponding resources and appendices are outlined below for review prior to the workshop.
- Materials:
  - Tables: Minimum of 4 tables; no more than 10 chairs per table. Room setup detailed below.
  - Print materials prior to the workshop:
    - Facilitator guide (Appendix B) for reference throughout the workshop for each facilitator.
    - Script for Skit (Appendix C) for each of the facilitators who will participate in the role play scenario demonstration.
    - Editable coaching blueprints (Appendix D) for each participant: Phase 1 on front, Phase 2 on back.
    - Appendices E-J: Enough for all facilitators and participants.

**Instructions:** Please follow the detailed workshop timeline, including the recommended time durations, materials, and activity descriptions.

| **105-minute version** | **Time** | **Slides** | **Materials** | **Activity Description** |
| --- | --- | --- | --- | --- |
| **Introduction: Objectives, Polling and “Think-Pair-Share”** | 0-5 minutes | 3-5 | Appendices A, B | Overview of learning objectives. (Slide 3)   - Identify the purpose and benefits of coaching in health professions education. - Examine the main structural components, facilitators and barriers in developing a coaching program. - Design a coaching program blueprint relevant to your institution.   Participants complete polling questions of individual coaching experiences: (Slide 4)   - “Have you been coached in sport or activity?” - “Have you been coached professionally in your career?” - “Have you coached anyone in a sport or activity?” - “Have you coached anyone professionally in their career?”   After the poll, participants engage in a “think-pair share” (slide 5) about their best coaching experiences and the key attributes that made them effective. Following paired discussion, a few participants will share their reflections with the larger group. |
| **Brief didactic:**  **Coaching in Medical Education and Coaching Cycle** | 5-15 minutes | 6-10 | Appendices A, B | The workshop transitions to a core didactic session which provides a foundational overview of coaching in medical education. This didactic segment begins with defining coaching and reviewing how it is increasingly recommended by professional medical organizations as a strategy to support lifelong learning and professional identity formation. This is followed by a brief literature review that compares and contrasts the academic roles of coaching, mentoring and advising. A table in Appendix A (slide 9) illustrates these roles by outlining the relationship type, learner versus faculty driven and feedback style. The main benefits of coaching are highlighted with a focus on supporting learning development.  The coaching cycle (slide 10) is then introduced to illustrate an iterative process to promote lifelong learning and growth mindset. Similar to a Plan, Do, Study, Act cycle (PDSA) for continuous process improvement in quality improvement work, the coaching cycle parallels the PDSA cycle’s message of continuous and ongoing study to promote growth and development. The coaching cycle begins with goal setting in which learners are encouraged to define clear and specific objectives. *(Plan)* After goal setting, a learner is directly observed in a clinical or educational setting. *(Do)* A coach then facilitates active self-reflection and may probe further with questions and give feedback. *(Study)* The Ask, Discuss, Ask, Plan Together framework (ADAPT) framework is a coaching tool that can be applied during the “study” steps of the PDSA coaching cycle. ^23^ (slide 11) Lastly, self-reflection is encouraged again followed by active goals setting. *(Act)* |
| **Role Playing Scenarios:** | 15-25 minutes | 12 | Appendix C  Two chairs at front of room positioned across from one another | To further juxtapose the main distinctions between a directive feedback and coaching approach, facilitators prepare in advance and role play the two clinical scenarios. Full written scripts are provided in the coaching skits (Appendix C). Facilitators sit in two chairs facing each other at the front of the room, simulating a realistic clinical debrief setting. The two role playing scenarios portray a senior resident and attending who are debriefing after a resident’s failed attempt of a lumbar puncture**.**  The first scenario depicts a directive feedback approach, where the attending focuses on primarily giving the learner feedback rather than asking questions or facilitating self-reflection.  The second scenario demonstrates effective coaching through thoughtful questioning, guiding the resident in self-reflection and goal setting, while applying the ADAPT framework^22^ to structure the coaching process. The iterative nature of the coaching cycle is highlighted in scenario two which reinforces the didactics presented and illustrates coaching in action.  Each scenario is allotted five minutes. While the performance may take only 2-3 minutes, additional time is included to allow for group reflection, if time permits.  Additional video resource similar to our skits compares inexperienced coaching versus experience coaching:  American Medical Association. *Coaching in Medical Education*. AMA Ed Hub. Accessed June 18, 2025.<https://edhub.ama-assn.org/change-med-ed/video-player/18684559> |
| **Large Group Discussion: Exploring Coaching Structures Across National Programs:** | 25-35 minutes | 13-14 | Appendices A, B | Participants review and discuss the structures and aims of different coaching programs in graduate medical education. This includes coaching programs at Stanford University, Johns Hopkins All Children’s Hospital, University of Washington and University of Kentucky. In Appendix A, slides 13 and 14 outline the various coaching programs. Through guided questions and open dialogue, participants collaboratively examine different models and share how these approaches might inform the development of their own programs. The purpose of this activity is to explore different approaches on how to structure and develop specific aims for a future coaching program.  Question to guide discussion:   - What do you observe about the type and settings of program aims? - What do most programs have in common in terms of aims and big picture? - Are there specific components you would like to implement in a future coaching program? |
| **Coaching Blueprint: Phase 1** | 35-45 minutes | 15-22 | Appendices A,B,D  Printed out coaching blueprints (Appendix D)  Writing utensils for all participants | This portion of the workshop focuses on **Coaching Blueprint Phase 1**, where participants are provided the editable coaching blueprint (Appendix D). Participants will systematically explore each of the 4 quadrants of coaching program development including the what, where, when and how of program development. A brief didactic session (5 minutes) in Appendix A will provide guidance on core information to place in these boxes as participants start to brainstorm their own coaching program logistics. The presenter will review examples of completion of quadrants (from the example JHACH blueprint Appendix E) in the slides 17-21 and encourage participants to start completing their own blueprint with ideas specific to their own institution and program building vision.  The four quadrants on the blueprint are explained in detail in Appendix A and participants are following along by taking notes and brainstorming on their personal blueprint (Appendix D).   - The “**Who**” quadrant, located in the upper left, focuses on identifying types of coaches and coachees, along with considerations of appropriate coach to coachee ratios. (Slide 17) - The “**Where and When**” quadrant, located in the upper right, demonstrates various clinical settings, frequency and duration of future coaching opportunities. (Slide 18) - The “**What**” quadrant, located in the lower left, illustrates the different types of coaching observations, coaching tools and assessment tools. (Slide 19) - The “**How**” quadrant, located in lower right, addresses coaching program sustainability including funding, faculty support, faculty selection and coaching assignments. (Slide 20)   In the middle of the blueprint, there are two boxes labeled as “Barriers” located at the bottom and “Why” of coaching program development located in the middle. (Appendix D)   - The “**Barrier**” box, located in the bottom center, depicts the primary barriers to coaching program development such as funding, faculty engagement, scheduling challenges and time commitment. (Slide 21) - The “**Why**” box, located in the center and at the heart of blueprints encourages participants to brainstorm why coaching is important to us individually and as educators. This last box sets the stage for subsequent interactive think-pair-share activity. (Slide 22)   Participants are then given additional 5 minutes to finish completing phase 1 of their blueprint (Appendix D) with preliminary ideas for their coaching program design and implementation. |
| **Think-Pair-Share** | 45-50 minutes | 22 | Appendices A, B, D | Participants will engage in a think-pair-share at their table to reflect on why coaching is meaningful to them. Participants will notate this at the center of their blueprint. Attendees are encouraged to share aloud any unifying themes related to coaching such as fostering longitudinal growth and providing rewarding experiences for faculty. |
| **Coaching Blueprint: Phase 2:** | 50-60 minutes | 23-28 | Appendices A, B, D  Printed out coaching blueprints (Appendix D)- opposite side | The workshop session transitions to the main activity, the **Coaching Blueprint Phase 2** which includes the four main structural components including program structure, tools for feedback and facilitated reflection, faculty development and evaluations and outcomes. Participants will flip their editable blueprint (Appendix D) to the back side to begin taking notes on the different structural components.  Below is a broad overview of four main structural components, including main aims, resources and guiding questions for the subsequent small group activity which are also highlighted in slides 24-28.  **Program Structure:** (Slide 24)   - **Main Aims:**    - Design a coaching program adaptable to the needs of institutions of varying size and structures.   - Discuss the implementation of coaching encounters across multiple clinical and educational domains. - **Resources:** Participants can review three examples of completed blueprints:   - Example Coaching Program Blueprint-JHACH (Appendix E)   - Example Coaching Program Blueprint-MUSC (Appendix F)   - Example Coaching Program Blueprint-Stanford (Appendix G) - **Guided Questions:**    - Which clinical and educational domains are best suited for implementing coaching encounters in your setting?   - After reviewing example blueprints (Appendices E-G), what elements could be adapted or scaled to fit your coaching program?   - In what ways can coaching be integrated seamlessly into existing educational and clinical workflows without overburdening faculty or learners?   **2. Tools for Feedback and Facilitated Reflection:** (Slide 25)   - **Main Aims:**    - Conduct group sharing of various coaching tools, including clinical observation forms, self-reflection activities and goal setting tasks.   - Discuss core coaching principles and tools such as growth mindset and ADAPT framework. - **Resources:** The following resources are provided to guide participants in the application and design of their own coaching tools.   - Inpatient Structured Clinical Observation Coaching Tool: Appendix H   - Resident Self-Reflection and Goal Setting Form: Appendix I   - ADAPT Framework^23^   - Downloadable coaching observation tools: Stanford Pediatric Residency Coaching Program Website^25^ - **Guided Questions:**    - What types of coaching tools would be most helpful in your clinical and educational environment?   - What challenges have you encountered when creating space for meaningful reflection and goal setting in learners?   - How might tools be adapted to meet the needs of different learners (medical students, interns, senior residents)?   **3. Faculty Development:** (Slide 26)   - **Main Aims:**   - Discuss the implementation of core coaching journal clubs and workshops to enhance faculty development and competency in coaching.   - Summarize foundational coaching literature, including *Master Adaptive Learner^24^* and *AMA Coaching in Medical Education: A Faculty Handbook^2^* as essential resources for developing a coaching program. - **Resources:**   - Examples of downloadable faculty development workshops: Stanford Pediatric Residency Coaching Program Website.^25^ - **Guided Questions:**    - What knowledge and skills do faculty need at your institution to become effective coaches?   - How might resources like the *Master Adaptive Learner^24^* and *AMA Coaching in Medical Education^2^* support a faculty development curriculum?   - What formats (workshops, peer observation, virtual learning) would be most feasible for launching faculty development in coaching at your institution?   - What misconceptions about coaching might exist among faculty and how can targeted training clarify expectations?   **4. Evaluation and Outcomes:** (Slides 27-28)   - **Main Aims:** - Guide participants in developing their evaluation metrics based on the goals of their unique clinical coaching program. - Describe both process and outcome metrics to support evaluation of a coaching program. - Discuss potential strategies of evaluating learner outcomes, coach outcomes and program outcomes. - **Resources:** - Millers Pyramid^26^ - Kirkpatrick’s model^27^ - Example of published outcomes: Stanford Pediatric Residency Coaching Program Website^25^ - **Guided Questions:**    - What would success look like for faculty coaches and resident coachees and how can their growth or satisfaction be meaningfully assessed?   - What process measures (frequency coaching encounters, tool utilization) could help monitor implementation of a coaching program?   - How can you use outcome data to expand institutional support for coaching?   What simple tools (pre- and post-session coaching forms, focus groups, reflective journals), could you implement now to begin gathering data? |
| **Small Group Activity** | 60-90 minutes | 29 | Appendices E-I  4 tables, no more than 10 chairs per table  Printed Appendices E-I  3 laptops for tables 2-4 (optional) | Participants then engage in small working groups (30 minutes), selecting two structural components to focus on and rotating between discussion tables in 15-minute intervals. Each small working group should have a max of 10 people to encourage rich discussion. Depending on participant numbers, multiple tables may be available for each structural component. Each structural component group should be led by 1-2 workshop facilitators who have reviewed the above resources. Preferably, two facilitators for each structural component are recommended to accommodate the potential need of an additional discussion table if participation exceeds 10 members. When this workshop was presented, the small group facilitators leaders had some expertise in various stages of program development; however, this is not required to lead this workshop as resources are provided and described above. The purpose of the facilitated discussions is to provide participants with dedicated time, relevant frameworks and practical resources and tools to conceptualize their coaching programs. Facilitators will use the guided questions above for each structural component to encourage active discussion, begin addressing potential barriers, and outline preliminary steps for implementation. If additional time is available for the workshop, extra rotations can be added in the 15-minute increments, allowing participants to engage in all four structural component discussions.  **Room Setup:** A minimum of four tables is recommended for up to 10 participants per table. Each of the four tables will represent a different structural component. Participants will visit two tables during the small group activity, rotating between them while facilitators stay at their assigned table. If participant number exceeds 40, eight tables are advised to allow for two tables for each of the four different structural components.  **Materials:**   - **Table 1: Program Structure**   - Appendices E, F, G: recommended for printing and distribution to participants - **Table 2: Tools for Feedback and Facilitated Reflection**   - Appendices H, I: recommended for printing and distribution to participants   - Laptop to review coaching observation tools on Stanford Pediatric Residency Coaching Program Website^25^ (optional) - **Table 3: Faculty Development**   - *Master Adaptive Learner* book*^24^* (optional)   - *AMA Coaching in Medical Education Handbook^2^* (optional)   - Laptop to review faculty development tools on Stanford Pediatric Residency Coaching Program Website*^25^* (optional) - **Table 4: Evaluations and Outcomes**   - Laptop to review examples of published outcomes on Stanford Pediatric Residency Coaching Program Website*^25^* (optional)   Printed images of Miller’s Pyramid and Kirkpatrick’s Model (optional) |
| **Large Group Activity** | 90-100 minutes | 30 | Appendices A, B, D | Participants will report out unifying themes, key insights, identified barriers and preliminary action items for coaching program development. Participants may also report out their initial design proposals for the main structural components they worked on in small groups.  Guided Questions:   - Any shared values or goals that stood out as important for building a coaching program? - What specific challenges did you identify in developing a coaching program? - How might institutional culture or existing programs hinder coaching implementation? - How can faculty development and resident engagement impact coaching program development? - What are the most feasible and actionable initial steps to launch a coaching program? |
| **Conclusion: Commitment to Action and Post-workshop Survey** | 100-105 minutes | 31 | Appendices A, B, J  Printed out post-workshop survey for all participants | Participants will write down or share 1 SMART goal they commit to doing in the next 4 weeks to help further the development of a coaching program at their home institution. Post-workshop survey distributed for completion. (Appendix J) |

**References:**

1. Cutrer WB, Pusic MV, Gruppen LD, Hammoud MM, Santen SA, eds. *The Master Adaptive Learner.* Elsevier; 2020.
2. Coaching Program. Stanford Pediatric Residency. Accessed August 19, 2025. <https://med.stanford.edu/peds/prospective-applicants/program-information/coaching.html>
3. Pangaro L, ten Cate O. Frameworks for learner assessment in medicine: AMEE Guide No. 78. *Med Teach*. 2013;35(6):e1197-e1210. <https://doi.org/10.3109/0142159X.2013.788789>
4. Kirkpatrick DL. Techniques for evaluation training programs. *J Am Soc Training Directors.* 1959;13:21-26.
